# FANCJ DNA helicase is recruited to the replisome by AND-1 to ensure genome stability

**DOI:** 10.1038/s44319-023-00044-y

**Published:** 2024-01-02

**Authors:** Ana Boavida, Luisa MR Napolitano, Diana Santos, Giuseppe Cortone, Nanda K Jegadesan, Silvia Onesti, Dana Branzei, Francesca M Pisani

**Affiliations:** 1 Istituto di Biochimica e Biologia Cellulare, Consiglio Nazionale delle Ricerche, Naples, Italy; 2 Università degli Studi della Campania “Luigi Vanvitelli”, Caserta, Italy; 3 Structural Biology Laboratory, Elettra–Sincrotrone Trieste, Trieste, Italy; 4 IFOM ETS—The AIRC Institute of Molecular Oncology, Milan, Italy; 5 Istituto di Genetica Molecolare, Consiglio Nazionale delle Ricerche, Pavia, Italy

**Keywords:** FANCJ/BRIP1, AND-1/WDHD1, DNA Replication, Genome Stability, G-quadruplex DNA, DNA Replication, Recombination & Repair

## Abstract

FANCJ, a DNA helicase linked to Fanconi anemia and frequently mutated in cancers, counteracts replication stress by dismantling unconventional DNA secondary structures (such as G-quadruplexes) that occur at the DNA replication fork in certain sequence contexts. However, how FANCJ is recruited to the replisome is unknown. Here, we report that FANCJ directly binds to AND-1 (the vertebrate ortholog of budding yeast Ctf4), a homo-trimeric protein adaptor that connects the CDC45/MCM2-7/GINS replicative DNA helicase with DNA polymerase α and several other factors at DNA replication forks. The interaction between FANCJ and AND-1 requires the integrity of an evolutionarily conserved Ctf4-interacting protein (CIP) box located between the FANCJ helicase motifs IV and V. Disruption of the CIP box significantly reduces FANCJ association with the replisome, causing enhanced DNA damage, decreased replication fork recovery and fork asymmetry in cells unchallenged or treated with Pyridostatin, a G-quadruplex-binder, or Mitomycin C, a DNA inter-strand cross-linking agent. Cancer-relevant FANCJ CIP box variants display reduced AND-1-binding and enhanced DNA damage, a finding that suggests their potential role in cancer predisposition.

## Synopsis

FANCJ/BRIP1, a DNA helicase counteracting replication stress, is recruited to the DNA replication machinery by directly binding to AND-1. Disruption of the FANCJ/AND-1 interaction causes enhanced DNA damage, replication fork stalling and asymmetry in either unperturbed or stressful conditions.

- Human FANCJ associates with the DNA replication machinery through an interaction with AND-1 that is mediated by a Ctf4-interacting protein (CIP) box.
- The FANCJ/AND-1 interaction is important for promoting replication fork progression and suppressing DNA damage.
- Previously uncharacterized FANCJ CIP box missense mutations, found in cancer patients, reduce AND-1-binding and enhance DNA damage.

## Abstract (web summary)

FANCJ/BRIP1, a DNA helicase counteracting replication stress, is recruited to the DNA replication machinery by directly binding to AND-1. Disruption of the FANCJ/AND-1 interaction causes enhanced DNA damage, replication fork stalling and asymmetry in either unperturbed or stressful conditions.

## Graphical abstract

**Figure:**
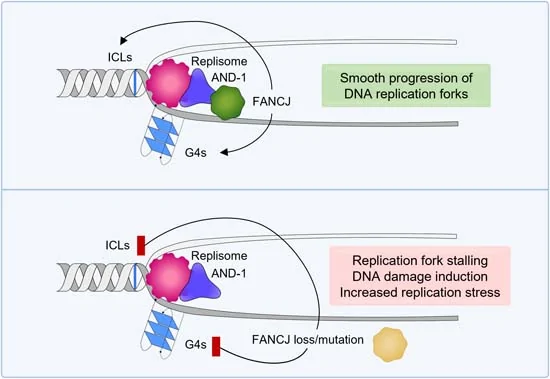

**Figure:**
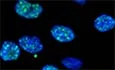

## Introduction

Faithful DNA replication is critical for genomic stability maintenance. The DNA replication machinery is continuously challenged by damaged DNA templates and other obstacles (alternate secondary structures, R-loops, tightly bound proteins) present throughout the genome. All these physical barriers, which impede a smooth progression of the replication forks, give rise to the so-called replication stress (Saxena and Zou, 2022). This condition is also caused by nucleotide pool depletion or imbalance of replicative factor levels. DNA helicases are important players in counteracting replication stress due to their ability to resolve DNA secondary structures and/or remodel nucleic acid molecules during DNA repair/recombination reactions. The importance of these enzymes in maintaining genome homeostasis is proven by the fact that many of them are genetically linked to rare hereditary diseases characterized by genome instability, chromosome anomalies, developmental defects, and cancer predisposition.

FANCJ, also known as BRIP1 (for BRCA1-interacting protein 1) or BACH1 (BRCA1-associated C-terminal helicase 1), belongs to super-family 2 (SF2) Iron–Sulfur (Fe–S) cluster-containing DNA helicases, and is frequently mutated in breast and ovarian cancers, as well as in many other tumor types (Brosh and Cantor, 2014; Cantor et al, 2001). FANCJ belongs to the Fanconi anemia pathway and bi-allelic mutations of the encoding gene are known to cause the disease, which is characterized by hematopoietic stem cell defects, progressive bone marrow failure, genomic instability and cancer predisposition (Bridge et al, 2005; Levran et al, 2005; Levitus et al, 2005; Litman et al, 2005). Human FANCJ-deficient cell lines display increased sensitivity to Mitomycin C (MMC), a genotoxic agent that introduces DNA inter-strand cross-links (ICLs). FANCJ helicase activity and direct interaction with the mismatch repair protein MLH1 are both required for processing DNA ICLs (Peng et al, 2007). Nonetheless, the precise role of FANCJ in DNA ICL repair reactions remains poorly defined. In addition to BRCA1 and MLH1, other identified FANCJ-binding partners include the DNA exo/endonuclease MRE11 (Suhasini et al, 2013), the single-stranded DNA-binding replication protein A (RPA) (Sommers et al, 2014), the Bloom DNA helicase (BLM) (Suhasini et al, 2011), the trans-lesion synthesis DNA polymerase REV1 (Lowran et al, 2020), the Topoisomerase II-binding protein TOPBP1 (Gong et al, 2010) and the DNA-end processing nuclease CtIP (Nath and Nagaraju, 2020). These multiple interactions reveal the involvement of FANCJ in various DNA repair pathways as well as in S-phase checkpoint activation.

Biochemical studies showed that the purified recombinant FANCJ protein has an ATPase-dependent DNA helicase activity with a 5’ to 3’ directionality that unwinds different DNA substrates in vitro, including DNA duplexes forming a fork or containing a 5’-flap, three-stranded displacement loops (D-loops) and various kinds of G-quadruplex (G4) DNA structures (Gupta et al, 2005; London et al, 2008; Wu and Brosh 2009). Studies carried out in different systems [worms (Cheung et al, 2002), chicken DT40 (Sarkies et al, 2012) and human cells (Bharti et al, 2013) and *Xenopus laevis* cell-free egg extracts (Castillo Bosch et al, 2014; Sato et al, 2021)] have pointed toward a prominent role of FANCJ in G4 DNA cellular metabolism. Moreover, *FANCJ*-knockout (KO) mouse embryonic fibroblasts show increased microsatellite instability, a phenotype that is further exacerbated upon treatment with compounds that induce replication stress (Matsuzaki et al, 2015; Barthelemy et al, 2016). All the above considered, it was proposed that FANCJ plays a key role in dismantling DNA secondary structures and unconventional conformations (such as G4) that occur in certain sequence contexts, especially in transiently exposed single-stranded regions. Thus, FANCJ would prevent DNA double-strand break formation and ensure a smooth progression of the DNA replication machineries in stressful conditions (Brosh and Wu, 2021). Moreover, it has been recently demonstrated that FANCJ promotes the repair of DNA-protein cross-links by actively unfolding the protein adduct thereby, enabling its cleavage by the SPARTAN protease (Yaneva et al, 2023). Notwithstanding the biological significance of FANCJ association with the DNA replication machinery, the protein factor responsible for recruiting FANCJ at the replication forks has not been yet identified.

Human acidic nucleoplasmic DNA-binding protein 1 (AND-1), also known as WD repeat and high mobility group (HMG)-box DNA-binding protein 1 (WDHD1), is an adaptor protein, crucial for DNA replication, with orthologs in metazoans and in fungi. It was originally identified in a *Saccharomyces cerevisiae* genetic screen of mutants with an increased rate of chromosome loss and was named *Ctf4* for chromosome transmission fidelity 4 (Spencer et al, 1990). Both budding yeast Ctf4 and human AND-1 were demonstrated to bridge the CDC45/MCM2-7/GINS (CMG) replicative DNA helicase with DNA polymerase α, within the replication machinery (Gambus et al, 2009; Tanaka et al, 2009; Simon et al, 2014; Kilkenny et al, 2017; Guan et al, 2017). In budding yeast, Ctf4 loss has pleiotropic effects, including increased sensitivity to genotoxic agents, defective sister chromatid cohesion and modifications in the ribosomal DNA genomic *locus* (Villa et al, 2016; Samora et al, 2016; Fumasoni et al, 2015; Sasaki and Kobayashi, 2017). Instead, the Ctf4 orthologous proteins from *Schizosaccharomyces pombe* (Williams and McIntosh, 2005), *Drosophila* (Gosnell and Christensen, 2011) and chicken (DT40 cell system) (Abe et al, 2018) are essential for cell proliferation. In human cells, AND-1 depletion using siRNAs slows down cell cycle progression (Yoshizawa-Sugata and Masai, 2009). Subsequent structural studies revealed that budding yeast Ctf4 and human AND-1 share a similar multi-domain organization, each comprising a β-propeller (WD40) and a SepB module (Simon et al, 2014; Kilkenny et al, 2017; Guan et al, 2017). However, the human AND-1 polypeptide chain has a C-terminal extension that includes an HMG DNA-binding domain, not present in budding yeast Ctf4. The SepB domain is responsible for AND-1/Ctf4 trimerization and for interaction with proteins containing the so-called Ctf4-interacting protein (CIP) box. This is a conserved short peptide sequence, originally identified in the *S. cerevisiae* DNA polymerase α catalytic subunit and in GINS Sld5, which contains conserved acidic and hydrophobic amino-acid residues essential for interacting with Ctf4 (Simon et al, 2014). Other Ctf4-binding partners were identified in budding yeast, such as the helicase–nuclease Dna2, the ribosomal DNA compaction protein Tof2, the replication initiation factor Dpb2 and the sister chromatid cohesion DNA helicase Chl1 (Simon et al, 2014; Villa et al, 2016; Samora et al, 2016). These findings suggest that Ctf4 is an interaction hub connecting the DNA replication machinery to multiple proteins and enzymes that contribute to diverse aspects of genome duplication.

Here, we report that human FANCJ directly interacts with AND-1 through a conserved CIP box that lies between the DNA helicase motifs IV and V, equivalent to the Chl1 CIP box. The FANCJ/AND-1 interaction is critical for recruiting FANCJ to the replisome and for promoting smooth progression of the DNA replication forks. We show that faulty association of FANCJ to AND-1, due to CIP box mutations, gives rise to increased DNA damage, replication fork stalling, and asymmetry in either unperturbed or stressful conditions. Besides, we report evidence that cancer-associated *FANCJ* mutant alleles causing substitutions of CIP box amino-acid residues are defective in AND-1-binding and induce enhanced DNA damage in normal and perturbed conditions. These findings provide important clues on how to interpret cancer risk and devise novel therapeutic strategies.

## Results

### Identification of a AND-1/Ctf4-interacting protein (CIP) box in FANCJ

**Figure 1:**
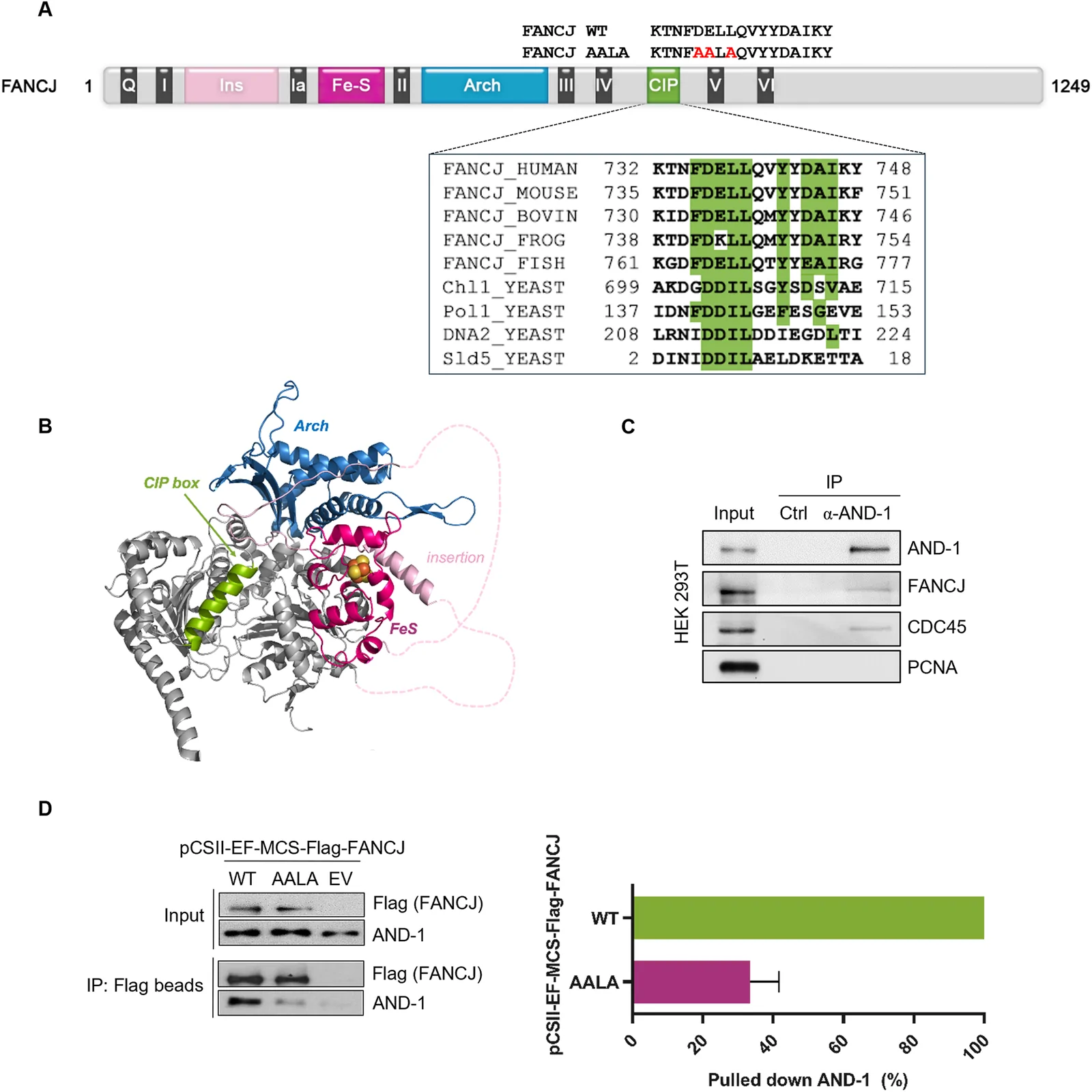
Identification of an AND-1/Ctf4-interacting protein (CIP) box in FANCJ. (**A**) Schematic representation of the polypeptide chain of human FANCJ. Conserved helicase motifs (from I to VI) are indicated in dark gray (for simplicity motifs Va and Vb are not displayed). The identified CIP box is depicted in green. Other sequence motifs and specific domains are indicated with different colors, using the abbreviations: *Q* Q motif, *Ins* Insertion, *Fe–S* Fe–S cluster, *Arch* Arch domain. A multiple sequence alignment of FANCJ and other AND-1/Ctf4-interacting proteins CIP boxes is reported in the inset. The KALIGN tool (version 3.3.1) was used. Identical or highly similar amino-acid residues are highlighted in green, if present in most of the aligned sequences. The following abbreviations are used: *HUMAN* *Homo sapiens*, *MOUSE* *Mus musculus*, *BOVIN* *Bos taurus*, *FROG* *Xenopus laevis*, *FISH* *Danio rerio*, *YEAST* *Saccharomyces cerevisiae*, Chl1 chromosome loss 1, Pol1 DNA polymerase α catalytic subunit, *Sld5* synthetic lethality with *dpb11‐1* protein 5. Changes of amino-acid residues to generate the FANCJ AALA mutant are indicated in red above the diagram. (**B**) Homology model of the FANCJ protein, as predicted by the AlphaFold server (Jumper et al, 2021), illustrating the location of the indicated conserved domains. The color code is the same as in (**A**). Disordered regions within the insertion are indicated by dashed lines; the Fe–S cluster was manually modeled based on the crystal structures of homologous DNA helicases. (**C**) Co-immunoprecipitation experiments were carried out with an anti-AND-1 antibody bound to Protein A Sepharose beads on the nuclear fraction of a S-phase-enriched HEK 293T cell population (lane indicated as *IP*). In a parallel control experiment, the anti-AND-1 antibody was omitted (lane indicated as *Ctrl*). Western blot analysis was carried out on the input (2% of each sample; 10 μg) and pulled down material (50% of each sample). Proteins of interest were detected using the indicated antibodies. PCNA was used as a negative control. The experiment was carried out in duplicate with consistent results. (**D**) Co-immunoprecipitation experiments were carried out with anti-Flag M2 beads on whole extracts from HEK 293T cells transfected with pCSII-EF-MCS vector, empty (*EV*) or expressing Flag-tagged FANCJ wild-type (*WT*) or the AALA mutant (*AALA*). Western blot analysis was carried out on the input (1% of each sample; 10 μg) and pulled-down material (50% of each sample). Proteins of interest were detected using the indicated antibodies. Co-immunoprecipitation experiments were done in triplicate. The level of immunoprecipitated AND-1 was normalized to pull down Flag-tagged FANCJ in each sample. Mean with standard errors is shown. Source data are available online for this figure.

**Figure EV1:**
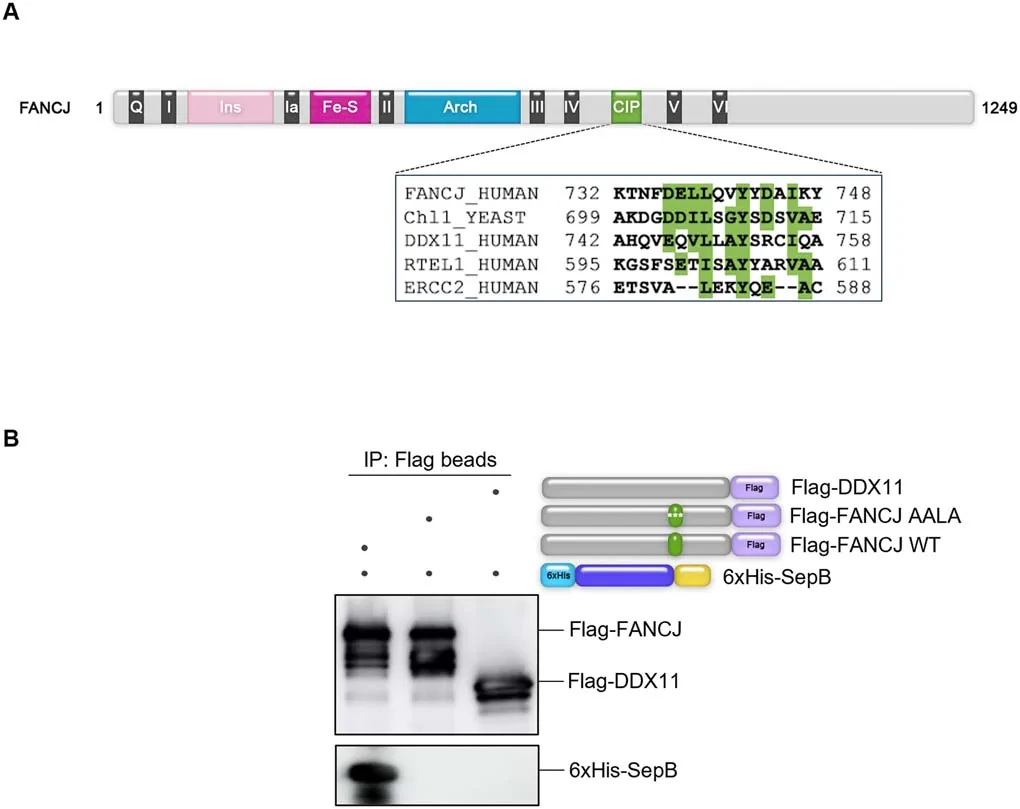
FANCJ, but not DDX11, directly interacts with AND-1 6xHis-SepB. (**A**) Schematic representation of the polypeptide chain of human FANCJ. Conserved sequence motifs and domains are indicated with the same abbreviations and colors used in Fig. 1A. In the insert, alignment of human FANCJ and budding yeast Chl1 CIP box sequence is reported with corresponding regions of other SF2 Fe–S human DNA helicases. Abbreviations used are *HUMAN*, *Homo sapiens* and *YEAST, Saccharomyces cerevisiae*. The KALIGN tool (version 3.3.1) was used. Highly conserved amino-acid residues are highlighted in green. (**B**) Co-pull-down experiments of Flag-tagged FANCJ WT or AALA mutant and AND-1 6xHis-SepB using anti-Flag agarose beads. Pulled-down samples were analyzed by Western blot with an anti-Flag peroxidase-conjugated mouse monoclonal antibody (Sigma-Aldrich cat. A8592) and anti-poly-Histidine peroxidase-conjugated mouse monoclonal antibody (Sigma-Aldrich cat. A7058). Schematic of the recombinant proteins used in the pulled-down experiments is shown.

The *S. cerevisiae* Chl1 DNA helicase was demonstrated to be recruited to the DNA replication forks through direct interaction with Ctf4. This interaction requires the integrity of a CIP box that is located between the conserved helicase motifs IV and V of the Chl1 polypeptide chain (Samora et al, 2016). Human DDX11 and FANCJ are paralogous proteins, both sharing sequence similarity with budding yeast Chl1. We identified a putative CIP box in the human FANCJ polypeptide chain between the helicase motifs IV and V by bioinformatic analysis. Other vertebrate FANCJ orthologs contain a similar putative CIP box that is located in the same position of their polypeptide chain (between the helicase motifs IV and V) and includes the amino-acid residues reported to be critical for AND-1/Ctf4-binding in other proteins (Fig. 1A) (Villa et al, 2016; Guan et al, 2017; Kilkenny et al, 2017). A multiple sequence alignment of the CIP boxes from various AND-1/Ctf4 client proteins is reported in Fig. 1A. In contrast, this putative CIP box is only partially conserved in human DDX11 or absent in other human Fe–S cluster DNA helicases (RTEL1 and ERCC2/XPD; Fig. EV1A). A model of the human FANCJ three-dimensional structure, based on an artificial intelligence prediction (Jumper et al, 2021), revealed that the putative CIP box is likely to fold as an α-helix and has a similar structure, position, and orientation as the budding yeast Chl1 CIP box (Fig. 1B).

These findings prompted us to test if FANCJ was indeed able to interact with AND-1. To this end, we carried out co-immunoprecipitation experiments on the nuclear fraction of S-phase-enriched HEK 293T cells and found that endogenous FANCJ and AND-1 are associated in cell nuclear extracts (Fig. 1C). We substituted highly conserved amino-acid residues of the putative FANCJ CIP box with Alanine to generate a FANCJ mutant named FANCJ AALA (Fig. 1A). HEK 293T cells were transiently transfected with plasmid vectors expressing Flag-tagged FANCJ wild type (WT) or the AALA mutant (AALA) and co-immunoprecipitation experiments were performed with anti-Flag M2 beads in whole-cell extracts. The results revealed that ectopically expressed Flag-tagged FANCJ WT was pulled down together with the endogenous AND-1 protein (Fig. 1D). In contrast, the amount of AND-1 co-immunoprecipitated with the Flag-tagged FANCJ AALA mutant was reduced by about 75%. All together, these results suggest that FANCJ associates with AND-1 in cell extracts, and this association is mainly dependent on the integrity of the newly identified FANCJ CIP box.

**Figure EV2:**
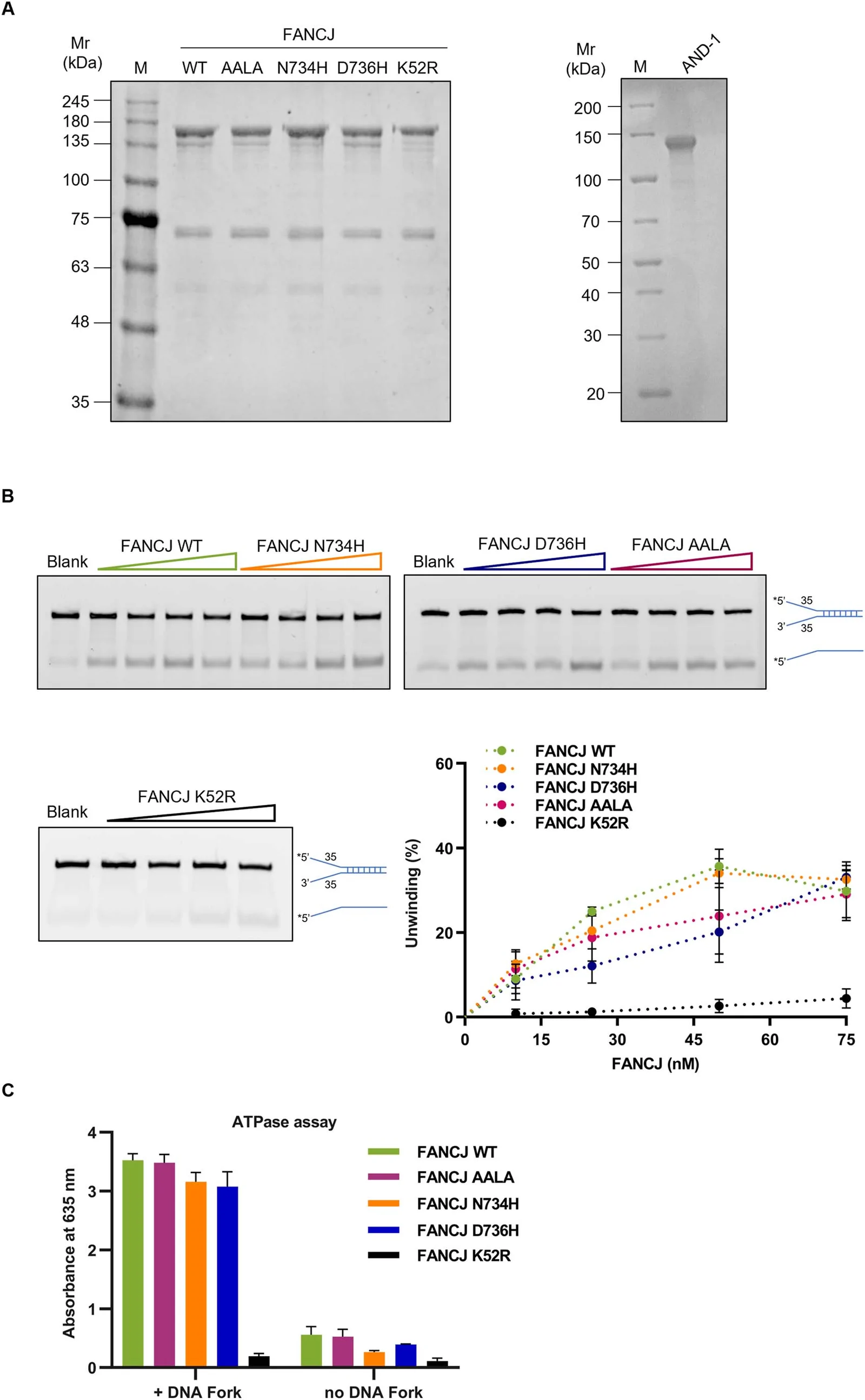
Biochemical analysis of CIP box FANCJ mutant derivatives. (**A**) SDS-PAGE analysis of purified recombinant FANCJ WT and indicated mutants and AND-1 full-length protein. An aliquot of each protein batch (3 μg) was loaded onto the indicated gel lane. Gels were stained with ProBlue Safe Stain (GiottoBiotech). Size of protein markers, loaded onto the lane indicated with *M*, is reported on the left. (**B**) DNA helicase assays using a forked duplex fluorescent-labeled DNA substrate. Asterisk represents the fluorophore attached to the 5’-end of the DNA oligonucleotide, named Fluo-D1-35. Blank refers to a control assay without protein. Data plot derives from three independent experiments (mean ± SD). (**C**) ATPase assays were carried out using the indicated FANCJ proteins (100 nM) with/without the DNA fork ligand, as described in “Methods”. Values reported derive from three independent experiments (mean ± SD).

Then, we purified Flag-tagged FANCJ WT and AALA mutant proteins from transiently transfected HEK 293T cells (Fig. EV2A) and measured their DNA helicase activity using fluorescent-labeled DNA substrates in gel-based assays. We found that FANCJ AALA retained the same level of DNA helicase activity as FANCJ WT on an anti-parallel bi-molecular G4 DNA substrate (Fig. 7B), while it was able to unwind a forked duplex DNA as efficiently as the wild-type protein only at the highest concentrations tested (Fig. EV2B). In parallel control assays, FANCJ K52R, an ATPase-dead mutant, was found to have a very low level of DNA unwinding activity, which could be ascribed to a residual catalytic function observed only at high protein concentrations. Moreover, FANCJ K52R ATPase activity was almost completely abolished and not stimulated by DNA, as observed for the ATP hydrolysis reaction catalyzed by FANCJ WT and AALA mutant in side-by-side experiments (Fig. EV2C). Therefore, these results allowed us to ascribe the observed DNA unwinding activity to the purified recombinant FANCJ WT or the AALA mutant protein and not to any co-purified contaminant DNA helicase. In addition, these findings confirmed a separation-of-function between AND-1-binding and DNA unwinding for the FANCJ AALA mutant and allowed us to exclude the possibility that the CIP box mutations altered FANCJ protein structure and/or stability and thus the loss of AND-1-binding was simply due to protein misfolding.

### FANCJ directly interacts with the AND-1 SepB domain via its CIP box

**Figure 2:**
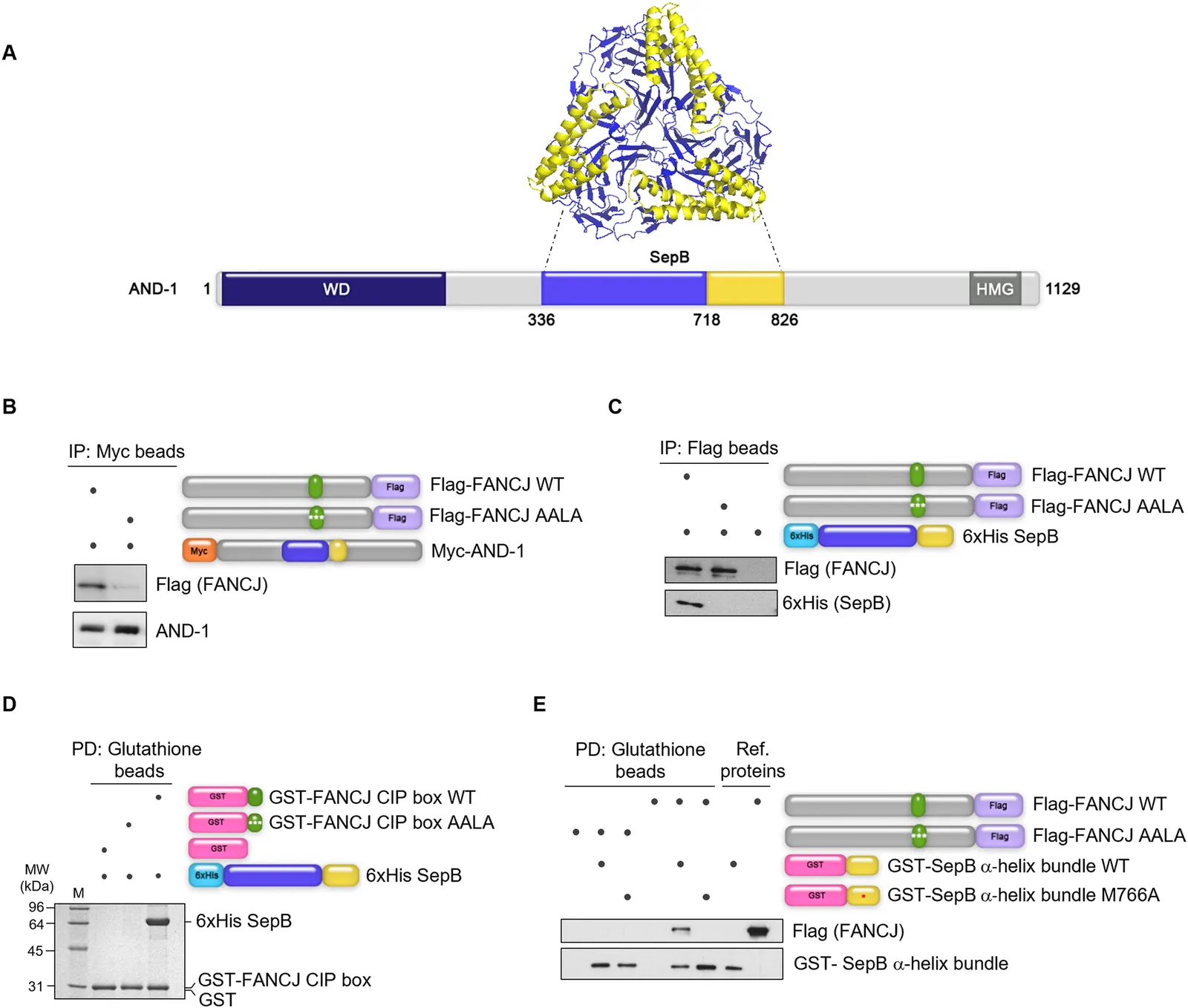
FANCJ directly interacts with AND-1. (**A**) Schematic representation of the polypeptide chain of human AND-1. The WD40 repeat (*WD*), SepB (*SepB*), and high mobility group (*HMG*) domains are indicated by boxes of different colors. The β-propeller subdomain and α-helix bundle that form the SepB domain are depicted in purple and yellow, respectively. The same colors are used to highlight them in the human AND-1 SepB structure (Protein Data Bank ID 5gvb). (**B**) Pull-down experiments of Myc-tagged AND-1 full-length and Flag-FANCJ WT or AALA mutant using anti-Myc beads. Pulled-down samples (50% of their total volume) were analyzed by Western blot with the indicated antibodies. (**C**) Pull-down experiments of Flag-tagged FANCJ WT or AALA mutant and AND-1 6xHis-tagged SepB using anti-Flag M2 beads. Pulled-down samples (50% of the total volume for each blot) were analyzed by Western blot with the indicated antibodies. (**D**) GST was fused to the FANCJ CIP box (amino-acid residues 730–747) for use in a pull-down (*PD*) assay with purified AND-1 6xHis-SepB. Specific mutations of the FANCJ CIP box (as in the GST-FANCJ CIP box AALA derivative) disrupt its ability to pull down the SepB domain. (**E**) The GST-SepB α-helix bundle chimeric protein was able to pull down purified Flag-tagged FANCJ WT. Substitution of the critical Met766 with Ala in the SepB α-helix bundle abolishes FANCJ-binding. GST-pull-down assays were carried out with the indicated recombinant proteins. Pulled-down samples (50% of their total volume) were analyzed by Western blot with the indicated antibodies. Source data are available online for this figure.

Biochemical and structural studies revealed that human AND-1 and yeast Ctf4 share a similar three-dimensional structure, formed by a N-terminal WD40 domain and a SepB module (Fig. 2A), which is responsible for AND-1/Ctf4 trimerization and for binding CIP box-containing proteins. To examine whether there was a direct interaction between FANCJ and AND-1, we carried out in vitro pull-down assays using the purified recombinant full-length proteins (Fig. EV2A). Our analysis revealed that Myc-tagged AND-1, bound to Myc-agarose beads, was able to efficiently pull down FANCJ WT, but not the FANCJ AALA mutant (Fig. 2B). When the FANCJ DNA helicase activity was assayed in the presence of full-length AND-1, neither a stimulatory nor an inhibitory effect was observed on FANCJ ability to resolve either a G4 or a forked duplex DNA substrate in vitro.

We tested the ability of FANCJ to bind the AND-1 SepB domain, produced in bacterial cells as previously described (Kilkenny et al, 2017; Guan et al, 2017), in pull-down experiments using anti-Flag beads. As shown in Fig. 2C, we found that FANCJ WT pulled down 6xHis-SepB, while the FANCJ AALA mutant was almost completely unable to bind the AND-1 SepB domain.

We next expressed a short peptide containing the FANCJ CIP box, or its AALA mutant version, as a fusion with glutathione Stransferase (GST). We observed that 6xHis-SepB was pulled down by the GST-CIP box WT protein but not by its AALA mutant form (Fig. 2D). Structural studies revealed that the SepB domain of either human AND-1 or budding yeast Ctf4 is made of a β-propeller followed by a bundle of 5 α-helixes (Simon et al, 2014; Kilkenny et al, 2017; Guan et al, 2017). The binding surface for the AND-1/Ctf4 client proteins consists of a groove between α-helices 2 and 4 (named H2 and H4) of the SepB helical bundle. The side chain of Methionine 766, which belongs to α-helix H2, is exposed on the groove surface and its substitution with an Alanine residue almost totally abolished the interaction of the SepB domain with the catalytic subunit of human DNA polymerase α, an AND-1-binding protein (Guan et al, 2017; Kilkenny et al, 2017). We produced the SepB α-helix bundle and its M766A mutant derivative as GST-fusion proteins (named GST-SepB α-helix bundle WT and M766A, respectively) and used these purified chimeric proteins to pull down FANCJ WT and the AALA mutant. As shown in Fig. 2E, a direct interaction was observed only between GST-SepB α-helix bundle WT (not the M766A mutant) and FANCJ WT (not the AALA mutant).

Collectively, these data demonstrate that FANCJ directly binds to AND-1 and this interaction requires the integrity of both the FANCJ CIP box and the AND-1 SepB α-helix bundle.

### FANCJ loss or its reduced AND-1-binding enhances DNA damage

**Figure EV3:**
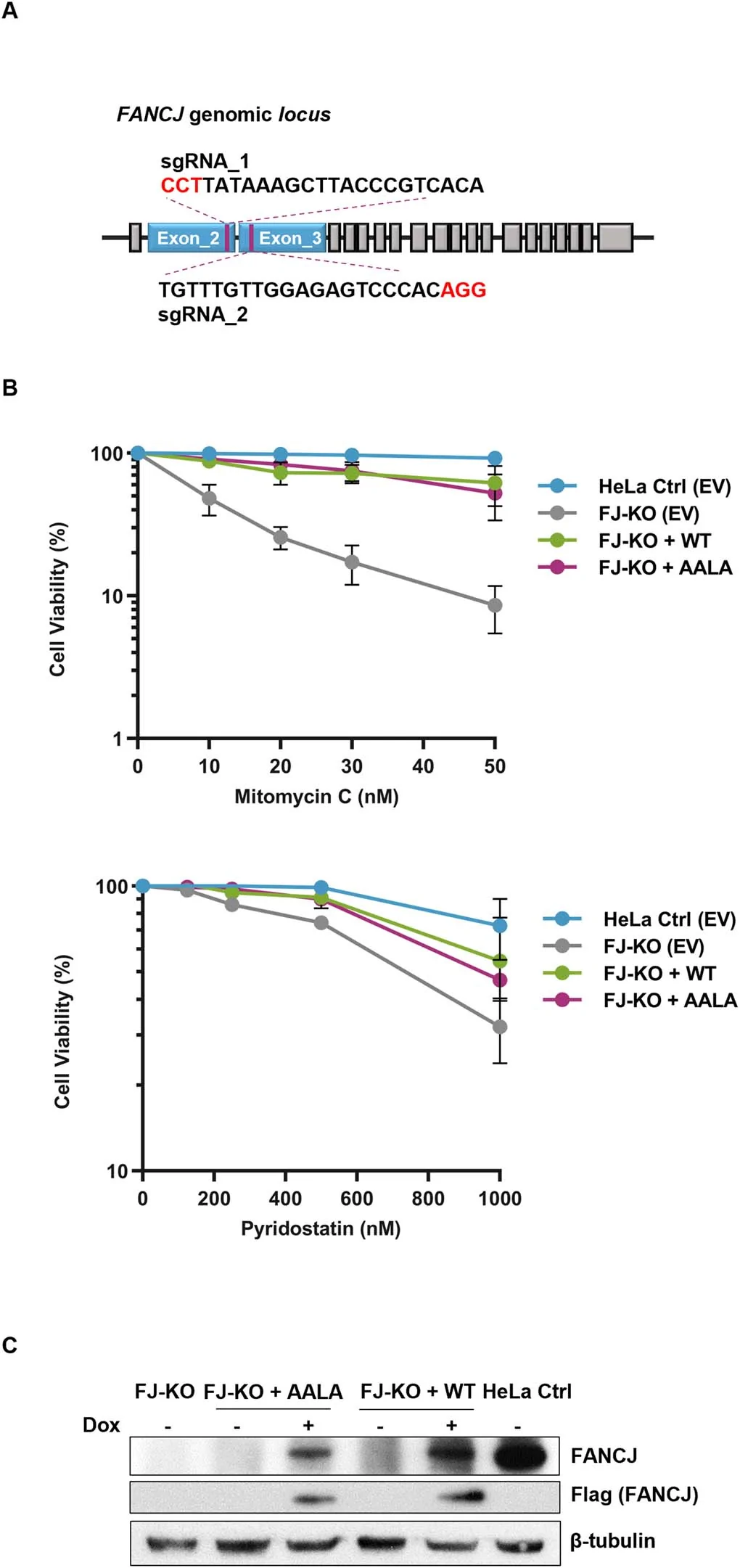
Establishment of a FJ-KO HeLa cell line and its complementation by *FANCJ* WT and AALA mutant alleles. (**A**) Schematic representation of the human *FANCJ* genomic *locus*. The sequence of CRISPR-paired guide RNAs targeting exons 2 and 3 is reported. *PAM* sequence is highlighted in red. (**B**) Viability assays of FJ-KO HeLa cell lines complemented with FANCJ WT and the AALA mutant. HeLa cells (*Ctrl*) or FJ-KO cells, transduced with lentiviral particles deriving from plasmid pCSII-EF-MCS-FANCJ WT or -FANCJ AALA or the empty vector (*EV*), were treated for 5 days with the indicated concentrations of MMC and PDS (*n*  =  3 biologically independent experiments, mean ± SD). Cells were detected by crystal violet staining, as described in “Methods”. Paired *t* tests were performed to analyze statistically significant differences between the HeLa control and the complemented cell lines, but no differences were found for both viability assays. (**C**) FJ-KO HeLa cell lines were established that stably express Flag-tagged FANCJ WT or the AALA mutant under the control of a Tetracycline-responsive promoter. Expression of ectopic *FANCJ* was detected before and after induction with Doxycycline (1 μg/mL; *Dox*) by Western blot analysis of whole extracts from the indicated cell lines using an anti-FANCJ or anti-Flag antibody. β-tubulin was used as a loading control.

To examine the physiological relevance of the FANCJ/AND-1 interaction, we carried out complementation assays in cells lacking the endogenous FANCJ protein and ectopically expressing FANCJ WT allele or its AALA mutant derivative. To this end, we generated a HeLa cell line where *FANCJ* was knocked out (FJ-KO) using CRISPR-paired guide RNAs targeting exons 2 and 3 (Fig. EV3A). Western blot analysis and Sanger sequencing of selected clones were carried out to confirm FANCJ protein loss and identify specific mutations introduced by the Cas9 activity. Since FANCJ depletion is expected to sensitize cells to Mitomycin C (MMC), a DNA inter-strand cross-linking agent, and Pyridostatin (PDS), a G4 DNA-stabilizer, we examined the viability of the FJ-KO HeLa cells after 5-day treatment with increasing doses of these drugs. As shown in Fig. EV3B, the FJ-KO HeLa cell line was sensitive to either MMC or PDS (with MMC exerting a toxic effect even in a low nM concentration range, 10–20 nM) and re-expressing FANCJ WT or the AALA mutant in the FJ-KO line did almost completely revert sensitivity to both drugs.

**Figure 3:**
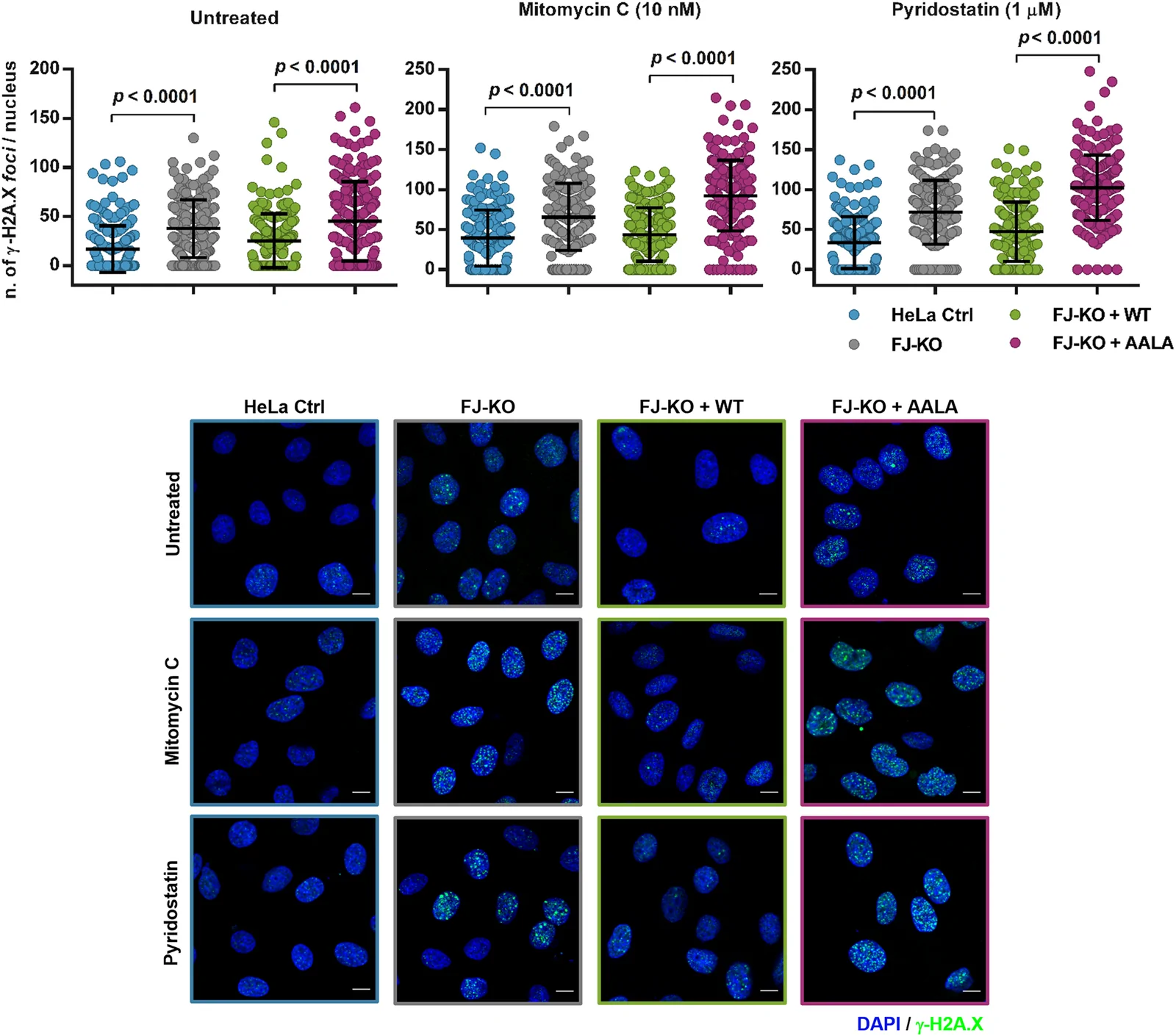
FANCJ loss or reduced binding to AND-1 enhance DNA damage. The indicated cell lines were treated with PDS or MMC, after Doxycycline induction. γ-H2A.X *focus* formation was detected by immunofluorescence with a monoclonal antibody that specifically recognizes the Ser139-phosphorylated form of the above histone. Scale bar, 10 μm. Dot plot of the number of *foci* detected per cell is reported in each graph. Bars indicate mean ± SD; 200 cells were analyzed per condition; *n* = 2 biologically independent experiments, with at least two technical replicates per experiment; two-tailed *P* value (*P* < 0.01) was calculated using Student’s *t* test nonparametric for unpaired data with Welch correction. Source data are available online for this figure.

We then stably complemented the FJ-KO HeLa line by expressing Flag-tagged FANCJ WT or the AALA mutant under the control of a Tetracycline-responsive promoter using the pMK240 plasmid vector (Okumura et al, 2018). Western blot analyses revealed that FANCJ WT and the AALA mutant were expressed at a comparable level and only upon Doxycycline induction in the selected clones (Fig. EV3C). These stably complemented cell lines were used to analyze the impact of the FANCJ/AND-1 interaction on DNA damage induction following treatment with MMC and PDS. Analysis of γ-H2A.X *foci* by indirect immunofluorescence experiments revealed a higher level of DNA damage in FJ-KO compared to control HeLa cells, not only after treatment with MMC and PDS, but also in unperturbed conditions (Fig. 3). Moreover, complementation with the *FANCJ* WT allele almost completely reverted the observed phenotype, while expression of the *FANCJ* AALA variant gave rise to an even higher level of γ-H2A.X *foci* compared to the FJ-KO cells (either with or without drug treatment; Fig. 3).

These results indicate that the FANCJ/AND-1 interaction is important for suppressing DNA damage in unchallenged conditions or after treatment with MMC or PDS. However, re-expression of either FANCJ WT or the AALA mutant rescued the ability of the FJ-KO line to survive after exposure to both drugs. These findings indicate that FANCJ interaction with AND-1 is not critical for cell viability after treatment with MMC or PDS and suggest that alternative DNA repair pathways are activated, albeit with different kinetics, to counteract enhanced DNA damage deriving from the faulty association of FANCJ to DNA replication forks.

### FANCJ associates with the DNA replication machinery through a direct interaction with AND-1

**Figure 4:**
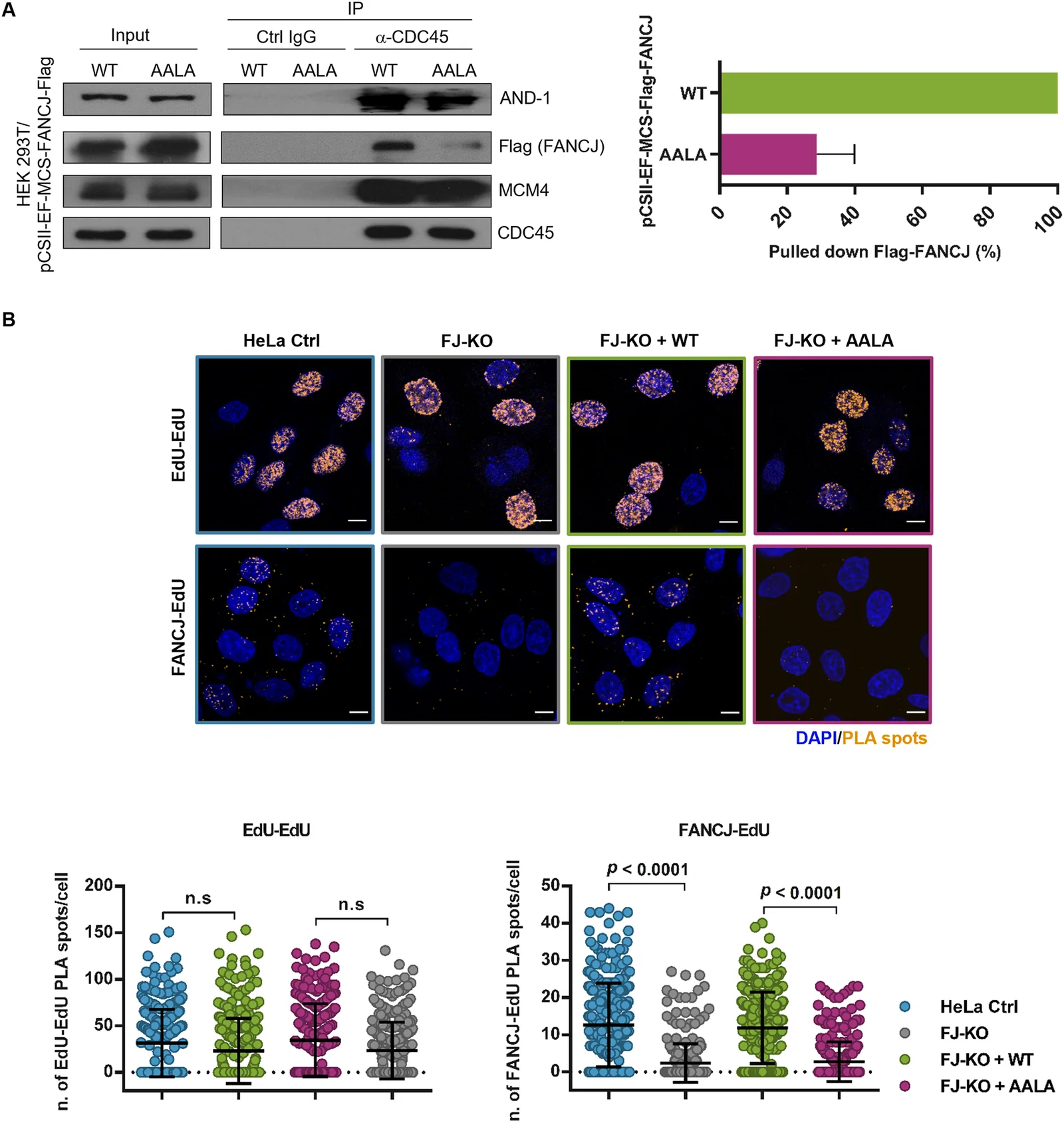
FANCJ associates to DNA replication forks through a direct interaction with AND-1. (**A**) Co-immunoprecipitation experiments were carried out on the nuclear fraction of HEK 293T cells transiently transfected with pCSII-EF-MCS plasmid constructs expressing Flag-tagged FANCJ wild-type (*WT*) or AALA mutant (*AALA*). Control (*Ctrl IgG*) and anti-CDC45 rabbit IgG (*α-CDC45*), bound to Protein A Sepharose beads, were added to each indicated nuclear fraction. Western blot analysis was carried out on the input (2% of each sample; 10 μg) and pulled-down material (50% of each sample). Proteins of interest were detected using the indicated antibodies. Immunoprecipitated protein samples were analyzed by Western blot using the indicated antibodies. Data from triplicate experiments show level of immunoprecipitated FANCJ (mean ± SD), normalized to pulled-down endogenous CDC45, in each sample. Means with standard errors are shown. (**B**) FANCJ association to sites of DNA synthesis was analyzed by SiRF assays. EdU-EdU (upper part) or FANCJ-EdU (lower part) proximity ligation assay (PLA) spots were quantified from control (*Ctrl*), FJ-KO and complemented HeLa cell lines. Scale bar, 10 μm. The frequency distribution of the population was analyzed and two-tailed *P* value (*P* < 0.01) was calculated using Student’s *t* test nonparametric for unpaired data with Welch correction (< 300 cells per condition; *n* = 3 biologically independent experiments; bars show mean ± SD). Source data are available online for this figure.

Given that FANCJ directly binds to AND-1 *via* its CIP box and AND-1 is a component of the DNA replication machinery, we asked whether FANCJ localizes at DNA replication forks. Co-immunoprecipitation experiments were carried out using an antibody specific for CDC45, a component of the CMG complex, to identify proteins associated with the DNA replication machinery in cell extracts. HEK 293T cells, transiently transfected with plasmids expressing Flag-tagged FANCJ WT or the AALA mutant, were released in S phase after an overnight thymidine block. The nuclear fraction was prepared from these cells using a procedure that included a sonication step followed by an exhaustive nuclease treatment to remove nucleic acids that could mediate protein-protein associations. Co-immunoprecipitations were performed in the nuclear fraction samples using an anti-CDC45 antibody conjugated to Protein A Sepharose beads. As reported in Fig. 4A, Western blot analyses revealed that FANCJ WT was pulled down with CDC45, together with AND-1 and MCM4, a subunit of the MCM2-7 complex; in contrast, the amount of FANCJ AALA mutant co-immunoprecipitated with CDC45 was reduced by ~75% in experiment replicates. Since CDC45 is loaded onto chromatin only in S phase as a stable component of the CMG complex (Masai et al, 2010), our results revealed that FANCJ was anchored to the DNA replication machinery *via* a direct interaction with AND-1, as this interaction was dependent on the integrity of the FANCJ CIP box.

Thereafter, we examined the localization of FANCJ to DNA synthesis sites marked by EdU incorporation in FJ-KO HeLa cell lines complemented with *FANCJ* WT or AALA mutant allele using the in situ visualization of protein interactions at DNA replication forks (SiRF) technique (Roy et al, 2018). Control experiments revealed a comparable level of EdU-EdU proximity ligation assay (PLA) spots in all the cell lines tested, meaning that they had a similar number of active replication factories and an equal probability to produce a positive signal in the conditions used for the SiRF assay (Fig. 4B). Our analyses revealed the presence of robust FANCJ-EdU PLA signals either in HeLa control cells or in the FJ-KO line complemented with the *FANCJ* WT allele, indicating that FANCJ does co-localize with DNA synthesis sites. In contrast, FANCJ-EdU PLA spots were almost totally absent either in FJ-KO cells or in the FJ-KO line complemented with the FANCJ AALA mutant, suggesting that the CIP box AALA mutation significantly reduced the level of FANCJ associated to the DNA replication factories (Fig. 4B).

Collectively, these results reveal that FANCJ is associated with the DNA replication machinery *via* a direct interaction with AND-1. Our findings are consistent with a previous study, where nascent chromatin capture (NCC), used to profile chromatin proteome dynamics during S phase in human cells, indicated that FANCJ is among the proteins that are enriched at the replication forks in nascent chromatin (Alabert et al, 2014). Besides, in a more recent report the isolation of proteins on nascent DNA (iPOND) technique revealed the presence of FANCJ at replication forks in unchallenged conditions (Peng et al, 2018). However, although all these data would suggest that FANCJ is a constitutive component of the DNA replication machinery, the possibility that its recruitment at the replication forks is induced by endogenous DNA damage or replication fork barriers cannot be ruled out.

### AND-1-binding by FANCJ promotes replication fork progression in stressful conditions

**Figure 5:**
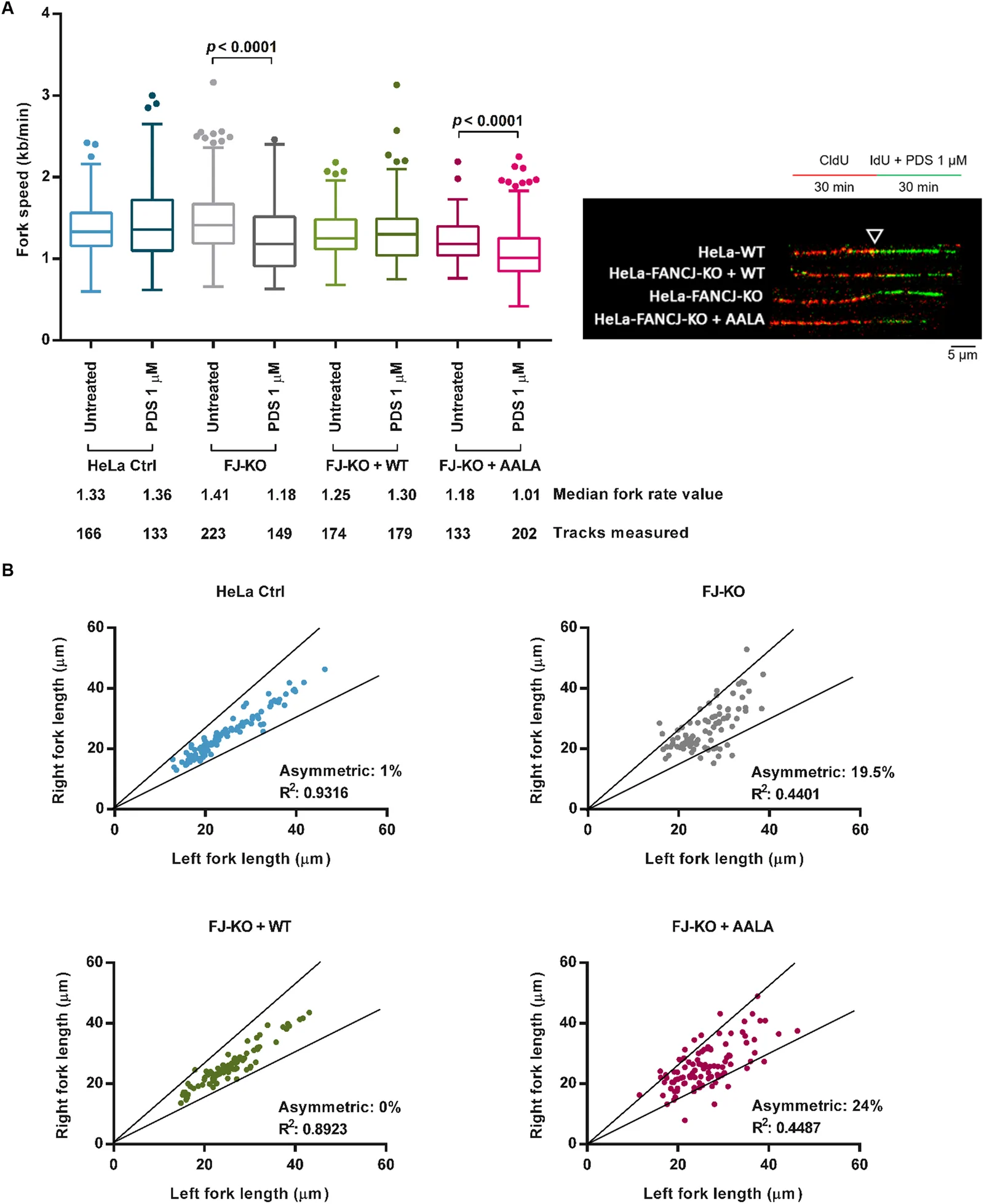
Association of FANCJ to the DNA replication machinery promotes fork progression in challenged conditions. (**A**) DNA fiber track analysis was carried out in either untreated or perturbed conditions. Box-plot showing fork speed determined on CldU:IdU double-labeled DNA fibers from control (*Ctrl*), FJ-KO and stably complemented HeLa cell lines, untreated or treated with PDS (1 µM) simultaneously with IdU pulse (*n*  =  3 biologically independent experiments, with at least 2 technical replicates). Labeled DNA fibers are shown from a representative experiment (arrow represents the switch point from CldU to IdU labeling). Median fork rate and the number of tracks analyzed are shown. The box extends from the 25th minus 1.5×IQR to 75th plus 1.5×IQR percentiles—Tukey method. Two-tailed *P* values were calculated by Mann–Whitney *U* test. (**B**) Fork asymmetry analysis in cells treated with PDS during IdU pulse, as in (**A**). The central area between the lines delimits a variation of <25% in fiber length. *R*^2^ is the linear correlation coefficient. Approximately 100 sister forks (green-red-green tracks only) were analyzed and plotted from three biologically independent experiments. Source data are available online for this figure.

Next, we examined the effect of disrupting the FANCJ CIP box on the replication fork dynamics using DNA fiber track assays to monitor DNA replication at a single-molecule level in the aforementioned cell lines. As schematically depicted in Fig. 5A, cells were first labeled with CldU and then a second label, IdU, was administered without or with PDS to test replication fork progression in normal or challenging conditions, respectively. A significant reduction of replication fork speed was measured upon PDS treatment in FJ-KO cells and in FJ-KO cells complemented with the AALA mutant, with a more prominent effect observed in this latter cell line (Fig. 5A). Analysis of sister forks (forks emanating from the same replication origin), obtained in PDS-treated conditions, revealed that the percentage of those with asymmetric tracks was remarkably higher in either FJ-KO cells or in FJ-KO cells complemented with the AALA mutant, indicating that replication forks stalled more frequently when FANCJ is either absent or not stably anchored to the ongoing replisomes (Fig. 5B).

### Cancer-relevant FANCJ CIP box mutations reduce AND-1-binding and induce enhanced DNA damage

**Figure 6:**
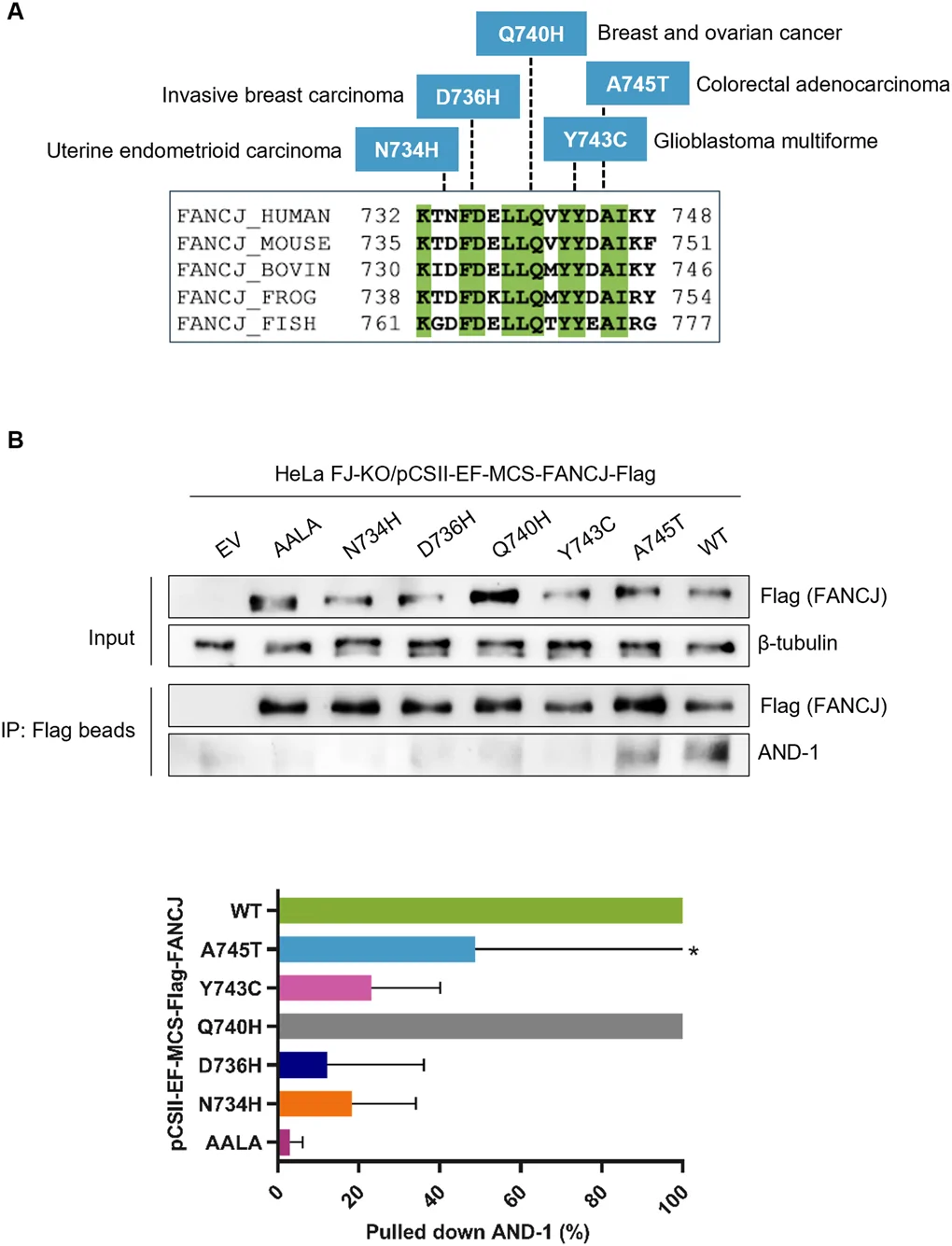
Cancer-associated FANCJ CIP box mutations show reduced AND-1-binding in cells. (**A**) Cancer-relevant FANCJ CIP box mutants. Amino-acid changes corresponding to *FANCJ* missense variants found in the indicated tumors are shown in the upper part. A multiple sequence alignment of the CIP box from various FANCJ orthologs is reported in the lower part; highly conserved amino-acid residues are highlighted in green. The following abbreviations were used: *HUMAN* *Homo sapiens*, *MOUSE* *Mus musculus*, *BOVIN* *Bos taurus*, *FROG* *Xenopus laevis*, *FISH* *Danio rerio*. (**B**) Co-immunoprecipitation experiments with anti-Flag agarose beads were carried out on whole extracts of HeLa FJ-KO cells transiently transfected with pCSII-EF-MCS vector expressing Flag-tagged FANCJ wild type (*WT*) or the indicated mutants (*EV* stands for empty vector). Western blot analysis was carried out on the input (1% of each sample; 5 μg of protein) and pulled-down material (20 and 40% of each sample for FANCJ-Flag and AND-1 detection, respectively). Proteins of interest were detected using the indicated antibodies. Experiments were done in triplicate. The level of immunoprecipitated endogenous AND-1 was quantitated in each sample, as described in “Methods”. Mean ± SD are shown in the bar graph, asterisk on A745T bar refers to a standard deviation of 58.44. Source data are available online for this figure.

After *BRCA1* and *BRCA2*, *FANCJ* is the third most common ovarian cancer susceptibility gene: nearly 0.9– 2.5% of all ovarian cancer patients carry a splice-site, stop, or frameshift mutation in the *FANCJ* gene (Ramus et al, 2015; Norquist et al, 2016). Besides, *FANCJ* was found to be mutated in several other malignancies, including melanoma, breast, prostate, and hereditary colon cancer, suggesting that *FANCJ* mutations may be a risk factor in multiple tumor types (Cantor and Guillemette, 2011; Paulo et al, 2018; Ali et al, 2019). Nevertheless, a direct association of *FANCJ* mutations with predisposition to breast cancer has been questioned (Weber-Lassalle et al, 2018; Easton et al, 2016). However, as the majority of *FANCJ* clinical variants remain uncharacterized, their possible connection to cancer risk is difficult to interpret (Moyer et al, 2020; Calvo et al, 2021). Through data mining of cancer genomics databases (cBioPortal, http://cbioportal.org, Cosmic-3D, https://cancer.sanger.ac.uk/cosmic3d, and gnomAD, https://gnomad.broadinstitute.org) we pinpointed *FANCJ* variants expected to give rise to mutations of CIP box amino-acid residues in various malignancies, including uterine endometrial carcinoma (N734H), invasive breast carcinoma (D736H), breast and ovarian cancer (Q740H), glioblastoma multiforme (Y743C), and colorectal adenocarcinoma (A745T) (Fig. 6A).

To test if the above FANCJ mutant derivatives were able to bind AND-1, we carried out co-immunoprecipitation experiments with anti-Flag agarose beads in whole extracts of FJ-KO HeLa cells transiently transfected with plasmids expressing Flag-tagged versions of the FANCJ mutants of interest. As reported in Fig. 6B, the FANCJ CIP box mutants N734H and D736H displayed a reproducibly reduced association with the endogenous AND-1 protein, as also found for the FANCJ AALA mutant.

**Figure 7:**
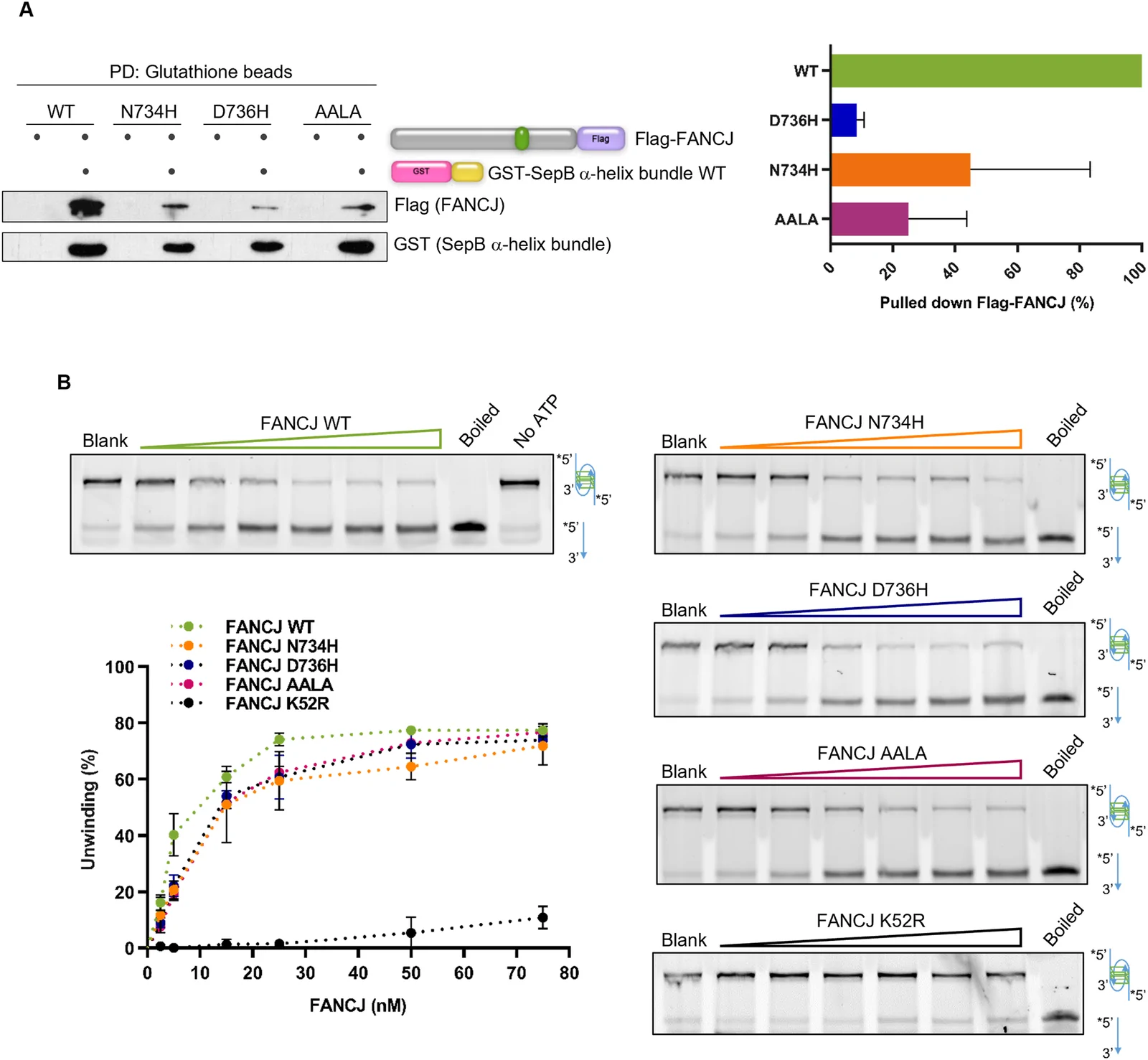
Biochemical characterization of the cancer-related FANCJ CIP box mutants N734H and D736H. (**A**) GST-pull-down experiments of the GST-SepB α-helix bundle protein and Flag-tagged FANCJ WT, AALA, N734H, or D736H mutants. Reduced binding to SepB α-helix bundle is observed for all the FANCJ variants, compared to the wild-type protein. Mean ± SD are shown in the bar graph (*n*  =  3 independent experiments). (**B**) Gel-based DNA helicase assays were carried out using FANCJ WT or the indicated mutants at increasing concentrations and a fluorescent-labeled anti-parallel bi-molecular G4 DNA substrate. Asterisk represents the fluorophore attached to the DNA oligonucleotide 5’ end. Blank and No ATP refer to control assays without protein or ATP, respectively. Boiled refers to a heat-denatured assay mixture with no protein. Graphs report data of three independent experiments (mean ± SD) carried out as described in the text. Source data are available online for this figure.

The FANCJ N734H and D736H mutants were produced as Flag-tagged recombinant proteins and purified from transiently transfected HEK 293T cells (Fig. EV2A). Then, GST-pull-down assays were carried out to examine their direct interaction with the GST-SepB α-helix bundle chimeric protein. Results from these experiments confirmed that AND-1-binding by both these FANCJ mutants was reduced, even if not completely abolished (Fig. 7A).

When assessing the activity towards a G4 DNA substrate, the FANCJ N734H and D736H mutants displayed the same level of unwinding as the wild-type protein (Fig. 7B). When tested towards a canonical forked duplex DNA, FANCJ N734H was as efficient as FANCJ WT, while the D736H mutant was as active as FANCJ WT on this substrate only at the highest concentrations tested (Fig. EV2B). In parallel control assays, when ATP was omitted from the assay, FANCJ WT did not display a detectable helicase activity on either a G4 or a forked duplex DNA substrate. In contrast, FANCJ K52R, an ATPase-dead mutant, was found to have a very low level of DNA unwinding, which could be ascribed to a residual catalytic function observed only at high protein concentrations, and not to another contaminating DNA helicase. In agreement with this hypothesis, FANCJ K52R was found to possess a very low level of ATPase activity that was not stimulated in the presence of DNA. In contrast, the ATPase activity of FANCJ WT and its mutants of interest was found to be highly enhanced in the presence of DNA in side-by-side experiments (Fig. EV2C). Therefore, our results confirmed previously published data indicating that FANCJ DNA helicase function is ATP-dependent and allowed us to ascribe the observed DNA unwinding activity to the purified recombinant FANCJ WT protein or its CIP box mutants of interest and not to any co-purified contaminant protein. Overall, these findings confirmed a separation-of-function between AND-1-binding and DNA helicase activity for the FANCJ N734H and D736H CIP box mutants, as also previously found for the FANCJ AALA mutant.

**Figure EV4:**
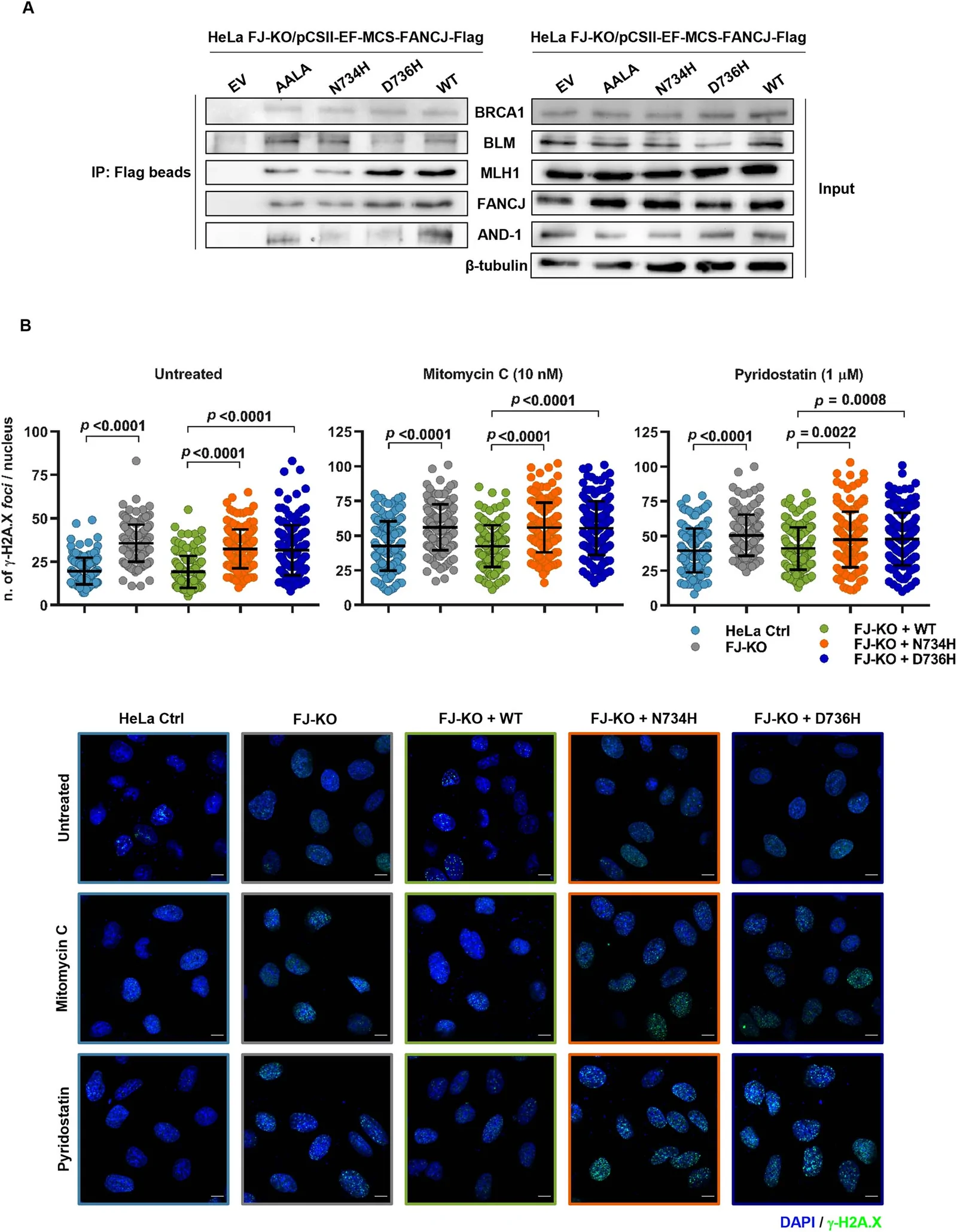
FANCJ CIP box mutants, AALA, N734H and D736H retain the ability to interact with BRCA1, BLM and MLH1 and induce enhanced DNA damage. (**A**) Co-immunoprecipitation experiments with anti-Flag agarose beads were carried out on whole extracts of HeLa FJ-KO cells transiently transfected with pCSII-EF-MCS vector expressing Flag-tagged FANCJ wild type (*WT*) or the indicated mutants (*EV* stands for empty vector). Western blot analysis was carried out on the input (0.6% of each sample; 10 μg of total protein) and pulled-down material (50% of each sample). Proteins of interest were detected by Western blot experiments using the indicated antibodies. Experiments were done in triplicate. (**B**) HeLa FJ-KO cells, transfected with plasmid vectors expressing FANCJ WT or the indicated mutants, were treated with PDS or MMC. γ-H2A.X *focus* formation was detected by immunofluorescence with a monoclonal antibody that specifically recognizes the Ser139-phosphorylated form of the above histone. Scale bar, 10 μm. Dot plot of the number of *foci* detected per cell is reported in each graph. Bars indicate mean ± SD; 150 cells were analyzed per condition; *n* = 2 biologically independent experiments, with at least two technical replicates per experiment; two-tailed *P* value (*P* < 0.01) was calculated using Student’s t test nonparametric for unpaired data with Welch correction.

Moreover, we found that the above FANCJ CIP box mutants retain the ability to associate with BRCA1, BLM, and MLH1, other known FANCJ-binding partners in cell extracts (see Fig. EV4A), suggesting that their involvement in the related genome maintenance pathways is not affected by the above CIP box amino-acid substitutions.

Then, we examined the effect of the FANCJ N734H and D736H mutants on DNA damage induction in cells untreated/treated with MMC or PDS. This analysis revealed that the above FANCJ CIP box mutants were unable to counteract increased γ-H2A.X *focus* formation in FJ-KO HeLa cells (see Fig. EV4B).

## Discussion

Here, we report the identification of an AND-1/Ctf4-interacting protein (CIP) box in the human FANCJ polypeptide chain between the conserved DNA helicase motifs IV and V. We found that endogenous FANCJ and AND-1 are associated in cell extracts and demonstrated that a direct interaction takes place between the two proteins produced in purified recombinant form. Moreover, pull-down experiments with various AND-1 and FANCJ protein fragments revealed that the FANCJ CIP box directly binds to the α-helix bundle of the AND-1 SepB domain (Fig. 2). Notably, only some of the CIP box residues were found in the same region of the DDX11 DNA helicase sequence and we were unable to detect any direct interaction between Flag-tagged DDX11 and AND-1 SepB in co-immunoprecipitations carried out in vitro with the purified recombinant proteins (Fig. EV1), in agreement with a previous report (Farina et al, 2008).

Whereas the majority of CIP boxes fold as short α-helices that are typically surrounded by partially disordered regions, the *S. cerevisiae* Chl1 CIP box is located on an α-helix that is an integral part of the structure, although reasonably exposed to the solvent. As previously pointed out, in an AlphaFold model of the human FANCJ structure, the proposed CIP box is exactly structured and positioned as the budding yeast Chl1. When compared with the CIP boxes present in low complexity sequences (such as those found in human Pol α and in budding yeast Sld5, Pol1, and DNA2), some of the residues that are expected to interact with AND-1/Ctf4 (i.e., FANCJ L738 and L739 and Chl1 I705 and L706) are partly buried. However, the CIP box α-helices of both FANCJ and Chl1 appear to be loosely associated with the bulk of helicase structure, and it is not impossible to conceive a small conformational change that would expose those residues to the solvent. Moreover, no experimental structure for either FANCJ or Chl1 is available, and our bioinformatic analyses rely on computational models that are designed to place hydrophobic residues toward the inside of the protein; the presence of conserved hydrophobic amino acids may therefore drive the algorithm to artificially bury those residues. Indeed, the interaction between budding yeast Chl1 and Ctf4 is supported by a growing body of evidence (Borges et al, 2013; Samora et al, 2016; Villa et al, 2016), strongly suggesting that the conformation of the Chl1 CIP box α-helix is not an impediment. Nevertheless, an alternative explanation would be that FANCJ (or Chl1) binds AND-1 (or Ctf4) in a different fashion involving other residues within the same region.

The physiological relevance of the FANCJ/AND-1 interaction was examined by functional assays carried out in FJ-KO HeLa cells stably complemented with either *FANCJ* WT or the AALA mutant allele. The FANCJ AALA derivative is a separation-of-function mutant as it is defective in AND-1-binding due to changes in critical CIP box residues but retains a level of DNA helicase activity comparable with the wild-type protein. Of note, we found that the CIP box mutations described in this study (AALA, N734H, and D736H) did not abolish the ability of FANCJ to associate with BRCA1, BLM, and MLH1, suggesting that the related FANCJ functions could be not altered by these amino-acid changes (see Fig. EV4A). Our data revealed that the integrity of the CIP box was critical for recruiting FANCJ to the replication factories and for suppressing DNA damage in either unperturbed or challenged conditions (treatment with MMC or PDS). In addition, we found that the FANCJ/AND-1 interaction protected DNA replication forks by promoting their progression and stability, especially in cells treated with PDS. These findings are consistent with several lines of evidence suggesting that FANCJ has a prominent role in resolving G4 structures at DNA replication forks (Cheung et al, 2002; Wu et al, 2008; Schwab et al, 2013; Castillo Bosch et al, 2014; Sato et al, 2021; Van Schendel et al, 2021) and with biochemical studies showing that FANCJ is able to untangle different kinds of G4 DNA structures in vitro with higher catalytic efficiency, as compared to other DNA helicases (Bharti et al, 2013). Of note, G4 *foci* were reported to accumulate in FANCJ-deficient human cells, revealing that other G4 resolvases only partially compensate for FANCJ loss (Henderson et al, 2014; Summers et al, 2021). Recent studies based on multi-color single-molecule localization microscopy have provided direct evidence that in human cells G4 are formed at DNA replication forks behind the CMG complex and resolved by the combined action of FANCJ and replication protein A (RPA) (Odermatt et al, 2020; Lee et al, 2021). In recent structural studies of the human CMG complex bound to AND-1 (Rzechorzek et al, 2020) or the whole human core replisome (Jones et al, 2021), the SepB trimer appears to be located near-perpendicularly to the N-tier face of the MCM2-7 complex, where it interfaces with CDC45 and GINS via its β-propeller domain, while the SepB α-helix bundle and the C-terminal HMG subdomain project away from the CMG, likely in close proximity to the lagging strand. Thus, FANCJ, bound to the SepB helical bundle, occupies a position in the replisome favorable to promptly hop onto the lagging strand between the Okazaki fragments and/or the unwound leading template as it emerges from the MCM complex, where G4 DNA structures are expected to arise. Notably, in a study carried out in DT40 cells it was suggested that G4 occurring at the replication forks could be resolved by the interplay of DDX11 and its binding partner TIMELESS, a component of the fork-protection complex (Lerner et al, 2020). In fact, in this cell system DDX11 and TIMELESS were found to act epistatically in preventing G4-dependent epigenetic instability, but independently from FANCJ. In agreement with these findings, herein we propose the existence of at least two partially redundant pathways responsible for G4 resolution at the DNA replication forks in human cells: one operating on the DDX11-TIMELESS axis and the other one involving FANCJ and its binding partner AND-1 (Calì et al, 2015; Cortone et al, 2018; Lerner et al, 2020).

Furthermore, our data suggest that the FANCJ/AND-1 interplay could also be relevant for repairing DNA ICLs. The integrity of the Fanconi anemia pathway is required to fix these DNA lesions and allow restart of stalled DNA replication forks. In a very recent work, it has been demonstrated that ATR-mediated phosphorylation of AND-1 at Threonine 826 (an amino-acid residue of the SepB helical bundle) promotes recruitment of the FANCM/FAAP24 complex at ICL-stalled DNA replication forks (Zhang et al, 2022). Of note, the authors of this study have proposed that AND-1 initiates the Fanconi anemia pathway by sensing and specifically binding DNA ICLs through its C-terminal HMG domain: threonine 826 phosphorylation would elicit an AND-1 conformational change that enhances affinity of the HMG domain for DNA ICLs promoting FANCM/FAAP24 recruitment to these damaged sites.

Our study also addresses the possible functional significance of two *FANCJ* missense variants (*FANCJ* N734H and D736H), that are found in tumor patients and result in mutations of CIP box amino-acid residues (Moyer et al, 2020; Calvo et al, 2021). We observed that AND-1-binding by these cancer-related FANCJ mutants is severely compromised, while the DNA helicase activity and the interaction with other known protein binding partners (BRCA1, BLM and MLH1; see Fig. EV4A) is not altered, as compared to the wild-type protein. Moreover, the FANCJ N734H and D736H mutants were found to be unable to counteract DNA damage induced by treatment with PDS or MMC in FJ-KO HeLa cells, likewise the FANCJ AALA mutant (see Fig. EV4B). Our findings offer a possible interpretation of the functional significance of these clinically relevant *FANCJ* variants, showing that the association of FANCJ to the DNA replication machinery *via* AND-1 is crucial for preserving genomic integrity in either normal or perturbed conditions. Further analysis evaluating tumor frequency in *FANCJ*-knocked-in mouse models is required to assess impact and prognostic relevance of the above cancer-associated *FANCJ* variants.

## Methods

### Plasmid construction and protein expression and purification

Plasmid pCSII-EF-MCS (version 3.4) harboring the human Flag-tagged FANCJ open reading frame (ORF) was a gift from Hisao Masai (Tokyo Metropolitan Institute of Medical Science, Tokyo, Japan) (Uno et al, 2012). The FANCJ mutant alleles, named AALA and N734H, D736H, Q740H, Y743C, A745T, and K52R, were produced by a PCR-mediated site-directed mutagenesis protocol (Carey et al, 2013) using the Q5 DNA polymerase (New England Biolabs Laboratories) or KOD Hot start (Merck). Plasmids (pCSII-EF-MCS version 3.4) expressing human Flag-tagged FANCJ WT and its mutant forms, named AALA, N734H, D736H, and K52R, were transfected into HEK 293 T cells grown on ten 15-cm dishes. After 48 h, cells were detached using ice-cold PBS (150 mM NaCl, 2.7 mM KCl, 10 mM Na_2_HPO4, 1.8 mM KH_2_PO4, pH 7.5) and collected by centrifugation (250× *g*, 3 min, 4 °C). Cell pellets were resuspended in Binding Buffer (50 mM Tris-HCl, pH 7.0, 150 mM NaCl, 10% [v:v] glycerol, 0.1% [v:v] Triton X-100 and 2× cOmplete EDTA-free protease inhibitor cocktail [Roche]). Cell lysis was obtained by a mechanical pestle and sonication, following digestion with Benzonase (Merck) at 25 units/mL (in the presence of 5 mM MgCl_2_) for 30 min. The sample was ultracentrifuged (21500× *g*, 45 min, 4 °C), the supernatant filtered with 0.22-μm filter and mixed with anti-Flag M2 agarose beads (Merck) for 2 h in a rotating wheel at 4 °C. Resin was washed with Binding Buffer containing increasing amounts of NaCl (150 mM, 300 mM, and 500 mM). Bound protein was eluted from the resin using Elution Buffer (50 mM Tris-HCl, pH 7.0, 300 mM NaCl, 10% [v:v] glycerol, 0.1% [v:v] Triton X-100, 0.2 µg/µL 3× Flag-peptide, 2× cOmplete EDTA-free protease inhibitor cocktail [Roche]). The eluted sample was concentrated on a Vivaspin (cutoff: 10 kDa). Sample buffer was exchanged by diafiltration in Storage Buffer (25 mM Tris-HCl, pH: 7.5, 150 mM NaCl, 10% [v:v] glycerol, 1 mM PMSF, and 1 mM dithiothreitol. The final yield of the purified full-length proteins ranged from about 50 to 150 μg from a total of 9 × 10^7^ HEK 293T-transfected cells.

The pFBDM vector plasmid harboring the ORF encoding full-length 10xHis-Myc-tagged AND-1 (amino-acid residues 1–1129; a gift of Luca Pellegrini, Cambridge, United Kingdom) was used to generate a recombinant baculovirus (Kilkenny et al, 2017). To express the AND-1 protein, High-Five^TM^ insect cells, grown in suspension at a concentration of 1.0 × 10^6^ cells/mL (culture volume: 1 L), were infected with a recombinant baculovirus stock and incubated for 72 hr at 27 °C. Then, cells were harvested by centrifugation (1000× *g*, 1 hr, 4 °C), washed with PBS supplemented with 10% (v:v) glycerol and stored at −80 °C. After thawing, cells were resuspended in ten volumes of Lysis Buffer (25 mM Tris-HCl, pH 7.2, 300 mM NaCl, 10% [v:v] glycerol, 0.5 mM Tris(2-carboxyethyl)phosphine [TCEP], 20 mM imidazole), complemented with protease inhibitors (cOmplete, EDTA-free) and incubated for 15 min on ice. Cells were disrupted by 5 × 10-s pulses of ultrasound at 35% amplitude interrupted by 45-s incubations on ice using a Branson 450 Digital Sonifier. Benzonase (Merck) was added at 2.40 units/mL and, after incubation at room temperature (RT) for 15 min, the crude extract was centrifuged (30000× *g*, 4 °C, 1 h). A 1-mL aliquot of Ni-NTA resin (Qiagen), pre-equilibrated with Lysis Buffer, was added to the supernatant. The mixture was incubated at 4 °C for 2 h. The resin was collected in a 20-mL column (bed volume: 2 mL) and washed with Lysis Buffer (volume: 30 mL). Resin was further washed with Lysis Buffer containing 5 mM MgCl_2_, 0.5 mM ATP (10 column volume, CV), followed by an additional wash with Lysis Buffer (10 CV). The AND-1 protein was eluted in 5-mL fractions with Elution Buffer (25 mM Tris-HCl, pH 7.2, 300 mM NaCl, 10% [v:v] glycerol, 0.5 mM TCEP, 250 mM imidazole). The sample was diluted in Elution Buffer without NaCl so that the salt concentration was reduced to 100 mM. Then, the sample was applied to a 1-mL HiTrap Q column (Cytiva), pre-equilibrated with anion exchange buffer (25 mM Tris-HCl pH, 7.2, 100 mM NaCl, 10% [v:v] glycerol, 0.5 mM TCEP). AND-1 was eluted with a 20-CV gradient from 150 to 1000 mM NaCl. Peak fractions were pooled and concentrated using an Amicon Ultra-15 system (cutoff: 30 kDa).

Recombinant human 6xHis-tagged AND-1 SepB (amino-acid residues 336–826) was produced in *Escherichia coli* BL21 (*DE3*) cells transformed with pRSF-Duet-1 plasmid construct (a gift from Luca Pellegrini, Cambridge, United Kingdom) and purified, as previously described (Kilkenny et al, 2017).

The FANCJ CIP box (amino-acid residues 730–747) wild-type (WT) and its AALA mutant version were produced as GST-fused proteins by cloning the encoding ORF sequences into the pGEX-6P-1 plasmid (GenScript).

The SepB α-helix bundle (amino-acid residues 718–824) was produced as a GST-fused protein by cloning the encoding ORF into the pGEX-2T plasmid. The recombinant chimeric protein was expressed in *E. coli* Rosetta p*LysS* (*DE3*) cells, grown in LB medium at 37 °C. When the A_600_ of the culture reached 0.6 optical density (OD), the cell culture was cooled down at 20 °C, and isopropyl thio-D-galactopyranoside (IPTG) was added at 0.1 mM. After an overnight incubation at 20 °C, cells were collected by centrifugation and resuspended in Binding Buffer (PBS containing: 2× cOmplete EDTA-free protease inhibitor cocktail [Roche], 1 mg/mL Lysozyme, 25 units/mL Turbonuclease and 5 mM MgCl_2_). Cells were broken by sonication and incubated for 20 min on ice, in a shaking platform, to allow further digestion of nucleic acids by Turbonuclease. Thereafter, NaCl concentration was adjusted to 300 mM and the sample was ultracentrifuged (21500 *g*, 45 min, 4 °C). The supernatant was filtered through a 0.22-μm filter and mixed with Glutathione Sepharose 4B agarose beads (Cytiva). The sample was incubated for 1 h in a rotating wheel at 4 °C. Resin was washed with Binding Buffer (PBS supplemented with 150 mM NaCl) and protein was eluted with Elution Buffer (50 mM Tris-HCl, pH 7.5, 300 mM NaCl and 10 mM reduced glutathione). The eluted sample was concentrated using a Vivaspin system (cutoff: 10 kDa). Sample buffer was exchanged by diafiltration in Storage Buffer (50 mM Tris-HCl, pH: 7.5, 300 mM NaCl, 1 mM PMSF and 1 mM dithiothreitol).

All proteins were stored in aliquots at −80 °C.

### Co-immunoprecipitation experiments from whole-cell extract

pCSII-EF-MCS plasmids expressing Flag-tagged FANCJ WT or the mutants of interest were transfected into HEK 293T (shown in Figs. 1D, 4A, and 6B) using poly-ethylenimine (PEI, Polyscience, Inc.). At 48 h *post* transfection, cells (about 1 × 10^9^ cells/experiment) were detached, and collected by centrifugation (250× *g*, 3 min, RT). After two washes in ice-cold PBS, cell pellets were resuspended in Lysis Buffer (50 mM Tris-HCl, pH 8.0, 150 mM NaCl, 0.25% [v:v] Triton X-100, 10% [v:v] glycerol) supplemented with cOmplete EDTA-free protease inhibitor cocktail (Roche). The samples were subjected to sonication on ice (eight cycles consisting of 2-s impulses at an output 15% followed by 5-s intervals), followed by incubation for 20 min at 37 °C in the presence of Benzonase (25 units/sample) and MgCl_2_ (1 mM). Insoluble material was removed by centrifugation (13000× *g*, 10 min, 4 °C). Then, Flag M2 agarose beads (Merck; volume: 20 μL) were added to cell extract aliquots containing 1–2 mg of total protein. Samples were incubated at 4 °C for 2 h in a rotating wheel. Beads were finally washed four times with Lysis Buffer and pulled-down proteins were eluted by adding SDS-PAGE loading buffer 1.5× (100 mM Tris-HCl, pH 6.8, 20% [v:v] glycerol, 400 mM β-mercapto-ethanol, 1.0% [w:v] SDS, 0.02% [w:v] blue bromophenol) to each pelleted resin sample. Mixtures were incubated at 95 °C for 5 min and subjected to Western blot analysis using the indicated antibodies. Relative Western blot band quantification was performed using the ImageJ software (1.52 v) by analyzing raw grayscale images (8-bit) of three biological replicates of the same experiment performed in the same conditions. The gray mean value of each band and background was measured using the rectangle tool by defining a region of interest that was fitting all the bands. The pixel density for all data (bands and backgrounds) was inverted by subtracting the gray mean value from 255 (pixel value in 8-bit images). The net value (amount) was calculated by deducting the inverted background from the inverted band value. When the amount of each band is calculated, AND-1 pulled-down values were normalized to each FANCJ pulled-down values (amount of pulled-down AND-1 divided by the amount of pulled-down FANCJ in each lane). Using this value, to plot the graph, an average of three biological replicates was calculated. The average value was converted to percentage assuming FANCJ WT average value as being 100%.

### In vitro pull-down assays

The pull-down experiments to analyze the interaction of FANCJ with full-length AND-1 (shown in Fig. 2B) were performed in pull-down (PD) Buffer (PBS containing 5% [v:v] glycerol, 0.5 mM TCEP, 0.2% [v:v] Igepal), as previously described (Kilkenny et al, 2017). Saturating quantities of purified His_10_-Myc-tagged AND-1 (12 μg) were added to c-Myc magnetic beads (Pierce^TM^, cat 88842, volume: 20 μL). The beads were washed three times with PD Buffer. Then, purified FANCJ WT or AALA mutant (4 μg) were added. Following a 2-h incubation at 4 °C in the presence of Benzonase (2.40 units/mL; Merck), the beads were washed four times with PD Buffer. Finally, they were resuspended in SDS-PAGE loading buffer supplemented with 2 mM DTT (volume: 30 μL) and boiled for 10 min at 95 °C. Samples were analyzed by western blot using an anti-Myc mouse monoclonal antibody (ab32, Abcam) and HRP-conjugated anti-Flag antibody (A8592, Merck).

Direct interaction between recombinant purified human AND-1 SepB and FANCJ proteins (shown in Figs. 2C and EV1B) was analyzed by co-immunoprecipitation experiments. Flag M2 agarose beads (Merck; volume: 10 μL) were incubated with Flag-tagged FANCJ WT or AALA mutant (2 µg of each purified recombinant protein) in mixtures (final volume: 300 µL) containing pull-down (PD) Buffer 1 (20 mM HEPES-NaOH, pH 7.2, 150 mM NaCl, 5% [v:v] glycerol, 1 mM dithiothreitol, 0.1% [v:v] Igepal) supplemented with cOmplete EDTA-free protease inhibitor cocktail (Roche). Samples were incubated for 1 hr at 4 °C in a rotating wheel. Then, they were washed three times with PD Buffer 1 containing 1% (w:v) BSA and incubated in the same buffer (final volume: 200 µL) for 20 min at 4 °C in a rotating wheel. Purified AND-1 SepB (4 µg) was added to the indicated samples in PD Buffer 1 containing 1% (w:v) BSA supplemented with cOmplete EDTA-free protease inhibitor cocktail (Roche; final volume: 250 µL). Samples were incubated for 1 h at 4 °C in a rotating wheel. Then, samples were washed twice with PD Buffer 1 containing 1% (w:v) BSA and protease inhibitor cocktail and four times with PD Buffer 1. Pulled-down proteins were eluted by adding SDS-PAGE loading buffer 2× (100 mM Tris-HCl, pH 6.8, 20% [v:v] glycerol, 400 mM β-mercapto-ethanol, 1.0% [w:v] SDS, 0.02% [w:v] blue bromophenol) to each pelleted resin sample. Mixtures were incubated at 95 °C for 5 min and subjected to western blot analysis using the indicated antibodies.

GST-pull-down assay to examine the interaction between GST-FANCJ-CIP box and the AND-1 SepB domain (shown in Fig. 2D) were carried out essentially as previously described (Villa et al, 2016). Transformed cells were cultured in LB medium (culture volume: 25 mL) at 37 °C until the A_600_ of the culture reached 0.9–1.0 OD. Expression of the fusion protein was induced by adding IPTG at 0.5 mM to the medium. Cell culture was incubated for 20 h at 16 °C. Cells were centrifuged (3500× *g*, 30 min, 4 °C). The cell pellet was resuspended in Lysis Buffer (50 mM Tris-HCl, pH 7.0, 500 mM NaCl, 10% [v:v] glycerol, 1 mM dithiothreitol) complemented with cOmplete EDTA-free protease inhibitor cocktail (Roche). After cell disruption by sonication, the cell extract was clarified by centrifugation (30000× *g*, 1 h, 4 °C). The soluble extract was then mixed with Glutathione Sepharose 4B beads (50 μL; Cytiva) pre-equilibrated in the same buffer and incubated under rotation at 4 °C for 1 h. Unbound protein was removed by three consecutive washes with Lysis Buffer (1 mL), followed by three washes (1 mL) with PD Buffer 1 containing BSA at 1% [w:v]. Then, the purified AND-1 SepB protein (0.5 mg in 500 μL) was added to the beads and binding was allowed to take place by incubating the mixtures for 1 h at 4 °C under rotation. Thereafter, the beads were washed three times with PD Buffer 1 and washed again three times with the same buffer without BSA. Proteins were eluted by adding SDS-PAGE loading buffer 2× to each pelleted resin sample. Mixtures were incubated at 95 °C for 5 min and subjected to SDS-PAGE followed by Coomassie-staining.

GST-pull-down assays (reported in Figs. 2E and 7A) were carried out to examine the interaction between GST-SepB α-helix bundle and full-length FANCJ WT and mutants of interest. Glutathione Sepharose 4B beads (10 μL) were incubated with GST-SepB α-helix bundle (0.5 µg) in mixtures containing PD Buffer 2 (25 mM Tris-HCl, pH 7.5, 150 mM NaCl and 0.1% [v:v] Triton X-100). Samples were incubated for 1 h at 4 °C in a rotating wheel and washed three times with PD Buffer 2. Then, purified Flag-tagged FANCJ WT and the indicated mutants were added (0.5 µg) to the mixtures containing the beads. Samples were incubated for 1 hr at 4 °C under rotation and finally washed five times with PD Buffer 2 containing NaCl at 300 mM and Triton X-100 at 1% (v:v). Pulled-down proteins were eluted by adding SDS-PAGE loading buffer 2× to each resin pellet. Mixtures were incubated at 95 °C for 5 min and subjected to SDS-PAGE followed by Coomassie-staining or Western blot analysis.

### Generation of a *FANCJ*-knockout (KO) HeLa cell line by CRISPR/Cas9

To establish a FJ-KO line, HeLa cells (ATCCCCL-2) were transiently transfected with SpCas9-expressing pX459 plasmid (Addgene # 62988) using Lipofectamine 2000 (ThermoFisher Scientific). Forty-eight hours *post* transfection, Alt-R synthetic paired guide RNAs (Integrated DNA Technologies), targeting *FANCJ* exon 2 (TATAAAGCTTACCCGTCACA) and exon 3 (TGTTTGTTGGAGAGTCCCAC), were transfected using Lipofectamine RNAiMAX Transfection Reagent (ThermoFisher Scientific) and expanded for clonal populations. These latter were screened using western blot analysis to detect FANCJ protein expression and further confirmed by Sanger sequencing. For sequencing, *FANCJ* genomic *loci* were amplified using high-fidelity Q5 DNA polymerase (New England Biolabs), and amplified fragments were subcloned using the Zero Blunt Topo PCR cloning kit (Invitrogen). Plasmid DNA was isolated from at least 10–15 colonies and sequenced to identify frameshifts or deletions produced by the Cas9 activity. *CRISPRscan* (https://www.crisprscan.org/) and *CRISPOR* (http://crispor.tefor.net/) online tools were used to design guide RNAs.

### Establishment of stable FJ-KO HeLa cell lines expressing FANCJ WT and the AALA mutant

For establishing FJ-KO HeLa cell lines that express FANCJ WT or the AALA variant, HeLa FJ-KO cells were transfected with the following plasmids: pMK204-TetOne AAVS1-MCS (+) Flag-tagged FANCJ WT or pMK240-TetOne AAVS1-MCS (+) Flag-tagged FANCJ AALA (GenScript). The plasmid pMK240-TetOne AAVS1-MCS (+), was a gift from Masato Kanemaki (National Institute of Genetics, Mishima, Japan) (Okumura et al, 2018). After transfection, cells were cultured in 96-well plates and selected by adding Puromycin (0.3 μg/mL) to DMEM complete medium. Single clones were isolated and further expanded in the presence of Puromycin at a lower concentration (0.2 μg/mL). Expression of Flag-tagged FANCJ, WT and AALA mutant, was assessed before and after induction with Doxycycline (1 μg/mL) by western blot analysis using the indicated antibodies.

All cell lines were tested for mycoplasma contamination and maintained at 37 °C with 5% CO_2_.

### Cell viability assays

To complement FJ-KO cells, lentiviral particles were produced in HEK 293T by transfecting the pCSII-EF-MCS empty vector (as a control), pCSII-EF-MCS-FANCJ WT and pCSII-EF-MCS-FANCJ AALA individually, together with psPAX2 and pMD2.G. For cell viability assays, HeLa FJ-KO cells, transduced with lentiviral particles, were seeded at a concentration of 1000–1500 cells/well in 96-well plates. Cells were treated with the indicated concentrations of PDS and MMC for 5 days (chronic treatment). Then, after discarding the medium, wells were washed with PBS, and viable cells were detected by adding a crystal violet solution (0.5% [w:v] dissolved in 20% [v:v] methanol) and incubating the plates for 5 min at RT. After removing the crystal violet solution, plates were washed with milli-Q water and dried overnight at RT. Then, absorbance at a wavelength of 570 nm was read in each well using a multi-well plate reader.

### Co-immunoprecipitation experiments from cell nuclear fraction

The association between endogenous FANCJ and AND-1 (as shown in Fig. 1C) was analyzed in HEK 239T cells, cultured in 10-cm dishes. Cells were synchronized in S phase with a single block in thymidine (2 mM) followed by the release in fresh medium for 2.5 h. Preparation of cell nuclear fraction was performed according to a published protocol with modifications (Guillou et al, 2010). Cell pellets were resuspended in 1 mL of Osmotic Buffer (10 mM HEPES-NaOH, pH 7.9, 0.2 M potassium acetate, 0.34 M sucrose, 10% [v:v] glycerol, 1 mM dithiothreitol, 0.1% [v:v] Triton X-100) and incubated for 5 min on ice. After centrifugation (800× *g*, 5 min, RT), the nucleus/chromatin fraction present in the pellet was resuspended in 1 mL of Hypotonic Buffer (10 mM HEPES-NaOH, pH 7.9, 50 mM NaCl, 1 mM dithiothreitol, 0.1% [v:v] Triton X-100) supplemented with cOmplete EDTA-free protease inhibitor cocktail (Roche). Samples were subjected to sonication on ice (ten cycles consisting of 10-s impulses at an output 10% followed by 20-s intervals) followed by incubation for 20 min at 37 °C in the presence of Benzonase (25 units/sample) and MgCl_2_ (1 mM). Insoluble material was removed by centrifugation (16000× *g*, 30 min, 4 °C). Samples (containing 0.5 mg of protein) were used in immunoprecipitation experiments with an anti-AND-1 antibody (H00011169-M01, Novus) bound to Protein A Sepharose beads (Cytiva). Mixtures were incubated for at least 3 hr at 4 °C in a rotating wheel. Beads were washed four times with Washing Buffer (10 mM HEPES-NaOH, pH 8.0, 50 mM NaCl, 1 mM dithiothreitol, 0.1% [v:v] Triton X-100). Proteins bound to the beads were eluted with SDS-PAGE loading buffer, incubated at 95 °C for 5 min, and analyzed by Western blot using the indicated antibodies.

Co-immunoprecipitations (as shown in Fig. 4A) using a CDC45 antibody bound to Protein A Sepharose beads were carried out on nuclear extracts prepared from HEK 293 T cells (about 4 × 10^7^ cells/experiment), transiently transfected with the indicated plasmid constructs for FANCJ WT and AALA. After transfection, cell culture synchronization and preparation of cell nuclear fractions was performed as above described.

### Immunofluorescence

For analyzing γ-H2A.X *focus* formation, HeLa cells (*Ctrl*, FJ-KO and complemented cell lines; experiments shown in Fig. 3) were grown overnight on coverslips in six-well plates (2 × 10^5^ cells/well), induced with Doxycycline (1 μg/mL) for 24 h and further treated for 24 h with PDS (1 μM) or MMC (10 nM). After incubation, coverslips were fixed in cold methanol for 5 min and further blocked/permeabilized for 1 h at room temperature with PBS containing: 1% [w:v] BSA, 0.3 M glycine, 0.1% [v:v] Tween20. Coverslips were incubated overnight with rabbit monoclonal anti-γ−H2A.X (phospho S139; 1:250, clone EP854(2)Y, Abcam) in a humid chamber at 4 °C. After washing with PBS, coverslips were incubated for 1 h at room temperature with goat anti-rabbit Alexa Fluor™ 488 (1:500, ThermoFisher Scientific) as a secondary antibody. Thereafter, coverslips were mounted into glass slides using mounting media containing DAPI. Images were acquired with a confocal microscope (Zeiss LSM 700) using a ×63 magnification objective (Nikon). γ-H2A.X *foci* were quantified with ImageJ (1.52 v) using the Find Maxima tool with variable values of prominence depending on each experiment: each cell was analyzed, and quantification (number of *foci* identified) made by the software was also checked and confirmed by eye-inspection.

For analyzing γ-H2A.X *focus* formation (experiments shown in Fig. EV3A), HeLa Ctrl and FJ-KO cells were seeded on coverslips in six-well plates (2 × 10^5^ cells/well) and grown overnight. The subsequent day, cells were transfected with the indicated pCSII-EF-MCS plasmids expressing FANCJ WT and its N734H or D736H mutant forms. Transfection was carried out using 1 μg of each plasmid and 3 μL of X-tremeGENE 9 DNA transfection reagent (Roche). In parallel control experiments, carried out in the same conditions but with a pcDNA plasmid expressing eGFP, transfection efficiency was estimated to be not lower than 90%, as revealed by visualizing cells that emitted green light, due to the eGFP presence, versus the total number of cells, observed under a bright field. At 24 h *post* transfection, cells were treated with PDS (1 μM) or MMC (10 nM) for 24 h. After incubation, coverslips were treated, as described before, to detect γ-H2A.X *foci* by indirect immunofluorescence. Thereafter, coverslips were mounted into glass slides using mounting media containing DAPI. Images were acquired with a confocal microscope (Zeiss LSM 980) using a ×63 magnification objective (Nikon) and analyzed with ImageJ (1.52 v), as above described.

### SiRF assays

Quantitative in situ analysis of protein interactions at DNA replication forks (SiRF) assays was carried out as previously described (Roy et al, 2018). HeLa cells (Ctrl, FJ-KO, and stably complemented cell lines) were incubated with Doxycycline (1 μg/mL) for 24 hr before seeding on eight-well chamber slides (Lab-Tek), at ~15,000 cells/well. Cells were pulsed with EdU (125 µM) for 15 min, followed by incubation with hydroxyurea (HU, 4 mM) for stalling DNA replication forks. Cells were subjected to fixation with a solution containing 2% [v:v] paraformaldehyde in PBS for 15 min at room temperature. Then, cells were permeabilized with a solution containing 0.25% [v:v] Triton X-100 in PBS for 15 min at RT, followed by washing with PBS. EdU-biotinylation was performed with a click-it reaction cocktail (PBS containing 2 mM CuSO_4_, 10 µM biotin-azide, 100 mM sodium ascorbate). Slides were incubated with primary antibodies, mouse anti-biotin (clone BTN.4, ThermoFisher Scientific), and rabbit anti-FANCJ (B1310, Merck). Specific probes that cross-react with the primary antibodies were used for rolling circle amplification and the related SiRF signals were visualized as discrete PLA (proximity ligation assay) spots in cell nuclei that were also counter-stained with DAPI. Images were acquired with a confocal microscope (Zeiss LSM 700) using a 63x magnification objective (Nikon). Signals were quantified using ImageJ software (1.52 v) and the Find Maxima tool with variable values of prominence depending on each experiment. Each cell was analyzed and quantification (number of points identified), done using the software, was also verified by eye inspection. Control experiments were also carried out to detect sites of DNA synthesis in cell nuclei by EdU-EdU PLA spot analysis for the same lines.

### DNA fiber track assays

Cells were pulse-labeled with chloro-deoxyuridine (CldU, 20 μM) for 30 min, followed by incubation in iodo-deoxyuridine (IdU, 200 μM) for 30 min. For the experiments where a PDS treatment was used, the drug was administered at 1 μM to the cells together with IdU. Approximately 1750 cells were lysed with 7.5 μL of Lysis Buffer (200 mM Tris-HCl, pH 7.5, 50 mM EDTA, 0.5% [v:v] SDS). Fibers were spread on Superfrost microscope slides (Epredia, AG00008332E01MNZ10), which were tilted by ~45° and air-dried for 2 min. Slides were then fixed in a mixture of methanol:acetic acid (3:1, v-v). After drying, or on the next day, DNA was denatured using a solution of HCl (2.5 M) for 1 h. After a careful wash with PBS, slides were incubated with filtered Blocking Solution (PBS containing 1% [w:v] BSA) for 1 h at room temperature. Slides were incubated with a rat anti-BrdU antibody (1:200, clone BU1/75 (ICR1), Abcam) overnight at 4 °C. Next day, slides were washed with PBS and incubated with goat anti-rat antibody labeled with Alexa Fluor™ 647 (1:500, ThermoFisher Scientific). Thereafter, slides were incubated with a mouse anti-BrdU antibody (1:500, clone B44, BD Biosciences), followed by incubation with a goat anti-mouse antibody labeled with Alexa Fluor™ 555 (1:500, ThermoFisher Scientific), both at room temperature for 1 h. Slides were mounted with mounting media containing DAPI (PBS:glycerol, 1:1 [v:v]). Images of DNA fibers were taken with a confocal microscope (Zeiss LSM 700) using a ×63 magnification objective (Nikon). Fiber tract lengths were assessed with ImageJ and μm values were converted into DNA kilobases using the conversion factor 1 µm = 2.59 kb for the spreading technique (Quinet et al, 2017; Nieminuszczy et al, 2016). Sister forks derived from the same fiber were also measured for fork asymmetry analysis.

### DNA helicase assays

PAGE-purified oligonucleotides used for the preparation of DNA substrates were purchased from Merck. An anti-parallel bi-molecular G4 DNA substrate was prepared using the following fluorescent-labeled DNA oligonucleotide OX1 (5’-GACCACTG-[Cy3]T-CGGTTCCAAGCACTGTCGTACTTGATATTTTGGGGTTTTGGGG-3’), as previously described (Calì et al, 2015). DNA helicase assays were carried out in Helicase Assay Buffer (25 mM HEPES-NaOH, pH 7.5, 5 mM MgCl2, 25 mM KCl, 2 mM dithiothreitol, 0.1 mg/mL [w:v] BSA), containing 2 mM ATP and 1.5 nM DNA substrate. Reactions (volume = 20 μL) were initiated by the addition of the indicated proteins. Then, samples were incubated for 20 min at 37 °C. Reactions were quenched with the addition of 5 µL of 5× Stop Solution (0.5% [w:v] SDS, 40 mM EDTA, 0.5 [w:v] mg/mL proteinase K, 20% [v:v] glycerol). Samples were run on a 8% polyacrylamide-bis (29:1) gel in TBE containing 0.1% [w:v] SDS at a constant voltage of 100 V. Both gel and running buffer contained KCl at 10 mM to preserve G4 DNA structure.

For the preparation of the forked duplex DNA substrate, fluorescent-labeled Fluo-D1-35 oligonucleotide (5′-[6FAM] GCACTGGCCGTCGTTTTACGGTCGTGACTGGGAAAACCCTGGCGTTTTTTTTTT-3′) was annealed to a 3-fold molar excess of the complementary unlabeled D3-35 oligonucleotide (5’-TTTTTTTTTTTTTTTTTTTTTTTTTTTTTTTCCCAGTAAAACGACGGCCAGTGC-3’; the complementary sequences are underlined) by incubation at 95 °C for 5 min and gradually cooled to 25 °C. DNA helicase assays were carried out in Helicase Assay Buffer containing 2 mM ATP, 20 nM DNA substrate and 100 nM capture strand oligo, named Cap1 (5’-GCACTGGCCGTCGTTTTAC-3’). Reactions (volume = 20 μL) were initiated by the addition of the indicated proteins and then incubated for 20 min at 37 °C. Samples were quenched with the addition of 5 µL of 5× stop solution. Samples were run on a 8% polyacrylamide-bis (29:1) gel in TBE containing 0.1% [w:v] SDS at a constant voltage of 100 V.

After electrophoresis, gels were analyzed with an imaging system instrument (ChemiDoc, Bio-Rad Laboratories). The displaced oligonucleotide was quantified and any free oligonucleotide in the absence of enzyme was subtracted.

### ATPase assay

The ATPase/GTPase activity assay kit (Sigma-Aldrich, cat. MAK113) was used to measure the presence of inorganic phosphate release from ATP as a measure of FANCJ ATPase activity. The assay mixture (volume: 50 μL) had the following composition: 25 mM HEPES-NaOH pH 7.2, 5 mM MgCl_2_, 50 mM KCl, 2 mM DTT, 0.1 mg/mL BSA and 5% (v:v) glycerol, 100 nM purified protein, 5 mM ATP with/without 10 nM DNA fork. The DNA fork was prepared using the following oligonucleotides (D1: 5’-CTACTACCCCCACCCTCACAACCTTTTTTTTTTTTTT-3’; D2: 5’-TTTTTTTTTTTTTTGGTTGTGAGGGTGGGGGTA-3’; the complementary sequences are underlined). Samples were incubated at 37 °C for 30 min. After incubation, Malachite Green Reagent solution (volume: 200 μL) was added to each sample and incubation continued at room temperature for additional 10 min. A_635_ was measured using the Infinite F200 PRO TECAN instrument. Concentrations of inorganic phosphate were determined using a standard curve derived from known phosphate concentrations.

### Statistical analysis

Statistical significance (*P* values), analysis, and graphs were performed using GraphPad Prism 6 software. Statistical tests and differences are mentioned in the respective figure legend.

### Antibodies

The following commercial primary antibodies were used: anti-Flag peroxidase-conjugated mouse monoclonal antibody (Sigma-Aldrich cat. A8592); anti-AND-1 mouse monoclonal antibody (Novus cat. H00011169-M01); anti-FANCJ rabbit polyclonal antibody (for Western blot analysis; Novus cat. NBP1-31883); anti-Myc mouse monoclonal antibody (ab32, Abcam); anti-FANCJ rabbit polyclonal antibody (for SiRF analyses; Sigma-Aldrich cat. B1310); anti-poly-Histidine peroxidase-conjugated mouse monoclonal antibody (Sigma-Aldrich cat. A7058); anti-GST antibody (Cytiva cat. RPN1236); anti-β-tubulin mouse monoclonal antibody (Elabscience cat. E-AB-20033); anti-γ-H2AX rabbit monoclonal antibody (Abcam cat. ab81299); anti-biotin mouse monoclonal antibody (ThermoFischer Scientific cat. MA5-11251); anti-MCM4 rabbit polyclonal antibody (Abcam cat. Ab4459); anti-BrdU rat monoclonal antibody (Abcam cat. ab6326); anti-BrdU mouse monoclonal antibody (BD Biosciences cat. 347580); anti-BRCA1 rabbit polyclonal antibody (Proteintech cat. 22362-1-AP); anti-BLM mouse monoclonal antibody (Santa Cruz Biotechnology cat. sc-365753); anti-MLH1 rabbit monoclonal antibody (Bethyl laboratories cat. A700-112-T).

The following secondary antibodies were used: anti-mouse IgG peroxidase-conjugated goat polyclonal antibody (ThermoFisher Scientific cat. 31439); anti-rabbit IgG peroxidase-conjugated goat polyclonal antibody (ThermoFisher Scientific cat. 31462); anti-rabbit goat antibody labeled with Alexa Fluor™ 488 (ThermoFisher Scientific cat. A11008); anti-rat goat antibody labeled with Alexa Fluor™ 647 (ThermoFisher Scientific cat. A21247); anti-mouse polyclonal goat antibody labeled with Alexa Fluor™ 555 (ThermoFisher Scientific cat. A21422).

## Supplementary information

Source Data Fig. 1

Source Data Fig. 2

Source Data Fig. 3

Source Data Fig. 4

Source Data Fig. 5

Source Data Fig. 6

Source Data Fig. 7

Peer Review File

Expanded View Figures

## Data Availability

This study includes no data deposited in external repositories. All the materials described are available upon request.

## References

[CR1] Abe T, Kawasumi R, Giannattasio M, Dusi S, Yoshimoto Y, Miyata K, Umemura K, Hirota K, Branzei D (2018) AND-1 fork protection function prevents fork resection and is essential for proliferation. Nat Commun 9:3091 30082684 10.1038/s41467-018-05586-7 PMC6079002

[CR2] Alabert C, Bukowski-Wills JC, Lee SB, Kustatscher G, Nakamura K, De Lima Alves F, Menard P, Mejlvang J, Rappsilber J, Groth A (2014) Nascent chromatin capture proteomics determines chromatin dynamics during DNA replication and identifies unknown fork components. Nat Cell Biol 16:281–291 24561620 10.1038/ncb2918 PMC4283098

[CR3] Ali M, Delozier CD, Chaudhary U (2019) BRIP-1 germline mutation and its role in colon cancer: Presentation of two case reports and review of literature. BMC Med Genet 20:75 31064327 10.1186/s12881-019-0812-0 PMC6505212

[CR4] Barthelemy J, Hanenberg H, Leffak M (2016) FANCJ is essential to maintain microsatellite structure genome-wide during replication stress. Nucleic Acids Res 44:6803–6816 27179029 10.1093/nar/gkw433 PMC5001596

[CR5] Bharti SK, Sommers JA, George F, Kuper J, Hamon F, Shin-Ya K, Teulade-Fichou MP, Kisker C, Brosh RM (2013) Specialization among iron-sulfur cluster helicases to resolve G-quadruplex DNA structures that threaten genomic stability. J Biol Chem 288:28217–28229 23935105 10.1074/jbc.M113.496463 PMC3784731

[CR6] Borges V, Smith DJ, Whitehouse I, Uhlmann F (2013) An Eco1-independent sister chromatid cohesion establishment pathway in *S. cerevisiae*. Chromosoma 122:121–134 23334284 10.1007/s00412-013-0396-y PMC3608886

[CR7] Bridge WL, Vandenberg CJ, Franklin RJ, Hiom K (2005) The BRIP1 helicase functions independently of BRCA1 in the Fanconi anemia pathway for DNA crosslink repair. Nat Genet 37:953–957 16116421 10.1038/ng1627

[CR8] Brosh RM, Cantor SB (2014) Molecular and cellular functions of the FANCJ DNA helicase defective in cancer and in Fanconi anemia. Front Genet 5:372 25374583 10.3389/fgene.2014.00372 PMC4204437

[CR9] Brosh RM, Wu Y (2021) An emerging picture of FANCJ’s role in G4 resolution to facilitate DNA replication. NAR Cancer 3:zcab034 34873585 10.1093/narcan/zcab034 PMC8378518

[CR10] Calì F, Bharti SK, Di Perna R, Brosh RM, Pisani FM (2015) Tim/Timeless, a member of the replication fork protection complex, operates with the Warsaw breakage syndrome DNA helicase DDX11 in the same fork recovery pathway. Nucleic Acids Res 44:705–717 26503245 10.1093/nar/gkv1112 PMC4737141

[CR11] Calvo JA, Fritchman B, Hernandez D, Persky NS, Johannessen CM, Piccioni F, Kelch BA, Cantor SB (2021) Comprehensive mutational analysis of the BRCA1-associated DNA helicase and tumor-suppressor FANCJ/BACH1/BRIP1. Mol Cancer Res 19:1015–1025 33619228 10.1158/1541-7786.MCR-20-0828 PMC8178215

[CR12] Cantor SB, Bell DW, Ganesan S, Kass EM, Drapkin R, Grossman S, Wahrer DCR, Sgroi DC, Lane WS, Haber DA et al (2001) BACH1, a novel helicase-like protein, interacts directly with BRCA1 and contributes to its DNA repair function. Cell 105:149–160 11301010 10.1016/s0092-8674(01)00304-x

[CR13] Cantor SB, Guillemette S (2011) Hereditary breast cancer and the BRCA1-associated FANCJ/BACH1/BRIP1. *Futur*. Oncol 7:253–261 10.2217/fon.10.191 PMC3109611 21345144

[CR14] Carey MF, Peterson CL, Smale ST (2013) PCR-mediated site-directed mutagenesis. Cold Spring Harb Protoc 2013:738–742 23906918 10.1101/pdb.prot076505

[CR15] Castillo Bosch P, Segura‐Bayona S, Koole W, van Heteren JT, Dewar JM, Tijsterman M, Knipscheer P (2014) FANCJ promotes DNA synthesis through G‐quadruplex structures. EMBO J 33:2521–2533 25193968 10.15252/embj.201488663 PMC4282361

[CR16] Cheung I, Schertzer M, Rose A, Lansdorp PM (2002) Disruption of dog-1 in *Caenorhabditis elegans* triggers deletions upstream of guanine-rich DNA. Nat Genet 31:405–409 12101400 10.1038/ng928

[CR17] Cortone G, Zheng G, Pensieri P, Chiappetta V, Tatè R, Malacaria E, Pichierri P, Yu H, Pisani FM (2018) Interaction of the Warsaw breakage syndrome DNA helicase DDX11 with the replication fork-protection factor Timeless promotes sister chromatid cohesion. PLoS Genet 14:e1007622 30303954 10.1371/journal.pgen.1007622 PMC6179184

[CR18] Easton DF, Lesueur F, Decker B, Michailidou K, Li J, Allen J, Luccarini C, Pooley KA, Shah M, Bolla MK et al (2016) No evidence that protein truncating variants in BRIP1 are associated with breast cancer risk: Implications for gene panel testing. J Med Genet 53:298–309 26921362 10.1136/jmedgenet-2015-103529 PMC4938802

[CR19] Farina A, Shin JH, Kim DH, Bermudez VP, Kelman Z, Seo YS, Hurwitz J (2008) Studies with the human cohesin establishment factor, ChlR1: association of ChlR1 with Ctf18-RFC and Fen1. J Biol Chem 283:20925–20936 18499658 10.1074/jbc.M802696200 PMC2475708

[CR20] Fumasoni M, Zwicky K, Vanoli F, Lopes M, Branzei D (2015) Error-Free DNA damage tolerance and sister chromatid proximity during DNA replication rely on the Polα/Primase/Ctf4 complex. Mol Cell 57:812–823 25661486 10.1016/j.molcel.2014.12.038 PMC4352764

[CR21] Gambus A, Van Deursen F, Polychronopoulos D, Foltman M, Jones RC, Edmondson RD, Calzada A, Labib K (2009) A key role for Ctf4 in coupling the MCM2-7 helicase to DNA polymerase α within the eukaryotic replisome. EMBO J 28:2992–3004 19661920 10.1038/emboj.2009.226 PMC2760104

[CR22] Gong Z, Kim JE, Leung CCY, Glover JNM, Chen J (2010) BACH1/FANCJ acts with TopBP1 and participates early in DNA replication checkpoint control. Mol Cell 37:438–446 20159562 10.1016/j.molcel.2010.01.002 PMC3695484

[CR23] Gosnell JA, Christensen TW (2011) Drosophila Ctf4 is essential for efficient DNA replication and normal cell cycle progression. BMC Mol Biol 12:13 21470422 10.1186/1471-2199-12-13 PMC3082215

[CR24] Guan C, Li J, Sun D, Liu Y, Liang H (2017) The structure and polymerase-recognition mechanism of the crucial adaptor protein AND-1 in the human replisome. J Biol Chem 292:9627–9636 28381552 10.1074/jbc.M116.758524 PMC5465487

[CR25] Guillou E, Ibarra A, Coulon V, Casado-Vela J, Rico D, Casal I, Schwob E, Losada A, Méndez J (2010) Cohesin organizes chromatin loops at DNA replication factories. Genes Dev 24:2812–2822 21159821 10.1101/gad.608210 PMC3003199

[CR26] Gupta R, Sharma S, Sommers JA, Jin Z, Cantor SB, Brosh RM (2005) Analysis of the DNA substrate specificity of the human BACH1 helicase associated with breast cancer. J Biol Chem 280:25450–25460 15878853 10.1074/jbc.M501995200

[CR27] Henderson A, Wu Y, Huang YC, Chavez EA, Platt J, Johnson FB, Brosh RM, Sen D, Lansdorp PM (2014) Detection of G-quadruplex DNA in mammalian cells. Nucleic Acids Res 42:860–869 24163102 10.1093/nar/gkt957 PMC3902944

[CR28] Jones ML, Baris Y, Taylor MRG, Yeeles JTP (2021) Structure of a human replisome shows the organisation and interactions of a DNA replication machine. EMBO J 40:e108819 34694004 10.15252/embj.2021108819 PMC8634136

[CR29] Jumper J, Evans R, Pritzel A, Green T, Figurnov M, Ronneberger O, Tunyasuvunakool K, Bates R, Žídek A, Potapenko A et al (2021) Highly accurate protein structure prediction with AlphaFold. Nature 596:583–589 34265844 10.1038/s41586-021-03819-2 PMC8371605

[CR30] Kilkenny ML, Simon AC, Mainwaring J, Wirthensohn D, Holzer S, Pellegrini L (2017) The human CTF4-orthologue AND-1 interacts with DNA polymerase a/primase via its unique C-Terminal HMG box. Open Biol 7:170217 29167311 10.1098/rsob.170217 PMC5717350

[CR31] Lee WTC, Yin Y, Morten MJ, Tonzi P, Gwo PP, Odermatt DC, Modesti M, Cantor SB, Gari K, Huang TT et al (2021) Single-molecule imaging reveals replication fork coupled formation of G-quadruplex structures hinders local replication stress signaling. Nat Commun 12:2525 33953191 10.1038/s41467-021-22830-9 PMC8099879

[CR32] Lerner LK, Holzer S, Kilkenny ML, Šviković S, Murat P, Schiavone D, Eldridge CB, Bittleston A, Maman JD, Branzei D et al (2020) Timeless couples G‐quadruplex detection with processing by DDX 11 helicase during DNA replication. EMBO J 39:e104185 32705708 10.15252/embj.2019104185 PMC7506991

[CR33] Levitus M, Waisfisz Q, Godthelp BC, De Vries Y, Hussain S, Wiegant WW, Elghalbzouri-Maghrani E, Steltenpool J, Rooimans MA, Pals G et al (2005) The DNA helicase BRIP1 is defective in Fanconi anemia complementation group J. Nat Genet 37:934–935 16116423 10.1038/ng1625

[CR34] Levran O, Attwooll C, Henry RT, Milton KL, Neveling K, Rio P, Batish SD, Kalb R, Velleuer E, Barral S et al (2005) The BRCA1-interacting helicase BRIP1 is deficient in Fanconi anemia. Nat Genet 37:931–933 16116424 10.1038/ng1624

[CR35] Litman R, Peng M, Jin Z, Zhang F, Zhang J, Powell S, Andreassen PR, Cantor SB (2005) BACH1 is critical for homologous recombination and appears to be the Fanconi anemia gene product FANCJ. Cancer Cell 8:255–265 16153896 10.1016/j.ccr.2005.08.004

[CR36] London TBC, Barber LJ, Mosedale G, Kelly GP, Balasubramanian S, Hickson ID, Boulton SJ, Hiom K (2008) FANCJ is a structure-specific DNA helicase associated with the maintenance of genomic G/C tracts. J Biol Chem 283:36132–36139 18978354 10.1074/jbc.M808152200 PMC2662291

[CR37] Lowran K, Campbell L, Popp P, Wu CG (2020) Assembly of a G-Quadruplex repair complex by the FANCJ DNA helicase and the REV1 polymerase. Genes 11:5 10.3390/genes11010005 PMC7017153 31861576

[CR38] Masai H, Matsumoto S, You Z, Yoshizawa-Sugata N, Oda M (2010) Eukaryotic chromosome DNA replication: Where, when, and how? Annu Rev Biochem 79:89–130 20373915 10.1146/annurev.biochem.052308.103205

[CR39] Matsuzaki K, Borel V, Adelman CA, Schindler D, Boulton SJ (2015) FANCJ suppresses microsatellite instability and lymphomagenesis independent of the Fanconi anemia pathway. Genes Dev 29:2532–2546 26637282 10.1101/gad.272740.115 PMC4699383

[CR40] Moyer CL, Ivanovich J, Gillespie JL, Doberstein R, Radke MR, Richardson ME, Kaufmann SH, Swisher EM, Goodfellow PJ (2020) Rare BRIP1 missense alleles confer risk for ovarian and breast cancer. Cancer Res 80:857–867 31822495 10.1158/0008-5472.CAN-19-1991 PMC8722358

[CR41] Nath S, Nagaraju G (2020) FANCJ helicase promotes DNA end resection by facilitating CtIP recruitment to DNA double-strand breaks. PLoS Genet 16:e1008701 32251466 10.1371/journal.pgen.1008701 PMC7162537

[CR42] Nieminuszczy J, Schwab RA, Niedzwiedz W (2016) The DNA fibre technique-tracking helicases at work. Methods 108:92–98 27102626 10.1016/j.ymeth.2016.04.019

[CR43] Norquist BM, Harrell MI, Brady MF, Walsh T, Lee MK, Gulsuner S, Bernards SS, Casadei S, Yi Q, Burger RA et al (2016) Inherited mutations in women with ovarian carcinoma. JAMA Oncol 2:482–490 26720728 10.1001/jamaoncol.2015.5495 PMC4845939

[CR44] Odermatt DC, Lee WTC, Wild S, Jozwiakowski SK, Rothenberg E, Gari K (2020) Cancer-associated mutations in the iron-sulfur domain of FANCJ affect G-quadruplex metabolism. PLoS Genet 16:e1008740 32542039 10.1371/journal.pgen.1008740 PMC7316351

[CR45] Okumura M, Natsume T, Kanemaki MT, Kiyomitsu T (2018) Dynein–dynactin–NuMA clusters generate cortical spindle-pulling forces as a multiarm ensemble. Elife 7:e36559 29848445 10.7554/eLife.36559 PMC6037482

[CR46] Paulo P, Maia S, Pinto C, Pinto P, Monteiro A, Peixoto A, Teixeira MR (2018) Targeted next generation sequencing identifies functionally deleterious germline mutations in novel genes in early-onset/familial prostate cancer. PLoS Genet 14:e1007355 29659569 10.1371/journal.pgen.1007355 PMC5919682

[CR47] Peng M, Cong K, Panzarino NJ, Nayak S, Calvo J, Deng B, Zhu LJ, Morocz M, Hegedus L, Haracska L et al (2018) Opposing roles of FANCJ and HLTF protect forks and restrain replication during stress. Cell Rep 24:3251–3261 30232006 10.1016/j.celrep.2018.08.065 PMC6218949

[CR48] Peng M, Litman R, Xie J, Sharma S, Brosh RM, Cantor SB (2007) The FANCJ/MutLα interaction is required for correction of the cross-link response in FA-J cells. EMBO J 26:3238–3249 17581638 10.1038/sj.emboj.7601754 PMC1914102

[CR49] Quinet A, Carvajal-Maldonado D, Lemacon D, Vindigni A (2017) DNA fiber analysis: mind the gap! Methods Enzymol 591:55–82 28645379 10.1016/bs.mie.2017.03.019

[CR50] Ramus SJ, Song H, Dicks E, Tyrer JP, Rosenthal AN, Intermaggio MP, Fraser L, Gentry-Maharaj A, Hayward J, Philpott S et al (2015) Germline mutations in the BRIP1, BARD1, PALB2, and NBN genes in women with ovarian cancer. J Natl Cancer Inst 107:djv214 26315354 10.1093/jnci/djv214 PMC4643629

[CR51] Roy S, Luzwick JW, Schlacher K (2018) SIRF: quantitative in situ analysis of protein interactions at DNA replication forks. J Cell Biol 217:1553–1553 29475976 10.1083/jcb.201709121 PMC5881507

[CR52] Rzechorzek NJ, Hardwick SW, Jatikusumo VA, Chirgadze DY, Pellegrini L (2020) CryoEM structures of human CMG-ATPγS-DNA and CMG-AND-1 complexes. Nucleic Acids Res 48:6980–6995 32453425 10.1093/nar/gkaa429 PMC7337937

[CR53] Samora CP, Saksouk J, Goswami P, Wade BO, Singleton MR, Bates PA, Lengronne A, Costa A, Uhlmann F (2016) Ctf4 links DNA replication with sister chromatid cohesion establishment by recruiting the Chl1 helicase to the replisome. Mol Cell 63:371–384 27397686 10.1016/j.molcel.2016.05.036 PMC4980427

[CR54] Sarkies P, Murat P, Phillips LG, Patel KJ, Balasubramanian S, Sale JE (2012) FANCJ coordinates two pathways that maintain epigenetic stability at G-quadruplex DNA. Nucleic Acids Res 40:1485–1498 22021381 10.1093/nar/gkr868 PMC3287192

[CR55] Sasaki M, Kobayashi T (2017) Ctf4 prevents genome rearrangements by suppressing DNA double-strand break formation and its end resection at arrested replication forks. Mol Cell 66:533–545.e5 28525744 10.1016/j.molcel.2017.04.020

[CR56] Sato K, Martin-Pintado N, Post H, Altelaar M, Knipscheer P (2021) Multistep mechanism of G-quadruplex resolution during DNA replication. Sci Adv 7:8653–8677 10.1126/sciadv.abf8653 PMC8462899 34559566

[CR57] Saxena S, Zou L (2022) Hallmarks of DNA replication stress. Mol Cell 82:2298–2314 35714587 10.1016/j.molcel.2022.05.004 PMC9219557

[CR58] Van Schendel R, Romeijn R, Buijs H, Tijsterman M (2021) Preservation of lagging strand integrity at sites of stalled replication by Pol α-primase and 9-1-1 complex. Sci Adv 7:eabf2278 34138739 10.1126/sciadv.abf2278 PMC8133754

[CR59] Schwab RA, Nieminuszczy J, Shin-ya K, Niedzwiedz W (2013) FANCJ couples replication past natural fork barriers with maintenance of chromatin structure. J Cell Biol 201:33–48 23530069 10.1083/jcb.201208009 PMC3613694

[CR60] Simon AC, Zhou JC, Perera RL, Van Deursen F, Evrin C, Ivanova ME, Kilkenny ML, Renault L, Kjaer S, Matak-Vinkovi D et al (2014) A Ctf4 trimer couples the CMG helicase to DNA polymerase α in the eukaryotic replisome. Nature 510:293–297 24805245 10.1038/nature13234 PMC4059944

[CR61] Sommers JA, Banerjee T, Hinds T, Wan B, Wold MS, Lei M, Brosh RM (2014) Novel function of the Fanconi anemia group J or RECQ1 helicase to disrupt protein-DNA complexes in a replication protein A-stimulated manner. J Biol Chem 289:19928–19941 24895130 10.1074/jbc.M113.542456 PMC4106313

[CR62] Spencer F, Gerring SL, Connelly C, Hieter P (1990) Mitotic chromosome transmission fidelity mutants in Saccharomyces cerevisiae. Genetics 124:237–249 2407610 10.1093/genetics/124.2.237 PMC1203917

[CR63] Suhasini AN, Rawtani NA, Wu Y, Sommers JA, Sharma S, Mosedale G, North PS, Cantor SB, Hickson ID, Brosh RM (2011) Interaction between the helicases genetically linked to Fanconi anemia group J and Bloom’s syndrome. EMBO J 30:692–705 21240188 10.1038/emboj.2010.362 PMC3041957

[CR64] Suhasini AN, Sommers JA, Muniandy PA, Coulombe Y, Cantor SB, Masson J-Y, Seidman MM, Brosh RM (2013) Fanconi anemia group J helicase and MRE11 nuclease interact to facilitate the DNA damage response. Mol Cell Biol 33:2212–2227 23530059 10.1128/MCB.01256-12 PMC3648079

[CR65] Summers PA, Lewis BW, Gonzalez-Garcia J, Porreca RM, Lim AHM, Cadinu P, Martin-Pintado N, Mann DJ, Edel JB, Vannier JB et al (2021) Visualising G-quadruplex DNA dynamics in live cells by fluorescence lifetime imaging microscopy. Nat Commun 12:162 33420085 10.1038/s41467-020-20414-7 PMC7794231

[CR66] Tanaka H, Katou Y, Yagura M, Saitoh K, Itoh T, Araki H, Bando M, Shirahige K (2009) Ctf4 coordinates the progression of helicase and DNA polymerase α. Genes to Cells 14:807–820 19496828 10.1111/j.1365-2443.2009.01310.x

[CR67] Uno S, You Z, Masai H (2012) Purification of replication factors using insect and mammalian cell expression systems. Methods 57:214–221 22800621 10.1016/j.ymeth.2012.06.016

[CR68] Villa F, Simon AC, Ortiz Bazan MA, Kilkenny ML, Wirthensohn D, Wightman M, Matak-Vinkovíc D, Pellegrini L, Labib K (2016) Ctf4 is a hub in the eukaryotic replisome that links multiple CIP-box proteins to the CMG helicase. Mol Cell 63:385–396 27397685 10.1016/j.molcel.2016.06.009 PMC4980431

[CR69] Weber-Lassalle N, Hauke J, Ramser J, Richters L, Groß E, Blümcke B, Gehrig A, Kahlert AK, Müller CR, Hackmann K et al (2018) BRIP1 loss-of-function mutations confer high risk for familial ovarian cancer, but not familial breast cancer. Breast Cancer Res 20:7 29368626 10.1186/s13058-018-0935-9 PMC5784717

[CR70] Williams DR, McIntosh JR (2005) Mcl1p is a polymerase α replication accessory factor important for S-phase DNA damage survival. Eukaryot Cell 4:166–177 15643072 10.1128/EC.4.1.166-177.2005 PMC544150

[CR71] Wu Y, Brosh Jr. R (2009) FANCJ helicase operates in the Fanconi anemia DNA repair pathway and the response to replicational stress. Curr Mol Med 9:470–482 19519404 10.2174/156652409788167159 PMC2763586

[CR72] Wu Y, Shin-ya K, Brosh RM (2008) FANCJ helicase defective in Fanconia anemia and breast cancer unwinds G-Quadruplex DNA to defend genomic stability. Mol Cell Biol 28:4116–4128 18426915 10.1128/MCB.02210-07 PMC2423121

[CR73] Yaneva D, Sparks JL, Donsbach M, Zhao S, Weickert P, Bezalel-Buch R, Stingele J, Walter JC (2023) The FANCJ helicase unfolds DNA-protein crosslinks to promote their repair. Mol Cell 83:43–56.e10 36608669 10.1016/j.molcel.2022.12.005 PMC9881729

[CR74] Yoshizawa-Sugata N, Masai H (2009) Roles of human AND-1 in chromosome transactions in S phase. J Biol Chem 284:20718–20728 19439411 10.1074/jbc.M806711200 PMC2742837

[CR75] Zhang Y, Li J, Zhou Y, Li Z, Peng C, Pei H, Zhu W (2022) AND-1 coordinates with the FANCM complex to regulate Fanconi anemia signaling and cisplatin resistance. Cancer Res 82:3249–3262 35867033 10.1158/0008-5472.CAN-22-0769 PMC9481708

