# Supplementary material for: FANCJ DNA helicase is recruited to the replisome by AND-1 to ensure genome stability
Source: EMBO Rep. 2024 Jan 2;25(2):24. doi: 10.1038/s44319-023-00044-y (PMC10897178; doi:10.1038/s44319-023-00044-y)
Supplement: Supplementary file 1 — Source Data Fig. 1 [file 44319_2023_44_MOESM1_ESM.zip › Source_Data_Figure_1/Panel_C/Figure_1_Panel C_WB.pptx]

## Slide 1
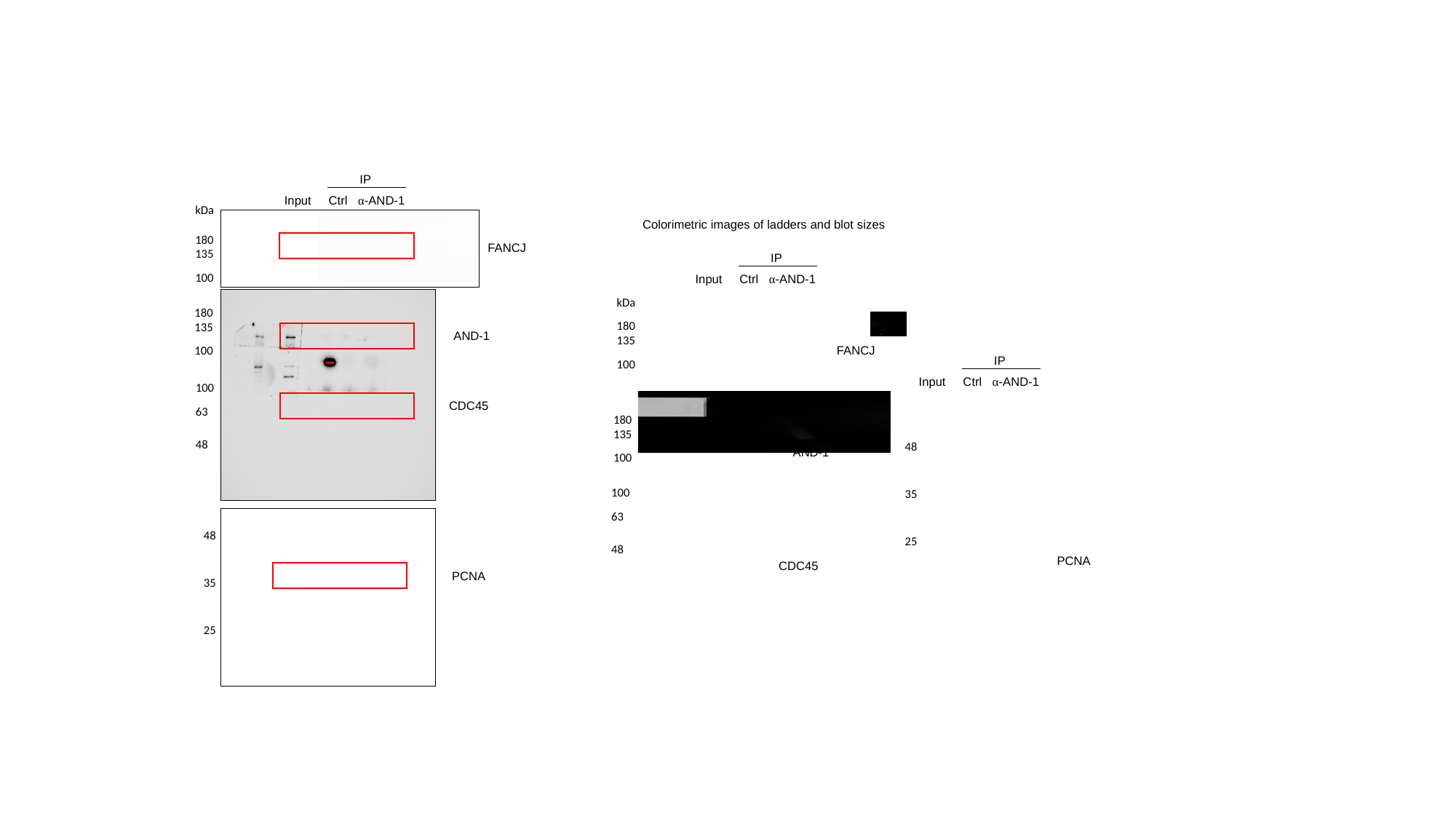

IP
Input Ctrl α-AND-1
kDa
Colorimetric images of ladders and blot sizes
180
135
100
FANCJ
IP
Input Ctrl α-AND-1
180
135
100
FANCJ
180
135
100
48
35
25
AND-1
100
63
48
PCNA
CDC45
kDa
180
135
100
AND-1
IP
Input Ctrl α-AND-1
100
63
48
CDC45
48
35
25
PCNA
