# Supplementary material for: FANCJ DNA helicase is recruited to the replisome by AND-1 to ensure genome stability
Source: EMBO Rep. 2024 Jan 2;25(2):24. doi: 10.1038/s44319-023-00044-y (PMC10897178; doi:10.1038/s44319-023-00044-y)
Supplement: Supplementary file 1 — Source Data Fig. 1 [file 44319_2023_44_MOESM1_ESM.zip › Source_Data_Figure_1/Panel_D/Figure 1 _Panel D - WB.pptx]

## Slide 1
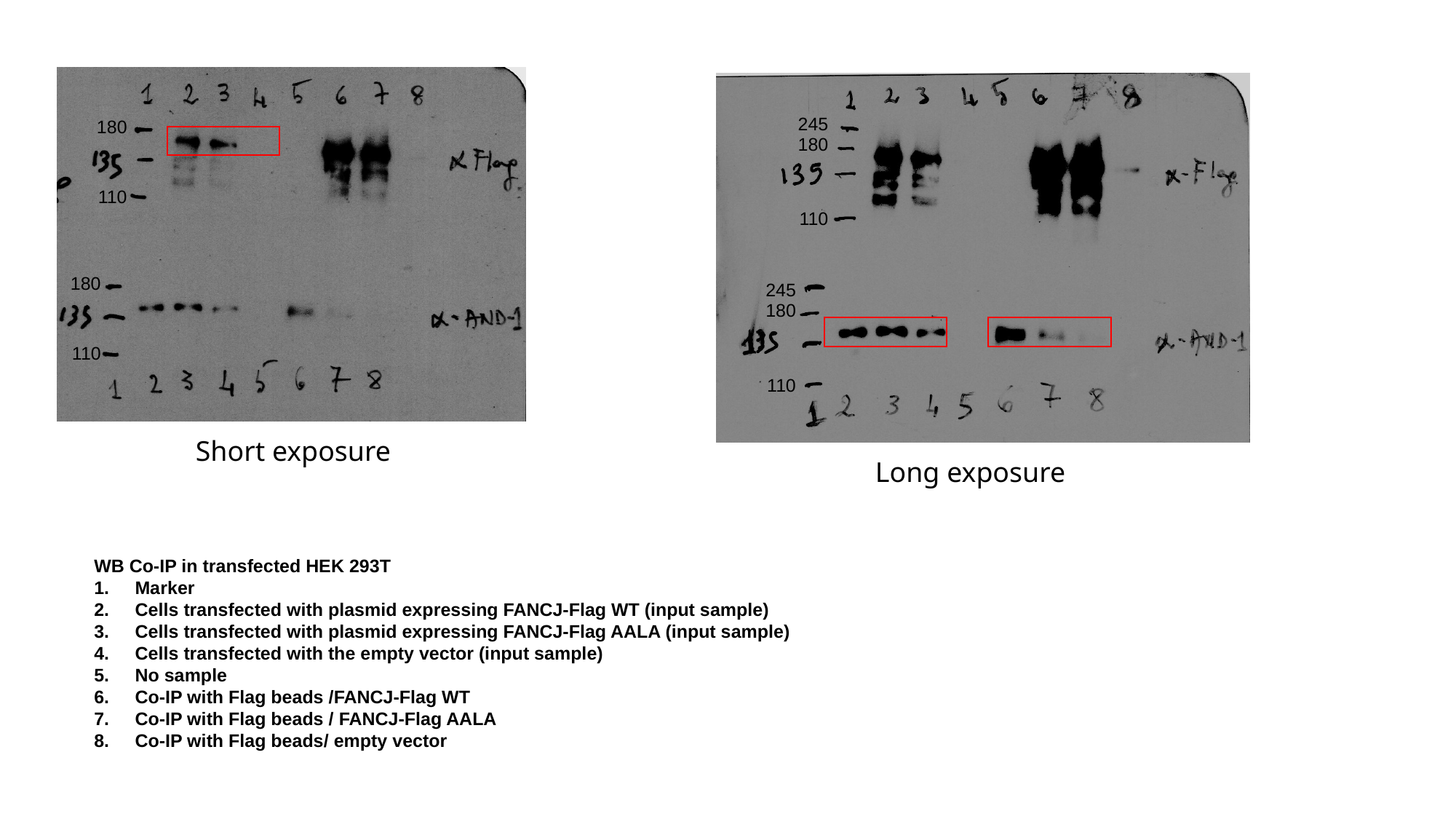

180
110
180
110
Short exposure
245
180
110
245
180
110
Long exposure
WB Co-IP in transfected HEK 293T
Marker
Cells transfected with plasmid expressing FANCJ-Flag WT (input sample)
Cells transfected with plasmid expressing FANCJ-Flag AALA (input sample)
Cells transfected with the empty vector (input sample)
No sample
Co-IP with Flag beads /FANCJ-Flag WT
Co-IP with Flag beads / FANCJ-Flag AALA
Co-IP with Flag beads/ empty vector
