# Supplementary material for: FANCJ DNA helicase is recruited to the replisome by AND-1 to ensure genome stability
Source: EMBO Rep. 2024 Jan 2;25(2):24. doi: 10.1038/s44319-023-00044-y (PMC10897178; doi:10.1038/s44319-023-00044-y)
Supplement: Supplementary file 2 — Source Data Fig. 2 [file 44319_2023_44_MOESM2_ESM.zip › Source_Data_Figure_2/Panel_D/Figure2_Panel D.pptx]

## Slide 1
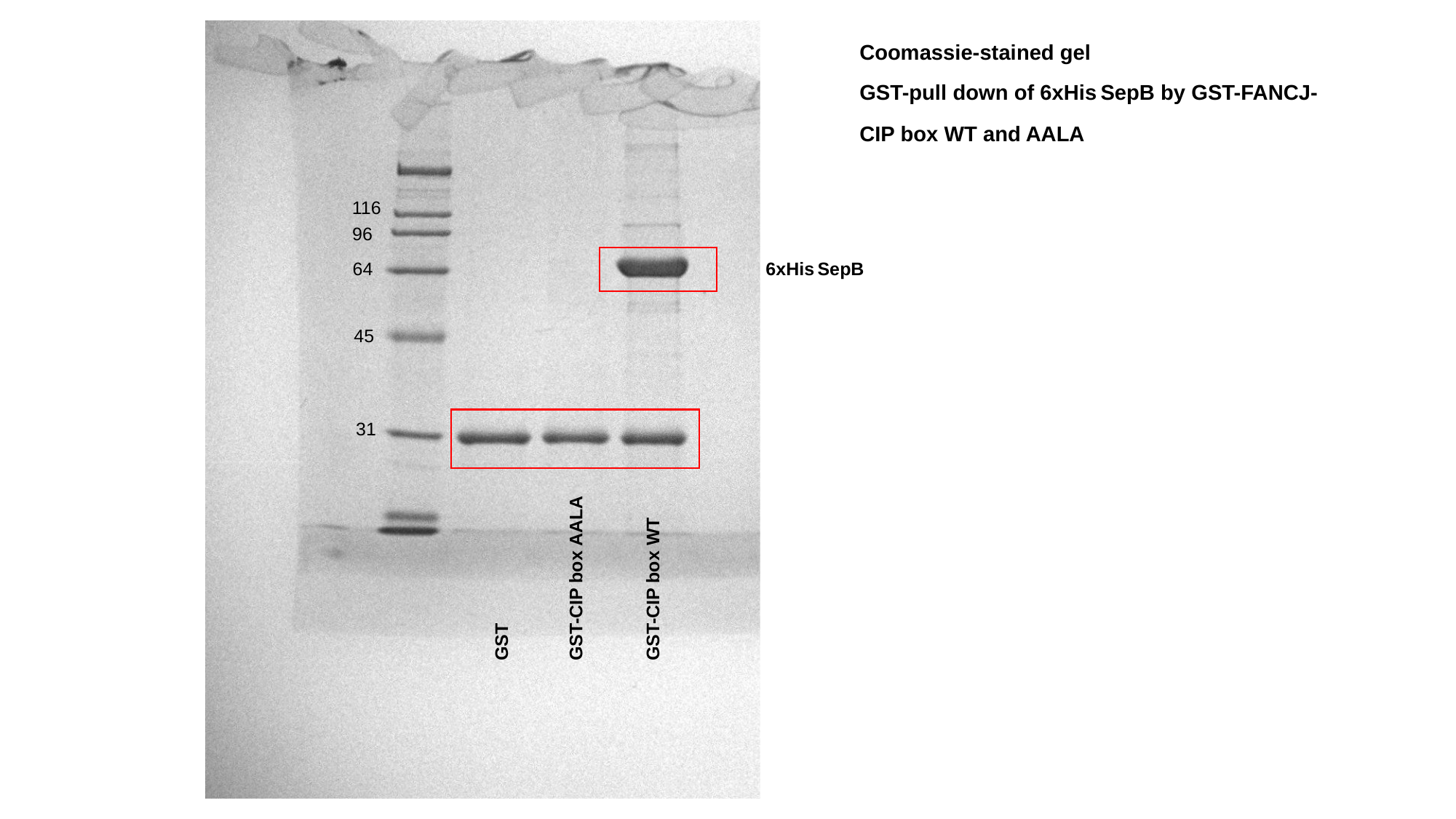

Coomassie-stained gel
GST-pull down of 6xHis SepB by GST-FANCJ-CIP box WT and AALA
116
96
64
45
31
6xHis SepB
GST-CIP box AALA
GST-CIP box WT
GST
