# Supplementary material for: FANCJ DNA helicase is recruited to the replisome by AND-1 to ensure genome stability
Source: EMBO Rep. 2024 Jan 2;25(2):24. doi: 10.1038/s44319-023-00044-y (PMC10897178; doi:10.1038/s44319-023-00044-y)
Supplement: Supplementary file 2 — Source Data Fig. 2 [file 44319_2023_44_MOESM2_ESM.zip › Source_Data_Figure_2/Panel_B/Figure 2 _Panel B.pptx]

## Slide 1
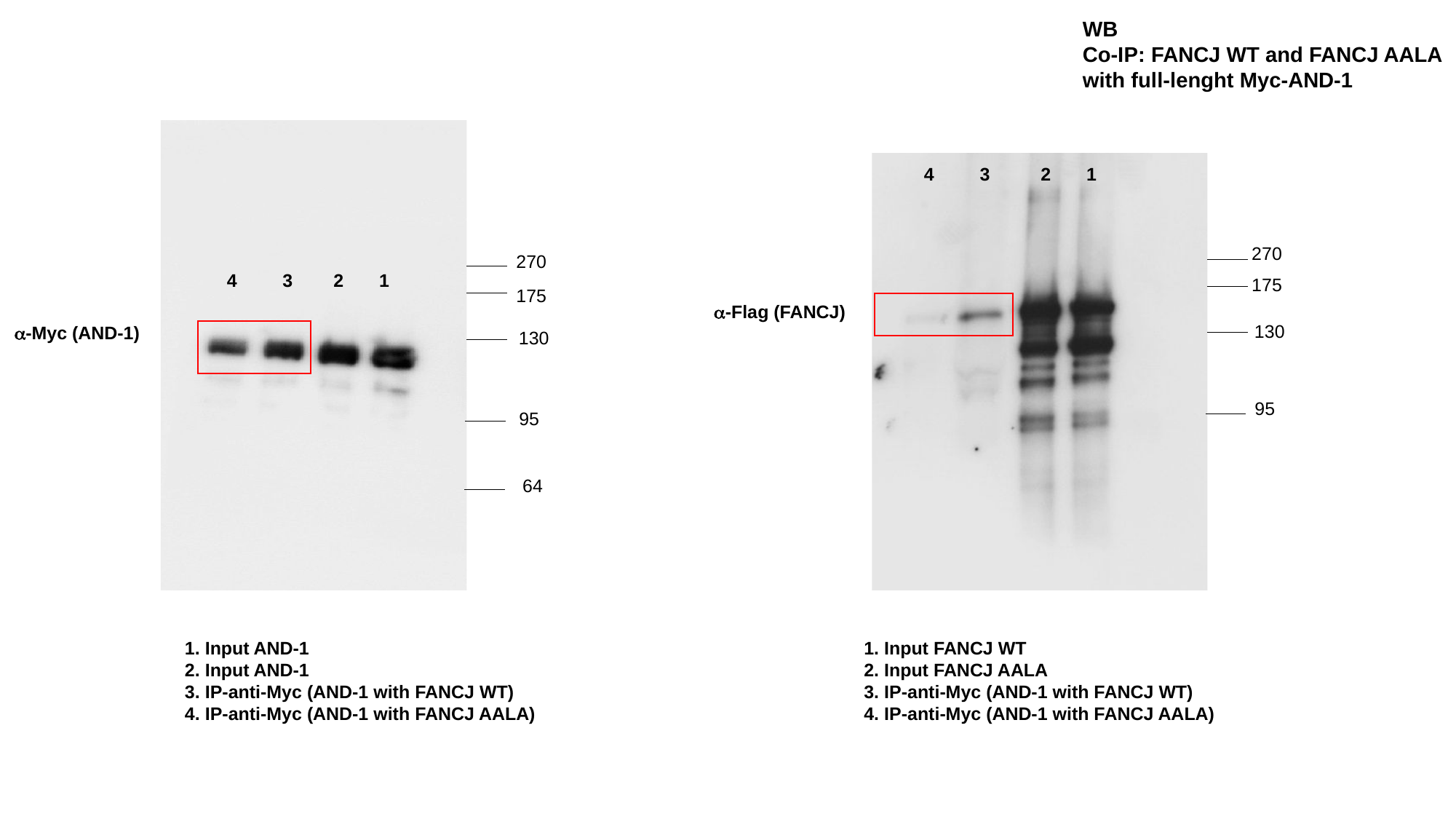

WB
Co-IP: FANCJ WT and FANCJ AALA with full-lenght Myc-AND-1
270
175
130
95
 4 3 2 1
a-Myc (AND-1)
64
 4 3 2 1
270
175
130
95
 a-Flag (FANCJ)
1. Input AND-1
2. Input AND-1
3. IP-anti-Myc (AND-1 with FANCJ WT)
4. IP-anti-Myc (AND-1 with FANCJ AALA)
1. Input FANCJ WT
2. Input FANCJ AALA
3. IP-anti-Myc (AND-1 with FANCJ WT)
4. IP-anti-Myc (AND-1 with FANCJ AALA)
