# Supplementary material for: FANCJ DNA helicase is recruited to the replisome by AND-1 to ensure genome stability
Source: EMBO Rep. 2024 Jan 2;25(2):24. doi: 10.1038/s44319-023-00044-y (PMC10897178; doi:10.1038/s44319-023-00044-y)
Supplement: Supplementary file 2 — Source Data Fig. 2 [file 44319_2023_44_MOESM2_ESM.zip › Source_Data_Figure_2/Panel_C /Figure 2_Panel C.pptx]

## Slide 1
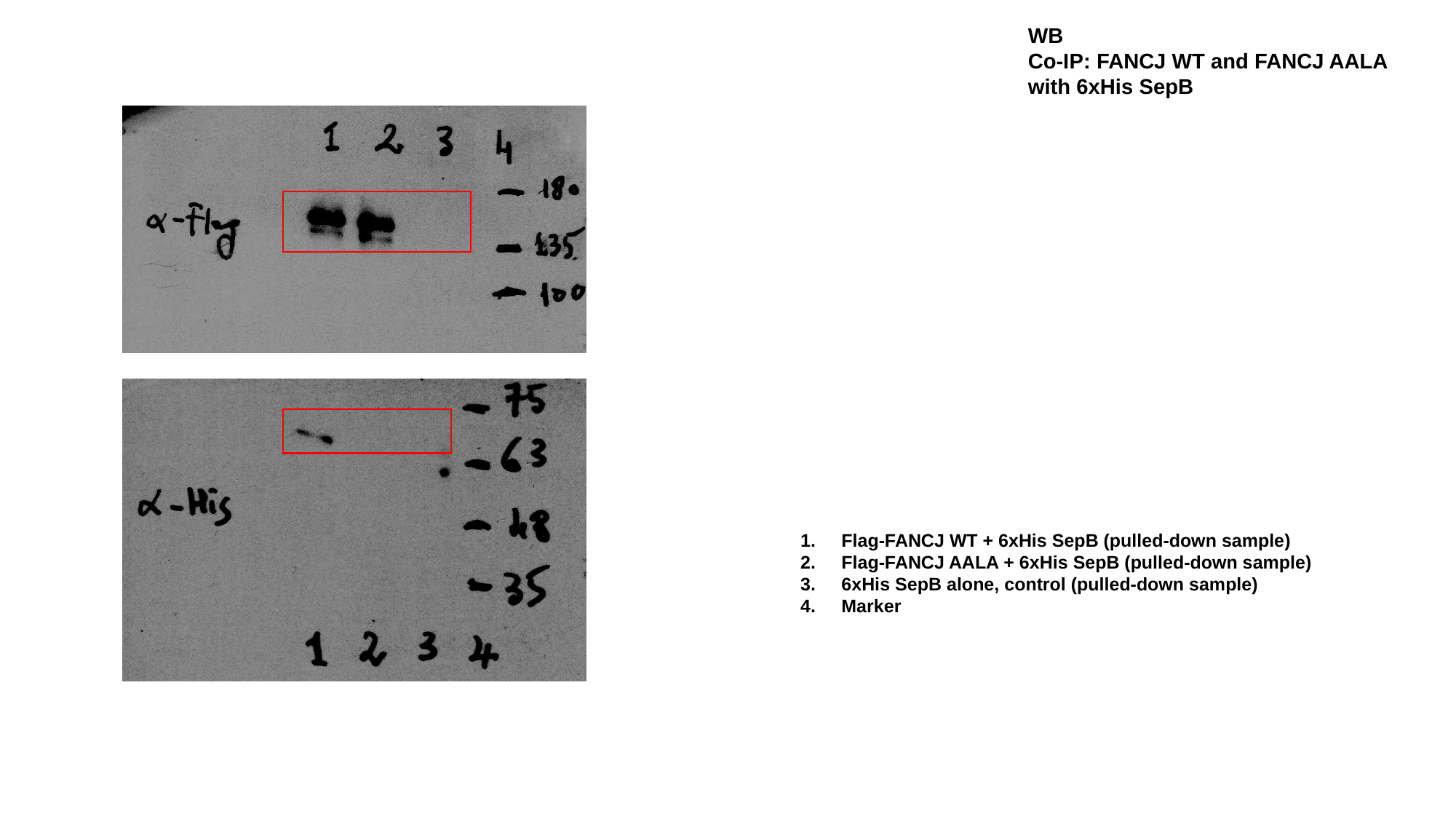

WB
Co-IP: FANCJ WT and FANCJ AALA with 6xHis SepB
Flag-FANCJ WT + 6xHis SepB (pulled-down sample)
Flag-FANCJ AALA + 6xHis SepB (pulled-down sample)
6xHis SepB alone, control (pulled-down sample)
Marker
