# Supplementary figures and images for: FANCJ DNA helicase is recruited to the replisome by AND-1 to ensure genome stability
Source: EMBO Rep. 2024 Jan 2;25(2):24. doi: 10.1038/s44319-023-00044-y (PMC10897178; doi:10.1038/s44319-023-00044-y)

## Slide 1
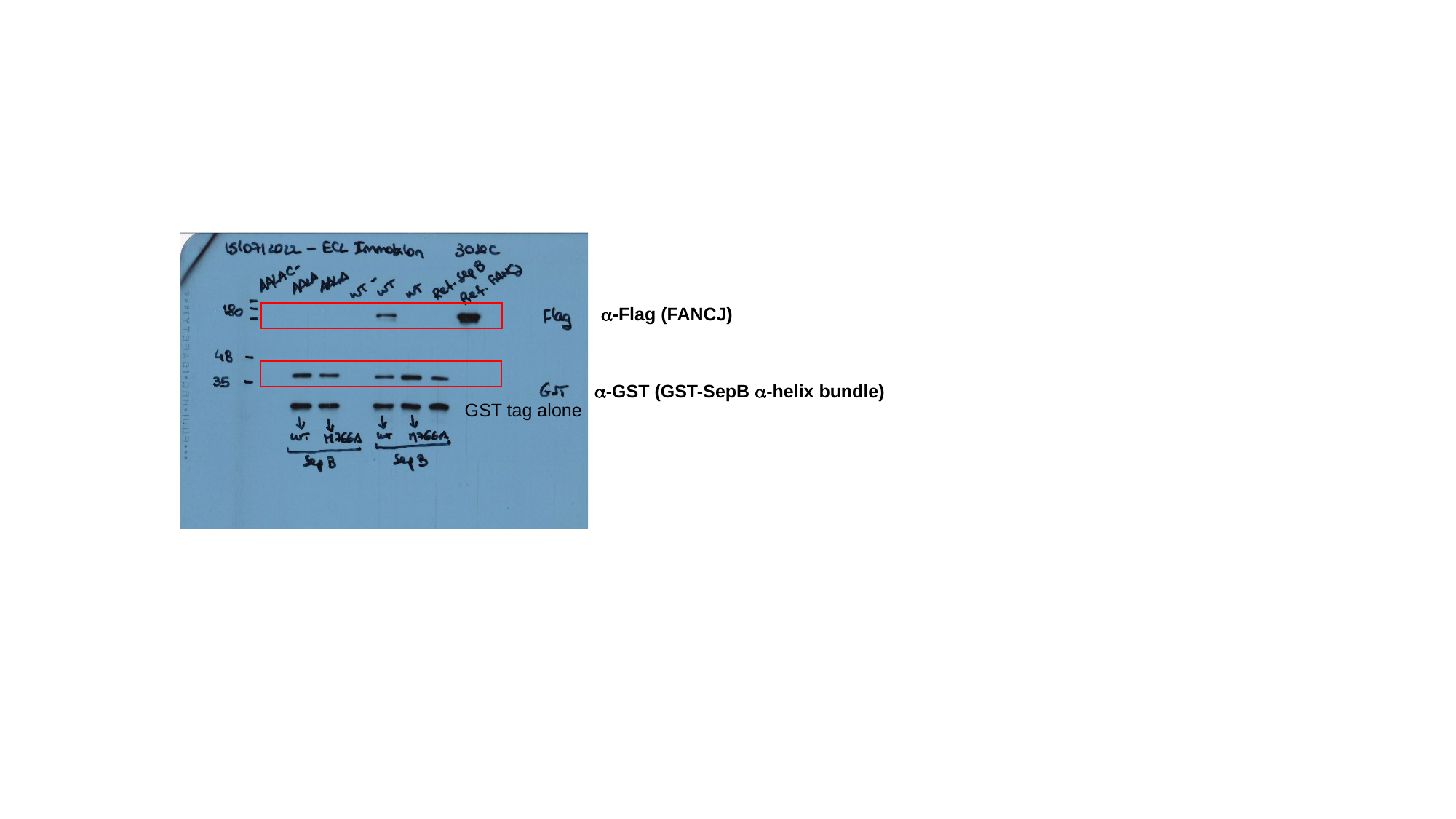

a-Flag (FANCJ)
a-GST (GST-SepB α-helix bundle)
GST tag alone

Supplement: Supplementary file 2 — Source Data Fig. 2 [file 44319_2023_44_MOESM2_ESM.zip › Source_Data_Figure_2/Panel_E/Figure_2_Panel E.pptx]

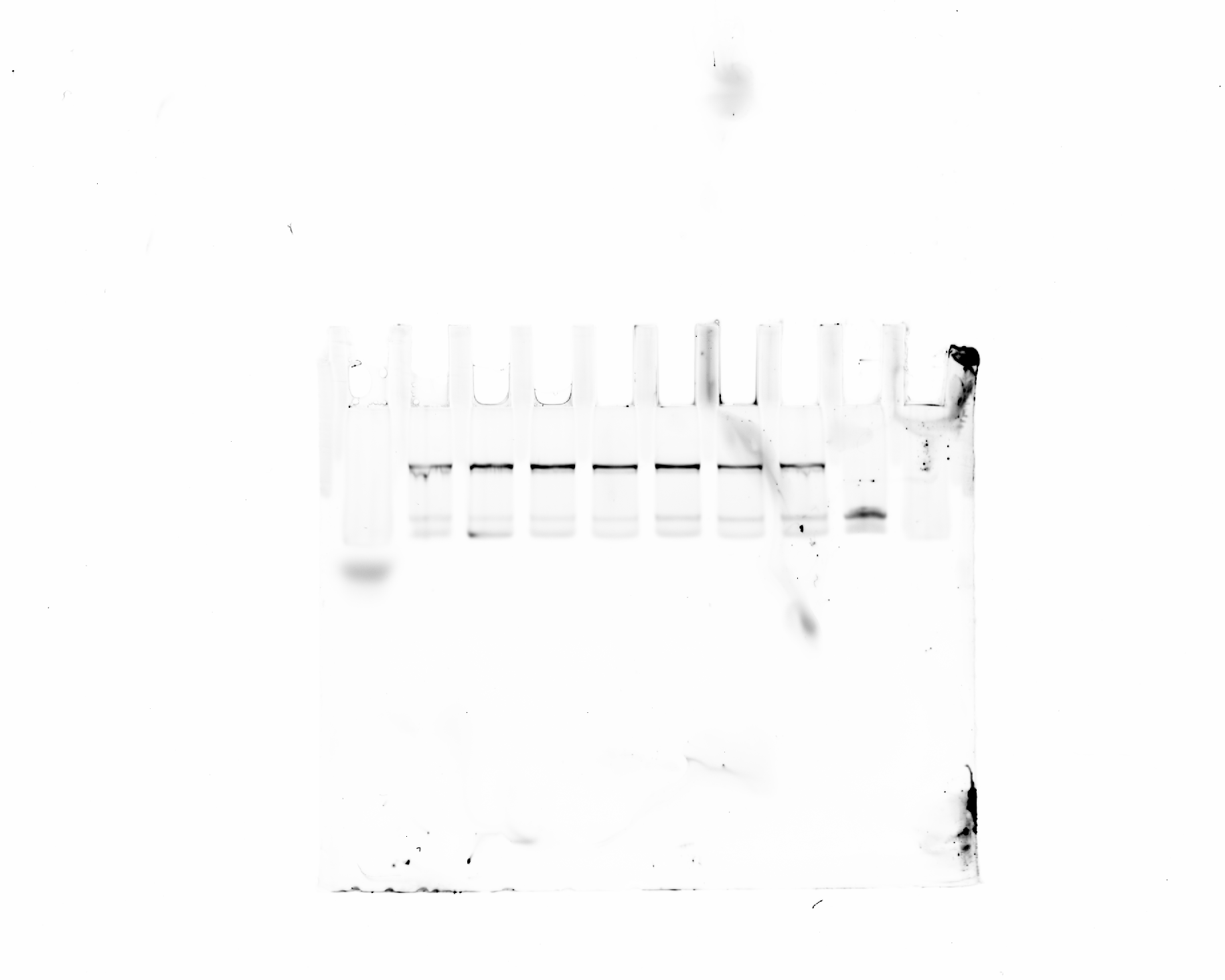

Supplement: Supplementary file 7 — Source Data Fig. 7 [file 44319_2023_44_MOESM7_ESM.zip › Source_Data_Figure_7/Panel_B/Figure_7_Panel_B_Helicase_Assay_FANCJ_K52R.tif]

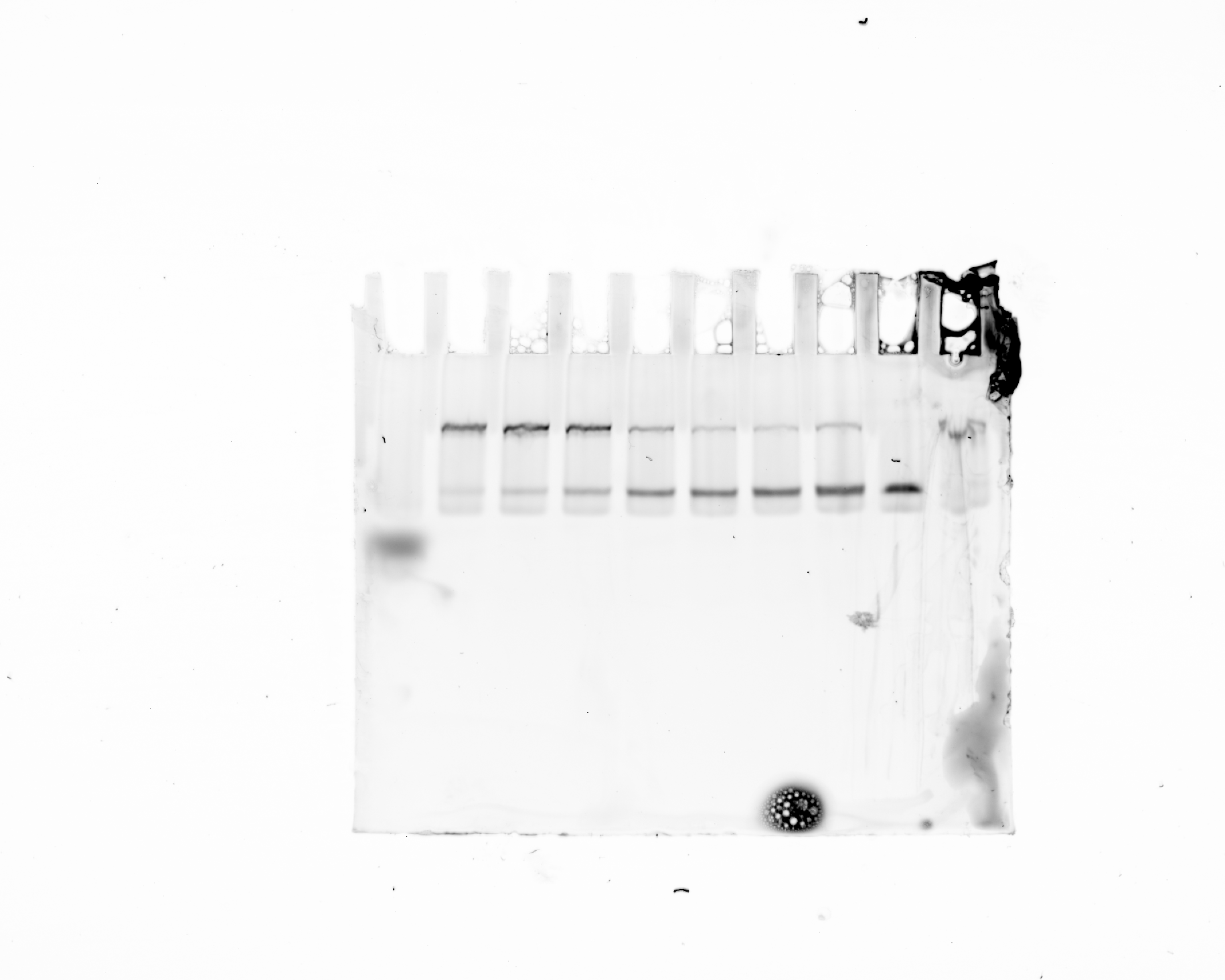

Supplement: Supplementary file 7 — Source Data Fig. 7 [file 44319_2023_44_MOESM7_ESM.zip › Source_Data_Figure_7/Panel_B/Figure_7_Panel_B_Helicase_Assay_FANCJ D736H.tif]

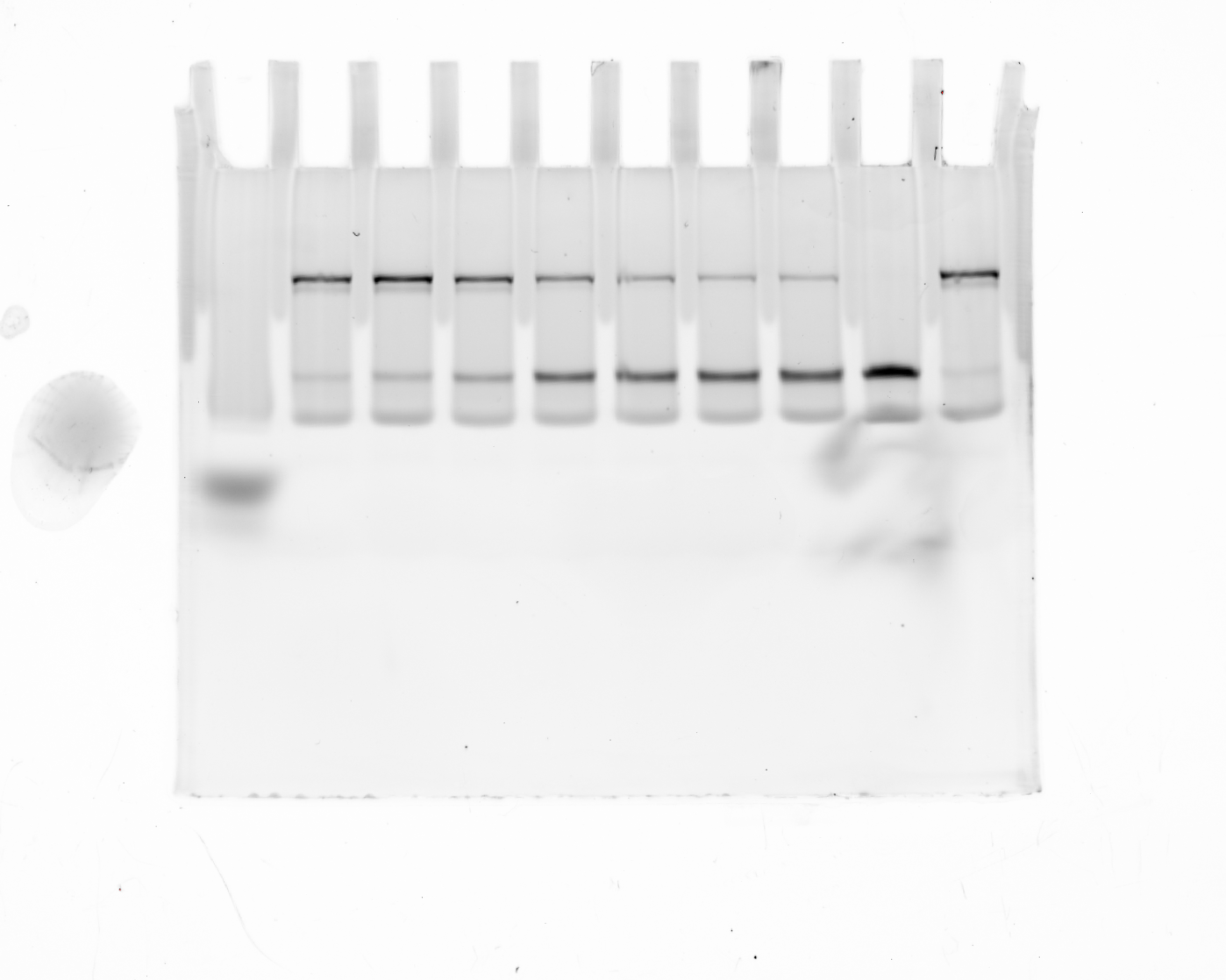

Supplement: Supplementary file 7 — Source Data Fig. 7 [file 44319_2023_44_MOESM7_ESM.zip › Source_Data_Figure_7/Panel_B/Figure_7_Panel_B_Helicase_Assay_FANCJ_AALA.tif]

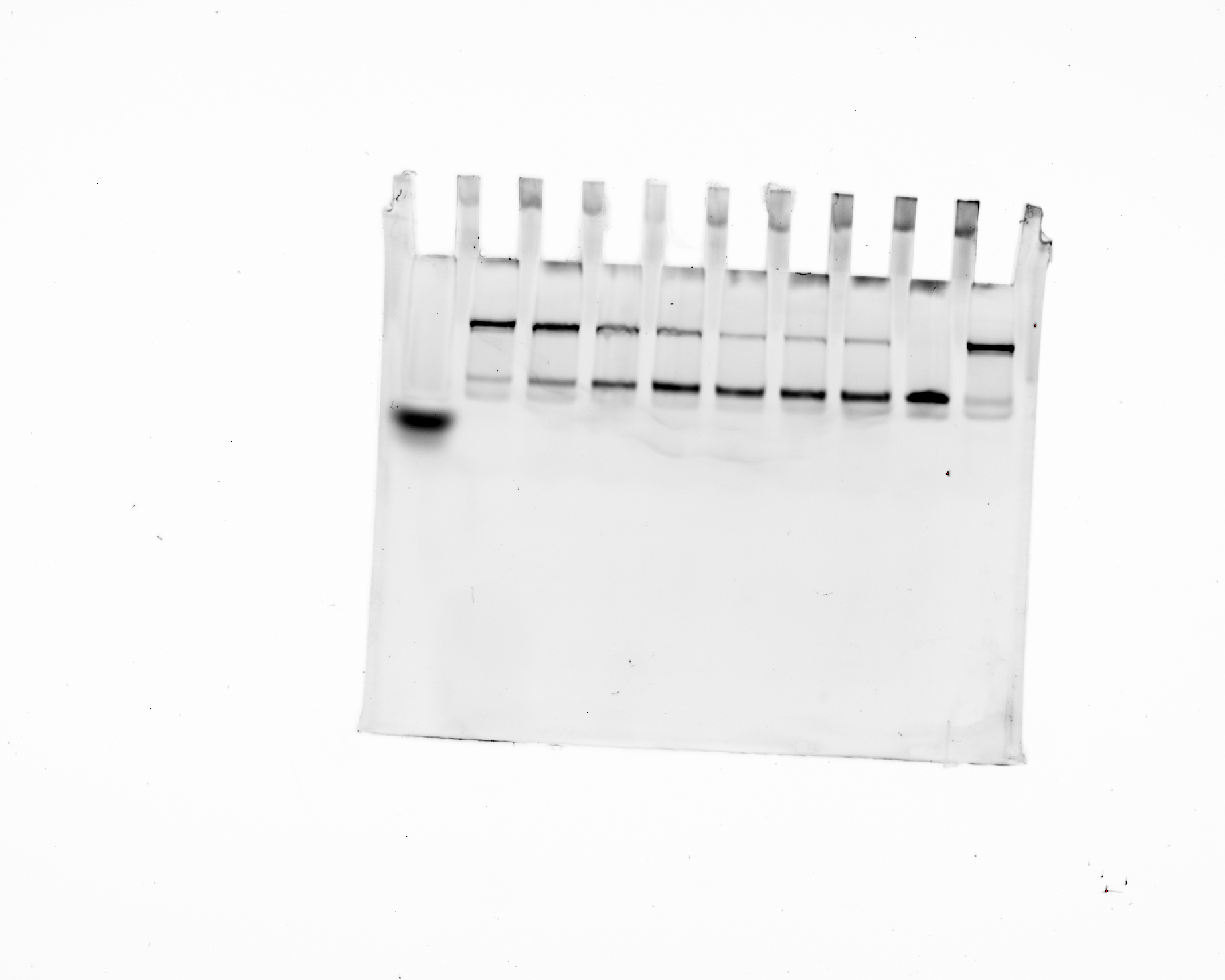

Supplement: Supplementary file 7 — Source Data Fig. 7 [file 44319_2023_44_MOESM7_ESM.zip › Source_Data_Figure_7/Panel_B/Figure_7_Panel_B_Helicase Assay_FANCJ WT.tif]

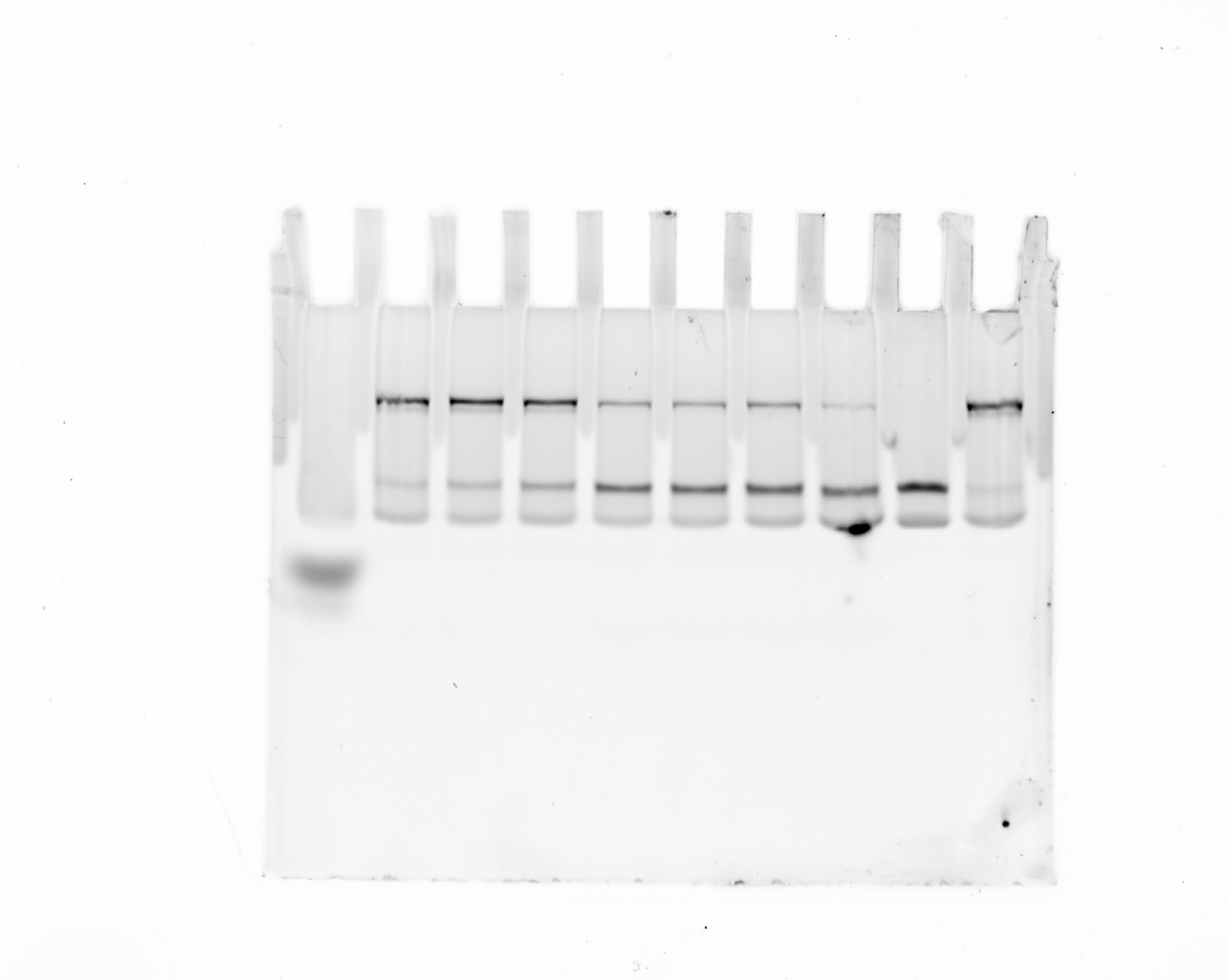

Supplement: Supplementary file 7 — Source Data Fig. 7 [file 44319_2023_44_MOESM7_ESM.zip › Source_Data_Figure_7/Panel_B/Figure_7_Panel_B_Helicase Assay FANCJ N734H.tif]

## Slide 1
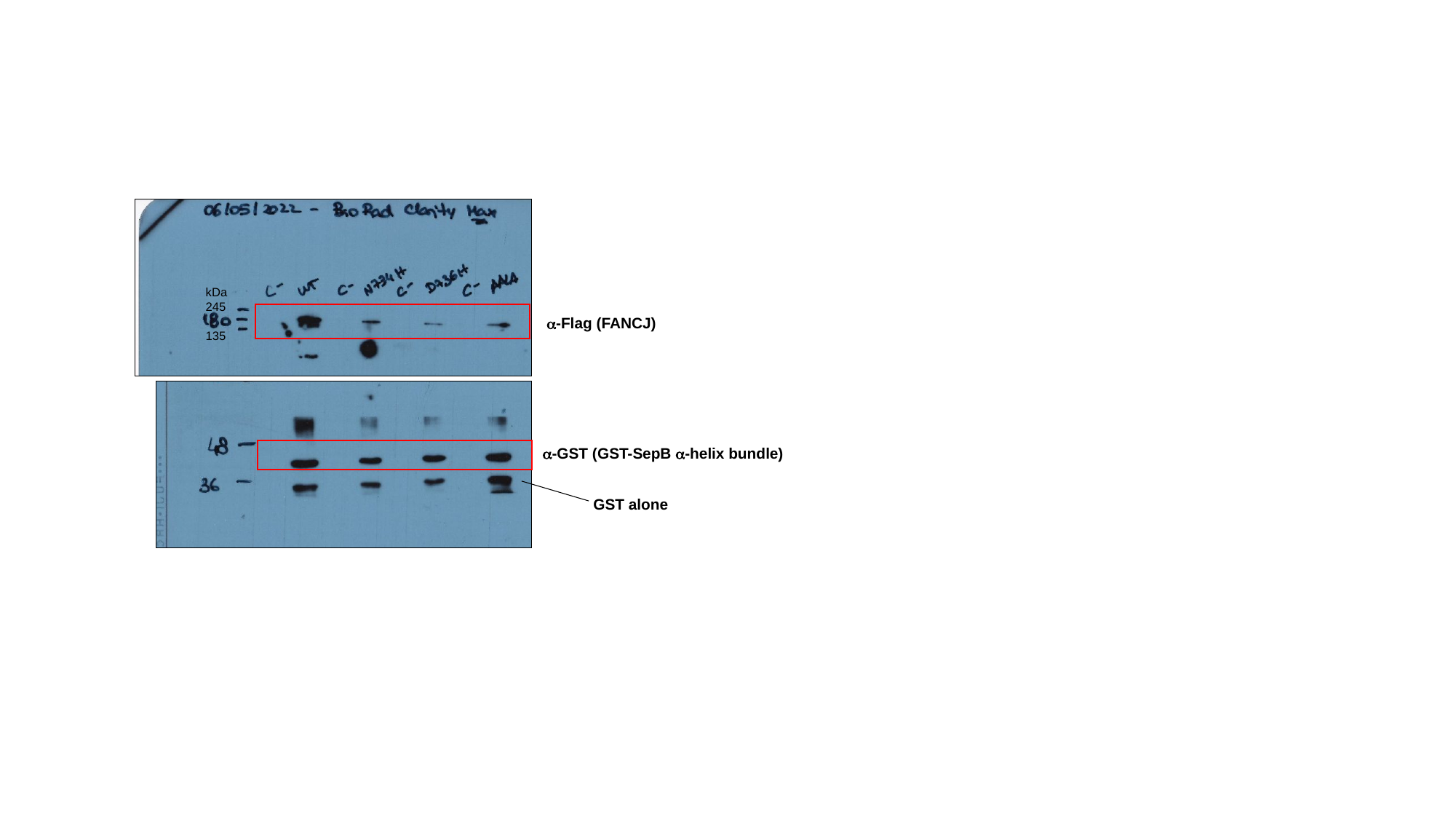

kDa
245
135
a-Flag (FANCJ)
a-GST (GST-SepB α-helix bundle)
GST alone

Supplement: Supplementary file 7 — Source Data Fig. 7 [file 44319_2023_44_MOESM7_ESM.zip › Source_Data_Figure_7/Panel_A/Figure_7_Panel_A_WB.pptx]
