# Supplementary material for: FANCJ DNA helicase is recruited to the replisome by AND-1 to ensure genome stability
Source: EMBO Rep. 2024 Jan 2;25(2):24. doi: 10.1038/s44319-023-00044-y (PMC10897178; doi:10.1038/s44319-023-00044-y)
Supplement: Supplementary file 4 — Source Data Fig. 4 [file 44319_2023_44_MOESM4_ESM.zip › Source_Data_Figure_4/Panel_A/Figure 4_Panel A_WB.pptx]

## Slide 1
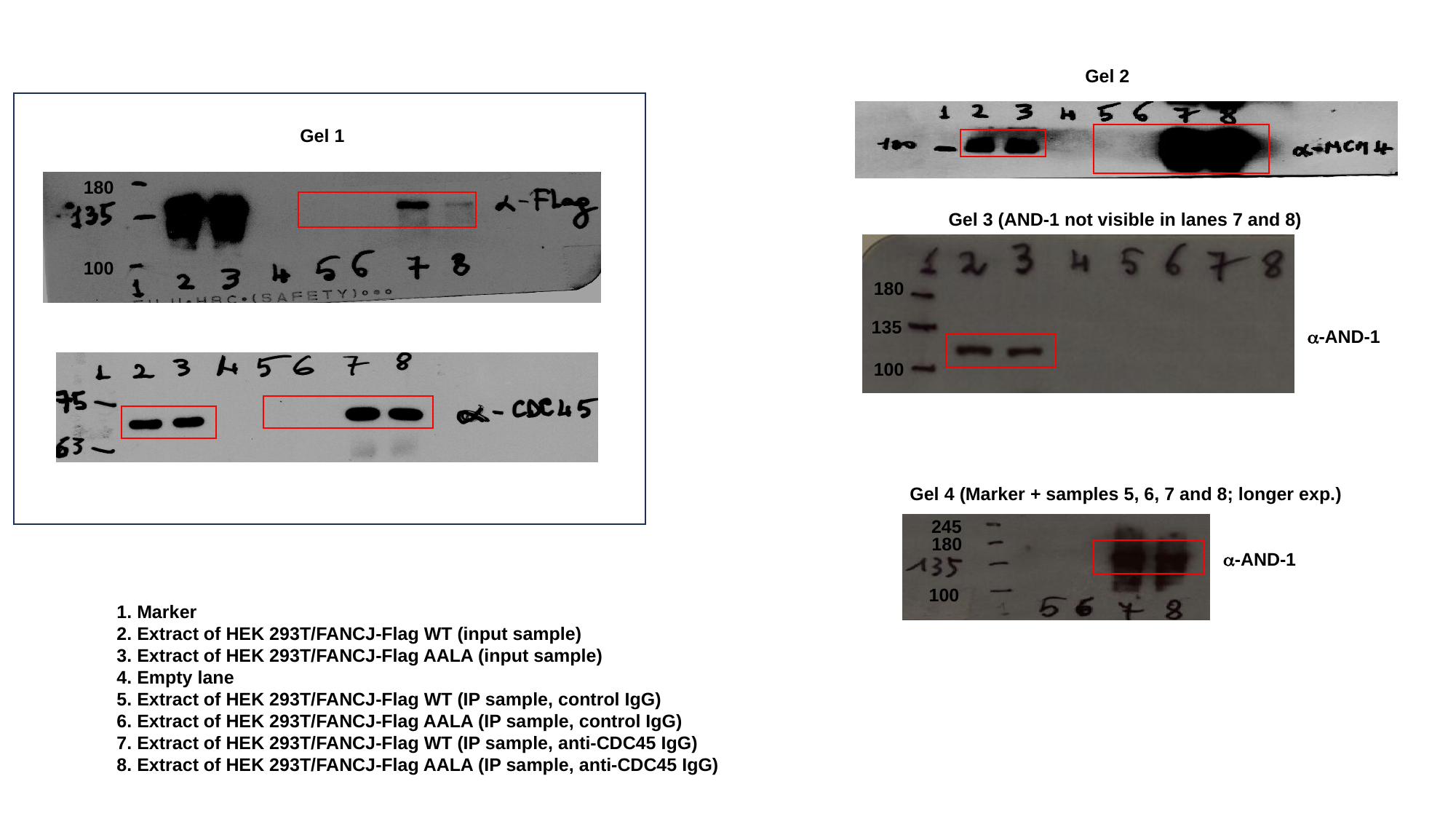

Gel 2
Gel 1
180
Gel 3 (AND-1 not visible in lanes 7 and 8)
180
135
a-AND-1
100
100
Gel 4 (Marker + samples 5, 6, 7 and 8; longer exp.)
245
180
a-AND-1
100
Marker
Extract of HEK 293T/FANCJ-Flag WT (input sample)
Extract of HEK 293T/FANCJ-Flag AALA (input sample)
Empty lane
Extract of HEK 293T/FANCJ-Flag WT (IP sample, control IgG)
Extract of HEK 293T/FANCJ-Flag AALA (IP sample, control IgG)
Extract of HEK 293T/FANCJ-Flag WT (IP sample, anti-CDC45 IgG)
Extract of HEK 293T/FANCJ-Flag AALA (IP sample, anti-CDC45 IgG)
