# Supplementary material for: FANCJ DNA helicase is recruited to the replisome by AND-1 to ensure genome stability
Source: EMBO Rep. 2024 Jan 2;25(2):24. doi: 10.1038/s44319-023-00044-y (PMC10897178; doi:10.1038/s44319-023-00044-y)
Supplement: Supplementary file 6 — Source Data Fig. 6 [file 44319_2023_44_MOESM6_ESM.zip › Source_Data_Figure_6/Panel_B/Figure_6_Panel_B_WB.pptx]

## Slide 1
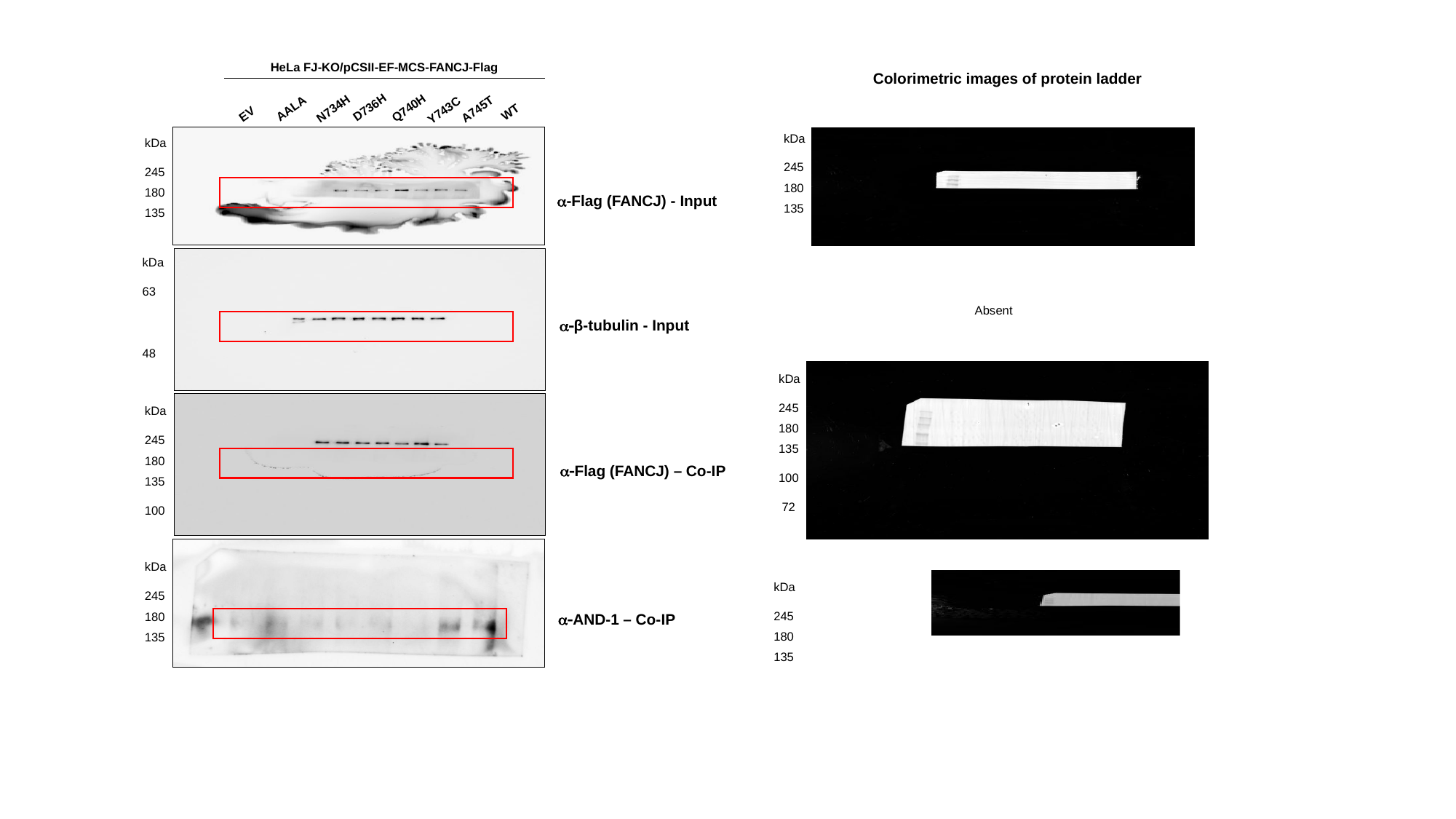

HeLa FJ-KO/pCSII-EF-MCS-FANCJ-Flag
Colorimetric images of protein ladder
AALA
D736H
Q740H
N734H
A745T
Y743C
WT
EV
kDa
245
180
135
kDa
245
180
135
a-Flag (FANCJ) - Input
kDa
63
48
Absent
a-β-tubulin - Input
kDa
245
180
135
100
 72
kDa
245
180
135
100
a-Flag (FANCJ) – Co-IP
kDa
245
180
135
kDa
245
180
135
a-AND-1 – Co-IP
