# Supplementary material for: FANCJ DNA helicase is recruited to the replisome by AND-1 to ensure genome stability
Source: EMBO Rep. 2024 Jan 2;25(2):24. doi: 10.1038/s44319-023-00044-y (PMC10897178; doi:10.1038/s44319-023-00044-y)
Supplement: Supplementary file 9 — Expanded View Figures [file 44319_2023_44_MOESM9_ESM.pdf]

Expanded View Figures

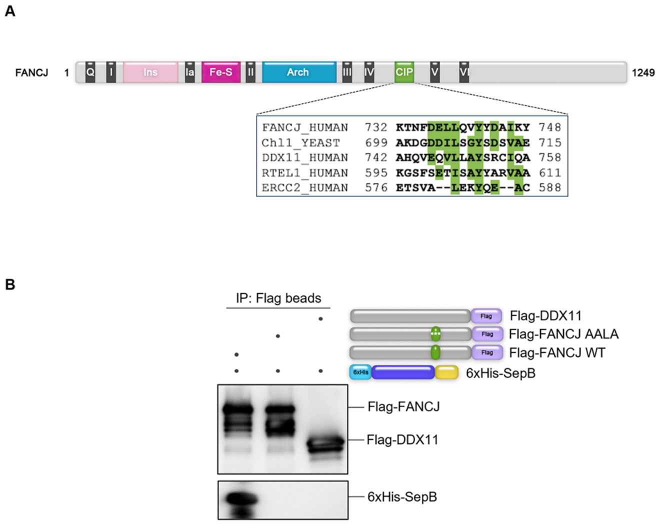

Figure EV1. FANCJ, but not DDX11, directly interacts with AND-1 6xHis-SepB.

(**A**) Schematic representation of the polypeptide chain of human FANCJ. Conserved sequence motifs and domains are indicated with the same abbreviations and colors used in Fig. 1A. In the insert, alignment of human FANCJ and budding yeast Chl1 CIP box sequence is reported with corresponding regions of other SF2 Fe–S human DNA helicases. Abbreviations used are *HUMAN*, *Homo sapiens* and *YEAST, Saccharomyces cerevisiae*. The KALIGN tool (version 3.3.1) was used. Highly conserved amino-acid residues are highlighted in green. (**B**) Co-pull-down experiments of Flag-tagged FANCJ WT or AALA mutant and AND-1 6xHis-SepB using anti-Flag agarose beads. Pulled-down samples were analyzed by Western blot with an anti-Flag peroxidase-conjugated mouse monoclonal antibody (Sigma-Aldrich cat. A8592) and anti-poly-Histidine peroxidase-conjugated mouse monoclonal antibody (Sigma-Aldrich cat. A7058). Schematic of the recombinant proteins used in the pulled-down experiments is shown.

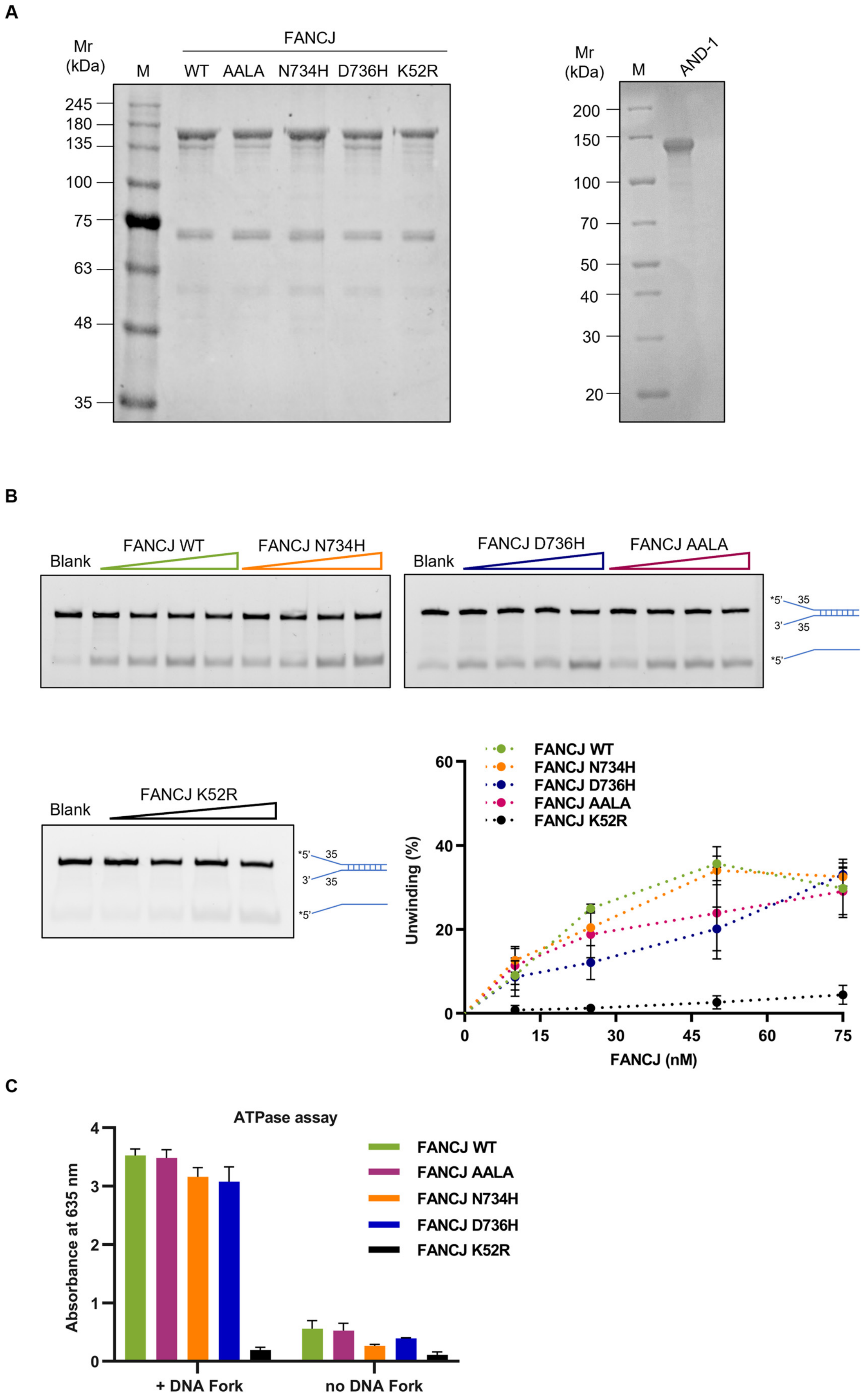
A
Mr (kDa)
M
FANCJ
WT
AALA
N734H
D736H
K52R
245
180
135
100
75
63
48
35
Mr (kDa)
M
AND-1
200
150
100
70
50
40
30
20
B
Blank
FANCJ WT
FANCJ N734H
Blank
FANCJ D736H
FANCJ AALA
*5'
35
3'
35
*5'
Blank
FANCJ K52R
FANCJ WT
FANCJ N734H
FANCJ D736H
FANCJ AALA
FANCJ K52R
Unwinding (%)
FANCJ (nM)
0
15
30
45
60
75
20
40
60
C
ATPase assay
Absorbance at 635 nm
0
1
2
3
4
FANCJ WT
FANCJ AALA
FANCJ N734H
FANCJ D736H
FANCJ K52R
+ DNA Fork
no DNA Fork

 © The Author(s)

Figure EV2. Biochemical analysis of CIP box FANCJ mutant derivatives.

(**A**) SDS-PAGE analysis of purified recombinant FANCJ WT and indicated mutants and AND-1 full-length protein. An aliquot of each protein batch (3 μg) was loaded onto the indicated gel lane. Gels were stained with ProBlue Safe Stain (GiottoBiotech). Size of protein markers, loaded onto the lane indicated with *M*, is reported on the left. (**B**) DNA helicase assays using a forked duplex fluorescent-labeled DNA substrate. Asterisk represents the fluorophore attached to the 5′-end of the DNA oligonucleotide, named Fluo-D1-35. Blank refers to a control assay without protein. Data plot derives from three independent experiments (mean ± SD). (**C**) ATPase assays were carried out using the indicated FANCJ proteins (100 nM) with/without the DNA fork ligand, as described in "Methods". Values reported derive from three independent experiments (mean ± SD).

© The Author(s)

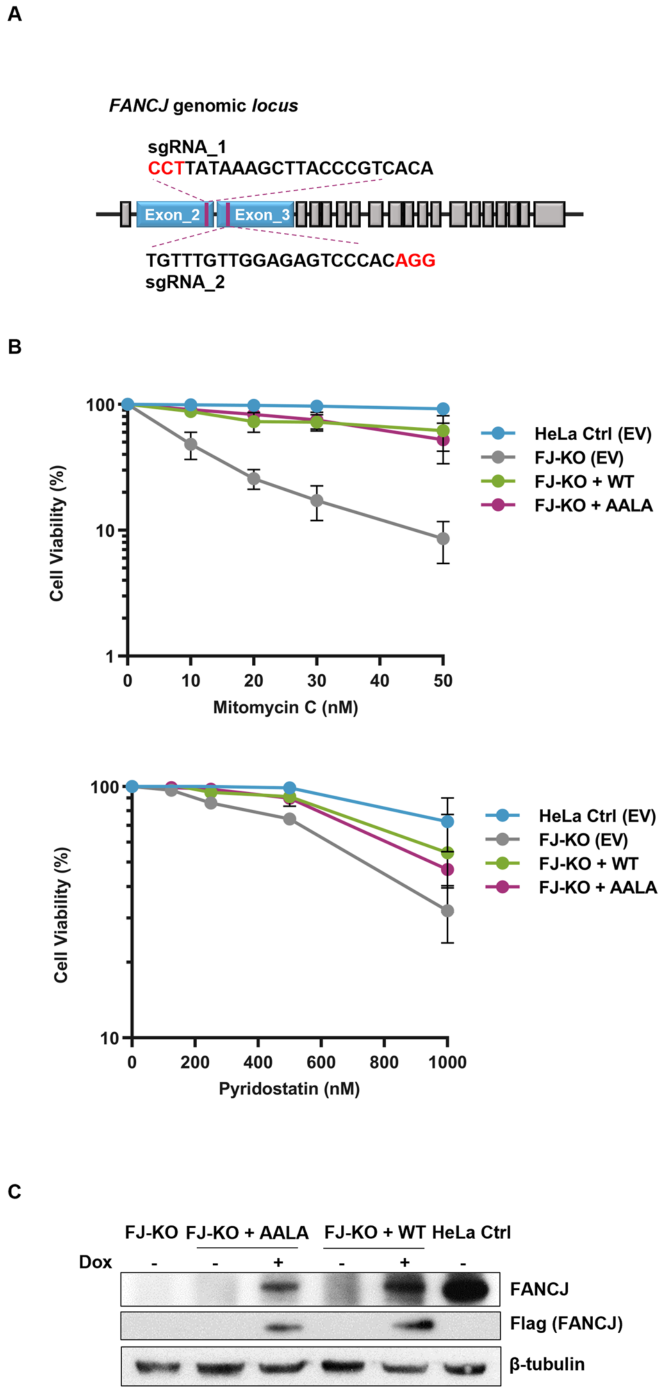

Figure EV3. Establishment of a FJ-KO HeLa cell line and its complementation by *FANCJ* WT and AALA mutant alleles.

(**A**) Schematic representation of the human *FANCJ* genomic *locus*. The sequence of CRISPR-paired guide RNAs targeting exons 2 and 3 is reported. *PAM* sequence is highlighted in red. (**B**) Viability assays of FJ-KO HeLa cell lines complemented with FANCJ WT and the AALA mutant. HeLa cells (*Ctrl*) or FJ-KO cells, transduced with lentiviral particles deriving from plasmid pCSII-EF-MCS-FANCJ WT or -FANCJ AALA or the empty vector (*EV*), were treated for 5 days with the indicated concentrations of MMC and PDS ($n = 3$ biologically independent experiments, mean ± SD). Cells were detected by crystal violet staining, as described in "Methods". Paired *t* tests were performed to analyze statistically significant differences between the HeLa control and the complemented cell lines, but no differences were found for both viability assays. (**C**) FJ-KO HeLa cell lines were established that stably express Flag-tagged FANCJ WT or the AALA mutant under the control of a Tetracycline-responsive promoter. Expression of ectopic *FANCJ* was detected before and after induction with Doxycycline (1 μg/mL; *Dox*) by Western blot analysis of whole extracts from the indicated cell lines using an anti-FANCJ or anti-Flag antibody. β-tubulin was used as a loading control.

© The Author(s)

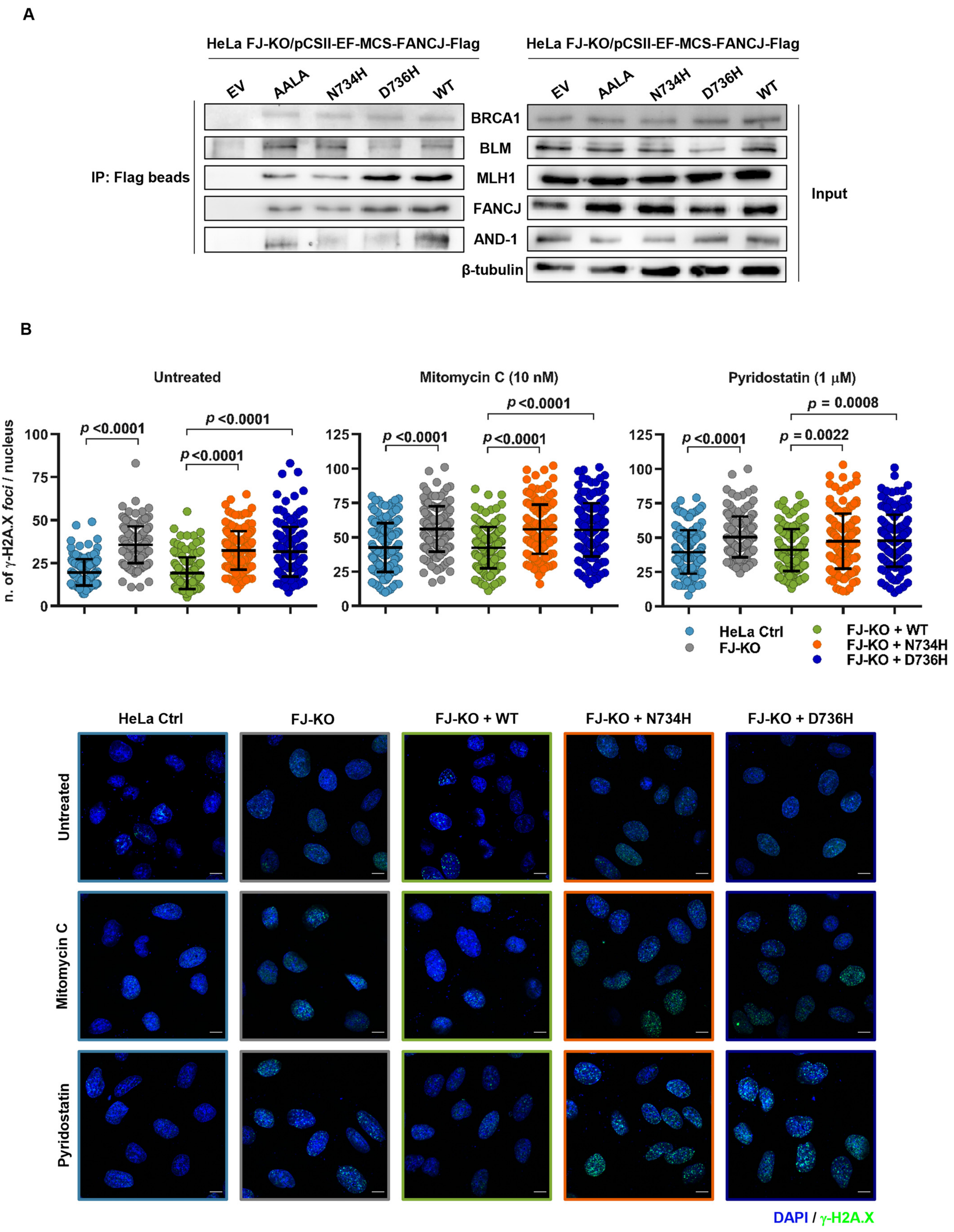
A
HeLa FJ-KO/pCSII-EF-MCS-FANCJ-Flag
HeLa FJ-KO/pCSII-EF-MCS-FANCJ-Flag
EV
AALA
N734H
D736H
WT
IP: Flag beads
BRCA1
BLM
MLH1
FANCJ
AND-1
β-tubulin
Input
B
Untreated
Mitomycin C (10 nM)
Pyridostatin (1 μM)
n. of γ-H2A.X foci / nucleus
p <0.0001
p = 0.0008
p = 0.0022
HeLa Ctrl
FJ-KO
FJ-KO + WT
FJ-KO + N734H
FJ-KO + D736H
Untreated
Mitomycin C
Pyridostatin
DAPI / γ-H2A.X

Figure EV4. FANCJ CIP box mutants, AALA, N734H and D736H retain the ability to interact with BRCA1, BLM and MLH1 and induce enhanced DNA damage.

(**A**) Co-immunoprecipitation experiments with anti-Flag agarose beads were carried out on whole extracts of HeLa FJ-KO cells transiently transfected with pCSII-EF-MCS vector expressing Flag-tagged FANCJ wild type (*WT*) or the indicated mutants (*EV* stands for empty vector). Western blot analysis was carried out on the input (0.6% of each sample; 10 µg of total protein) and pulled-down material (50% of each sample). Proteins of interest were detected by Western blot experiments using the indicated antibodies. Experiments were done in triplicate. (**B**) HeLa FJ-KO cells, transfected with plasmid vectors expressing FANCJ WT or the indicated mutants, were treated with PDS or MMC. γ-H2A.X *focus* formation was detected by immunofluorescence with a monoclonal antibody that specifically recognizes the Ser139-phosphorylated form of the above histone. Scale bar, 10 µm. Dot plot of the number of *foci* detected per cell is reported in each graph. Bars indicate mean ± SD; 150 cells were analyzed per condition; $n = 2$ biologically independent experiments, with at least two technical replicates per experiment; two-tailed *P* value ($P < 0.01$) was calculated using Student's t test nonparametric for unpaired data with Welch correction.

 © The Author(s)
